# Supplementary material for: Design and Rationale of the Sevoflurane for Sedation in Acute Respiratory Distress Syndrome (SESAR) Randomized Controlled Trial
Source: J Clin Med. 2022 May 16;11(10):2796. doi: 10.3390/jcm11102796 (PMC9147018; doi:10.3390/jcm11102796)
Supplement: Supplementary file 1 [file jcm-11-02796-s001.zip › Supplementary Material File S1_Final__SESAR trial protocol & approval_v9_20211117.pdf]

Supplementary Material File S1. Complete protocol of the Sevoflurane for Sedation in ARDS (SESAR) trial (version 9, approved on February 10, 2022)

Sevoflurane for Sedation in Acute Respiratory Distress Syndrome: A Multicenter Prospective Randomized Trial

Acronym: ***SESAR Study*** *(SEvoflurane for Sedation in ARds)*

Version: 9 **Date:** 17/11/2021

Sponsor code	EudraCT identifying number
**RBHP 2018 JABAUDON**	2018-000763-83

Study sponsor

Centre Hospitalier Universitaire (CHU) Clermont-Ferrand
58, Rue de Montalembert
63003 Clermont-Ferrand Cedex 1

Principal coordinator

Matthieu JABAUDON, MD, PhD
Department of Perioperative Medicine, CHU Clermont-Ferrand
Université Clermont Auvergne, CNRS UMR 6293, INSERM U1103, GReD
1, Place Lucie et Raymond Aubrac, 63003 Clermont-Ferrand Cedex 1
Email: mjabaudon@chu-clermontferrand.fr
Tel: +33 (0)4 73 750 501 / Fax: +33 (0)4 73 750 500

Study methodology

Bruno PEREIRA, PhD Biostatistics
Biostatistical Unit, Department of Clinical Research and Innovation (DRCI)
CHU Clermont-Ferrand
58, Rue Montalembert, 63003 Clermont-Ferrand Cedex 1
Email: bpereira@chu-clermontferrand.fr

SUMMARY

**Title**	Sevoflurane for Sedation in Acute Respiratory Distress Syndrome: A Multicenter Prospective Randomized Trial
**Context and rationale**	Acute respiratory distress syndrome (ARDS) represents approximately 10% of intensive care unit (ICU) admissions and more than 20% of patients requiring mechanical ventilation, with a hospital mortality rate of 35-45%[1,2]. Its pathophysiological landmark, diffuse alveolar damage, is associated with alveolar inflammation, epithelial injury and impaired alveolar fluid clearance (AFC)[3,4]. Despite intense research and advances in terms of limiting mechanical injury from ventilation (e.g., with the use of lower tidal volumes[5]), the identification of a single, targeted, effective ARDS pharmacological therapy has failed to date[6], and ARDS is still a deadly condition for patients and a serious challenge for clinicians[2].<br>Several preclinical studies have shown that a volatile anesthetic agent such as inhaled sevoflurane improves gas exchange[7–9], reduces alveolar edema[9] and attenuates pulmonary and systemic inflammation[10,11] in experimental models of ARDS. These effects could be explained by restored lung epithelial function and by immunomodulatory effects of sevoflurane. Volatile anesthetic agent use in the ICU, aided by technological advances, has now become more accessible to critical care physicians[12]. With increasing concern over adverse patient consequences associated with our current sedation practice, there is growing interest to find non–benzodiazepine-based alternative sedatives.<br>Research has demonstrated that volatile-based sedation may provide superior awakening and extubation times in comparison with current intravenous sedation agents (propofol and benzodiazepines such as midazolam)[13,14]. Volatile agents such as sevoflurane may also possess important end-organ protective properties mediated via cytoprotective and anti-inflammatory mechanisms[15].<br>In a previous pilot randomized controlled trial, our group found that, in patients with moderate-severe ARDS, the use of inhaled sevoflurane improved oxygenation and decreased levels of a marker of lung epithelial injury (soluble receptor for advanced glycation end-products, sRAGE) and of some inflammatory markers (interleukin (IL)-1β, IL-6, IL-8 and tumor necrosis factor (TNF)-α), compared to intravenous midazolam[16]. These results reinforce those from previously published preclinical studies as they suggest a protective effect of sevoflurane from alveolar/systemic inflammation and from reduced epithelial injury and/or improved AFC, as assessed by plasma sRAGE[17]. In this study, as in others from our group[18–20], sevoflurane inhalation through dedicated device was well tolerated, with no major adverse effect, e.g. on renal function or respiratory mechanics.<br>However, this first study of inhaled sevoflurane in patients with ARDS was underpowered to evaluate mortality or other major clinical outcomes[16]. Thus, the benefits and risks of inhaled sevoflurane for sedation in ARDS require further evaluation. In addition, it remains unknown whether specific ARDS subphenotypes might better benefit from inhaled sevoflurane, and which clinical and biological features, different natural histories and differential responses to therapy (also known as "endotypes") may be capable of predicting therapeutic response, and not just prognosis[17,21–25].<br>We hypothesized that a strategy of inhaled sedation with sevoflurane could

	be more effective at reducing organ failure, healthcare utilization, morbidity and mortality in patients with moderate-severe ARDS than a strategy of intravenous sedation. Given the number of ICU patients with ARDS receiving sedation, and the overall burden of ARDS on healthcare, the study could have significant clinical importance and could be highly feasible.
**Objectives**	Main objective:<br>To assess the efficacy of a sedation with inhaled sevoflurane in improving a composite outcome of mortality and time off the ventilator at 28 days in patients with moderate-severe ARDS in comparison to a control group receiving intravenous sedation with propofol.<br><br>Secondary objectives:<br>To evaluate the safety (clinical adverse events) of the two sedation strategies<br>To describe the effects of inhaled sevoflurane, compared to intravenous sedation, on respiratory mechanics<br>To describe the effects of inhaled sevoflurane, compared to intravenous sedation, on gas exchange and physiologic measures<br>To describe the effects of inhaled sevoflurane, compared to intravenous sedation, on hemodynamic measures and renal function (KDIGO criteria for acute kidney injury[26])<br>To describe the effects of inhaled sevoflurane, compared to intravenous sedation, on duration of mechanical ventilation<br>To describe the effects of inhaled sevoflurane, compared to intravenous sedation, on organ dysfunction<br>To describe the effects of inhaled sevoflurane, compared to intravenous sedation, on the use of rescue procedures (e.g., nitric oxide, epoprostenol sodium, high frequency ventilation, extracorporeal membrane oxygenation (ECMO), and neuromuscular blockade use after 48h from randomization)<br>To describe the effects of inhaled sevoflurane, compared to intravenous sedation, on ICU-acquired delirium<br>To describe the effects of inhaled sevoflurane, compared to intravenous sedation, on long-term outcomes (e.g., disability, health-related quality of life, self-rated health, pain-interference, post-traumatic stress-like symptoms, cognitive function, subsequent return to work and healthcare use)<br>To assemble a biological collection of plasma, alveolar, and urine samples for future mechanistic and endotyping studies of the biological effects of sevoflurane<br>To assess the presence of subphenotypes among patients with ARDS, based on distinct clinical, imaging[25,27,28], and/or biological[21,22] profiles (endotypes), and their differential therapeutic response to sevoflurane, if any<br>To assess between-group healthcare-related costs during ICU stay and hospital stay (ancillary analysis)<br>To assess the specific effects of inhaled sevoflurane in patients with Covid-19-related ARDS
**Primary outcome measure**	The primary outcome (event of interest) is the number of days alive and off the ventilator at 28 days (ventilator-free days through day 28, VFD28),

	thereby considering death as a competing event.
**Study design**	An investigator-initiated, multicenter, prospective, randomized, stratified, parallel-group clinical trial with blinded outcome assessment and concealed allocation of patients with moderate-to-severe ARDS to a strategy of inhaled sedation with sevoflurane or to a strategy of current intravenous sedation practice using propofol.
**Number of centers**	N= 37<br>Since "version 7 (Covid-19)" of the SESAR trial and because the training of all centers will not be feasible in practice due to the recent self-confinement measures related to the current context of widespread disease resulting from the 2019 coronavirus pandemics (Covid-19), the trial will first start in centers with previous experience in the clinical use of inhaled sedation in critically ill patients (N=14). In this Covid-19 pandemics situation, the start and conduct of the trial will be managed remotely by the sponsor (CHU Clermont-Ferrand) and the coordinating investigator, including the official study implementation at each participating center, in accordance with the latest guidance of the Agency (ANSM) issued on March 20, 2020 (www.ansm.sante.fr/Activites/Essais-cliniques/Covid-19-Essais-cliniques-en-cours).<br>Other centers will be trained and allowed to participate only when the situation will allow it.
**Inclusion criteria**	1. Age ≥18 years<br>2. Presence for ≤24 hours of all of the following conditions, within one week of a clinical insult or new or worsening respiratory symptoms:<br>a. PaO_2/FiO_2 <150 mmHg with positive end-expiratory pressure (PEEP) ≥8 cmH_2O[i,ii,iii]<br>or, if arterial blood gas not available<br>SpO_2/FiO_2 ratio that is equivalent to a PaO_2/FiO_2 <150 mmHg with PEEP ≥8 cmH_2O (Appendix A1), and a confirmatory SpO_2/FiO_2 ratio between 1-6 hours after the initial SpO_2/FiO_2 ratio determination[iii,iv]<br>b. Bilateral opacities not fully explained by effusions, lobar/lung collapse, or nodules<br>c. Respiratory failure not fully explained by cardiac failure or fluid overload; need objective assessment (e.g., echocardiography) to exclude hydrostatic edema if no risk factor present<br>i. If altitude >1,000 m, then PaO_2/FiO_2 <150 x (PB/760).<br>ii. These inclusion criteria ensure a non-transient, established hypoxia that persists despite elevated PEEP and time. Initial, post-intubation, PEEP is typically <8 cmH_2O.<br>iii. The qualifying PaO_2/FiO_2 or the SpO_2/FiO_2 must be from intubated patients receiving at least 8 cmH_2O PEEP.<br>iv. When hypoxia is documented using pulse oximetry, a confirmatory SpO_2/FiO_2 ratio is required to further establish persistent hypoxia. Qualifying SpO_2/FiO_2 must use SpO_2 values less than or equal to 96% Qualifying SpO_2 must be measured at least 10 minutes after any change to FiO_2.<br>The 24-hour enrollment time window begins when criteria a-c are met. Criteria may be met at either the Network or referring hospital.

	The first qualifying SpO_2/FiO_2 (not the confirmatory SpO_2/FiO_2) is used to determine this time window.
**Non-inclusion criteria**	Absence of affiliation to the French *Sécurité Sociale*<br>Patient under a tutelage measure or placed under judicial protection<br>Continuous sedation with inhaled sevoflurane at enrollment<br>Known pregnancy<br>Currently receiving ECMO therapy<br>Chronic respiratory failure defined as $PaCO_2$ >60 mmHg in the outpatient setting<br>Home mechanical ventilation (non-invasive ventilation or via tracheotomy) except for CPAP/BIPAP used solely for sleep-disordered breathing<br>Body mass index >40 kg/m^2<br>Chronic liver disease defined as a Child-Pugh score of 12-15 (Appendix A2)<br>Expected duration of mechanical ventilation <48 hours<br>Tidal volume of 6 mL/kg predicted body weight (PBW) below 200 mL<br>Moribund patient, i.e. not expected to survive 24 hours despite intensive care<br>Burns >70% total body surface<br>Previous hypersensitivity or anaphylactic reaction to sevoflurane or cisatracurium<br>Medical history of malignant hyperthermia<br>Long QT syndrome at risk of arrhythmic events<br>Medical history of liver disease attributed to previous exposure to a halogenated agent (including sevoflurane)<br>Known hypersensitivity to propofol or any of its components<br>Known allergy to eggs, egg products, soybeans, and soy products<br>Suspected or proven intracranial hypertension<br>Enrollment in another interventional ARDS trial with direct impact on sedation and mechanical ventilation<br>Endotracheal ventilation for greater than 120 hours (5 days)<br>Persistent bronchopleural fistula despite chest tube drainage<br>PaO_2/FiO_2 (if available) >200 mmHg after meeting inclusion criteria and before randomization<br>*As oxygenation may improve during the 24-hour enrollment window, this exclusion criterion ensures that patients with mild ARDS are not included in the study.*<br>Pregnancy testing will be systematically performed to rule out pregnancy in female patients of reproductive age, as per current practice in all participating centers.
**Interventions**	Patients will be randomly assigned to one of the two study groups using a dedicated, password-protected, SSL-encrypted website:<br>- Inhaled sedation with sevoflurane, as vaporized via the Anesthesia Conserving Device (AnaConDa-S, Sedana Medical, Danderyd, Sweden).<br>- Intravenous sedation with propofol, as already routinely used in participating ICUs.<br>In both groups, and in accordance with current guidelines on the management of severe ARDS, patients will receive cisatracurium besylate for neuromuscular blockade, and deep sedation (inhaled or intravenous,

	depending on the randomization group) will be protocolized to target a Richmond Agitation-Sedation Scale (RASS) of -4 to -5 (Ramsay of 5-6, or Riker of 1-2) before starting, and during, the cisatracurium besylate infusion. The cisatracurium besylate infusion will be continued until PaO_2/FiO_2 exceeds 150 mmHg for 4 hours with FiO_2 <0.6[29,30]; then, light sedation will be targeted (RASS of 0 to -1, Ramsay of 2-3, or Riker of 3-4), with prompt sedation interruption whenever possible. Higher doses of sedation (inhaled or intravenous, depending on the randomization group) will be allowed for respiratory distress, ventilator dyssynchrony, or hypoxia.<br>We will protocolize low tidal volume (4-8 mL/kg PBW) ventilation with higher rather than lower levels of PEEP[31], as recommended in moderate-severe ARDS[32], and the strategy for weaning from mechanical ventilation, including spontaneous breathing trials, in both arms.<br>Whenever possible, we will recommend sites to wait at least 12 hours before proning, as in the PROSEVA study[29].<br>We will provide recommendations for conservative fluid management in both arms.<br>To help match the two groups and address potential inter-hospital differences, <u>randomization will be stratified</u> by institution, by the degree of ARDS severity (PaO_2/FiO_2 <100 mmHg), by the suspicion or presence of Covid-19 as a cause for ARDS, and by the presence of shock (defined as intravenous infusion of vasoactive drugs) at study entry.<br>Patients will be assessed at least once a day during the first week after randomization, then on days 14, 21, 28, 90 and 365, as described in the Time-Events schedule (Appendix B). Long-term outcomes such as disability, health-related quality of life, self-rated health, post-traumatic stress-like symptoms, and cognitive function, subsequent return to work and healthcare use will be assessed at 3 and 12 months.<br>In the current Covid-19 pandemic context, data will be collected remotely from the (electronic) patient health record by clinical research associates at each center, so that 1) the risk for intensive care providers to be contaminated with the virus is minimized, as no data will be collected at patient bedside, and 2) no additional work overload will be needed from intensive care providers, as their staffing may be very challenged in this threatening situation. Relevant information on Covid-19, such as specific interventions that may be delivered in these patients (such as antiviral, anti-inflammatory (e.g., corticosteroids), immunomodulatory and/or other drug(s)) will be collected.<br>This strategy will allow the rigorous collection of most data relevant to major patient outcomes (survival, duration of mechanical ventilation, indices of lung function, and main safety data) while ensuring the safety of both intensive care providers and clinical research associates, and preventing interaction to the priority care patients will receive in participating ICUs.<br>However, and as a result, a larger amount of missing data can be expected for some variables that may be considered less relevant to collect from the (electronic) patient health record in the current pandemic context due to the surge in patients with Covid-19.
**Number of subjects**	The following assumptions were made:<br>- The variability of days free from ventilation would follow the properties of recently published studies:

	- a pilot study from our group: median [interquartile range], 13 [1–20] and 5 [0–28] VFDs at day 28 in patients receiving inhaled sedation with sevoflurane and those receiving intravenous sedation, respectively[16].<br>- a recent multicenter randomized controlled trial of mechanical ventilation targeting transpulmonary pressure (EPVent-2 trial) (22 [15-24] and 21 [16.5-24] VFDs at day 28 in the intervention (n=102) and control (n=98) groups, respectively)[33]<br>- a recent multicenter randomized controlled trial of early neuromuscular blockade (ROSE trial) (9.6 ± 10.4 and 9.9 ± 10.9 VFDs at day 28 in the intervention (n=501) and control (n=505) groups, respectively)[34]<br>- 28-day mortality would be around 30-35%, based on data from recent ARDS trials[33–35]<br><br>To highlight a minimal, but clinically relevant, difference of 2 days free from ventilation at day 28 for a standard-deviation at 8[36,37], a two-sided type I error at 5%, and a statistical power greater than 80%, we have estimated that 340 patients by group would be necessary. We therefore propose to include 700 patients (350 by group).<br>An interim analysis is planned after 350 patients (symmetric group sequential flexible stopping boundaries as described by Lan and DeMets[38]). A data monitoring and safety committee (DMSC) will be convened to discuss the continuation of this study in case of a between-group difference in severe adverse events (SAEs) or suspected unexpected severe adverse events (SUSAEs).<br>Interim safety reports to the DMSC will be performed each time 40 patients (20 by group) are enrolled, according to previous results published by our team[16], and will include blinded variables on randomization strata (inclusions by center, number of patients enrolled with Covid-19, PaO_2/FiO_2 <100 mmHg or shock at study entry), SAEs, and the rates and causes of death at day 28 in both groups.<br>The DMSC will be empowered to communicate these interim findings directly to sponsor (CHU Clermont-Ferrand) and public health authorities if the committee thinks the trial produces any results that may be of relevance to public health with regard to the current Covid-19 outbreak. At the end of the Covid-19 pandemics, we will check if the results obtained in patients enrolled during the pandemics do not alter the initial assumptions for sample size estimation. Standardized mean (or median) differences and statistical power will then be estimated.
**Study schedule**	Anticipated duration of the recruitment period: 36 months<br>Duration of participation of each patient: 365 days<br>Total duration of the study: 48 months
**Expected patient or public health benefit**	See the accompanying “NOTICE - SESAR trial v5 (Covid-19)” document for the specific impact the trial could have during the Covid-19 pandemics.<br><br>Recent findings from a large international observational study emphasized that ARDS is still a frequent and deadly condition. ARDS represents more than 10% of ICU admissions and nearly 25% of ICU patients requiring mechanical ventilation, with a hospital mortality rate of 35-45%[2]. Beyond such a high mortality rate, ARDS is associated with greater healthcare

	utilization, reduced quality of life among survivors and worse long-term physical and cognitive outcomes[39–46]. Fifty years after its first clinical description[47], and despite intense research, the identification of an effective ARDS therapy has failed[6]. The field has mainly advanced in terms of limiting mechanical injury from ventilation (e.g., with the use of lower tidal volumes[5]), but the benefit on major outcomes of early systematic neuromuscular blockade[48] is being challenged, and some interventions such as prone positioning may be beneficial in most severe forms only[29].<br><br>Numerous trials support the efficacy and safety of volatile anesthetic agent sevoflurane through dedicated devices for the sedation of ICU patients[12,13,50], with superior awakening and extubation times in comparison with current intravenous sedation agents (propofol and benzodiazepines). Because there is current increasing concern over adverse patient consequences associated with our current sedation practice, there is growing interest to find non–benzodiazepine-based alternative sedatives. Notably, sevoflurane may possess important end-organ protective properties mediated via cytoprotective and anti-inflammatory mechanisms that are very relevant to the pathogenesis and resolution of ARDS. Several preclinical studies have shown that inhaled sevoflurane improves gas exchange[7–9], reduces alveolar edema[9] and attenuates pulmonary and systemic inflammation[10,11] in experimental models of ARDS. More recently, a monocenter randomized controlled trial (n=50 patients) found that early use of inhaled sevoflurane in ARDS was associated with improved oxygenation, reduced levels of some pro-inflammatory markers and reduced lung epithelial injury[16], compared to intravenous midazolam. Although this pilot study was not adequately powered to assess major clinical outcomes (day-30 mortality of 36% with sevoflurane vs. 40% with midazolam, P = 0.9), there was a stimulating, yet non-significant, signal towards less ventilator-free days at day 30 with sevoflurane than with midazolam (median, interquartile, 13.0 [1.0-20.0] vs. 5.5 [0.0-28.0], respectively, P = 0.4).<br><br>However, and because no prospective data from multicenter randomized clinical trials are available to date, there remains an important gap in knowledge on the efficacy and safety of a sedation with inhaled sevoflurane in reducing mortality and morbidity in ARDS patients in comparison to current intravenous sedation practice.<br><br>As there is an urgent need for developing novel ARDS therapies to improve survival and decrease its morbidity, an innovative approach based on inhaled sedation with sevoflurane has the potential, in case of positive results, to make a significant breakthrough in the management of patients with ARDS. Given the number of ICU patients with ARDS for whom the question of sedation applies each year worldwide, the study can have significant clinical and public health implications. In addition, and by study design (e.g., enrolment of all patients with moderate-severe ARDS), data from this first multicenter RCT will allow to investigate specific ARDS subphenotypes/endotypes and their specific responses to sevoflurane.

LIST OF ABBREVIATIONS

ACURASYS = The ARDS and Curarisation Systematique study investigators
ARDS = Acute Respiratory Distress Syndrome
BiPAP = Bilevel Positive Airway Pressure
BMI = Body Mass Index
CPAP = Continuous Positive Airway Pressure
CRF = Case Report form
DMSC = Data Monitoring and Safety Committee
ECMO = Extracorporeal Membrane Oxygenation
EDEN = Early vs. Delayed Enteral Nutrition trial
$EtCO_2$ = End-tidal partial pressure of carbon dioxide
FACTT = Fluid and Catheter Treatment Trial
FiO2 = Fraction of Inspired Oxygen
GCS = Glasgow Coma Scale
GRADE = Grading of Recommendations Assessment, Development and Evaluation
ICU = Intensive Care Unit
IL-1β = Interleukin 1β
IL-6 = Interleukin 6
IL-8 = Interleukin 8
IMV = Intermittent Mechanical Ventilation
INR = International Normalized Ratio
ITT = Intent to Treat
KDIGO = kidney disease improving global outcomes
LAR = Legally Authorized Representative
LTAC = Long Term Acute Care Facility
mBW = Measured Body Weight
MRC = Medical Research Council
NHLBI = National Heart Lung and Blood Institute
NIV = Non-Invasive Ventilation
NMBA = Neuromuscular blocking agent
OSCILLATE = Oscillation for Acute Respiratory Distress Syndrome Treatment Early
$PaCO_2$ = Partial pressure of arterial carbon dioxide
PaO_2 = Partial pressure of arterial oxygen
PAP = Pulmonary Artery Pressure
PB = Barometric Pressure
PBW = Predicted Body Weight
PEEP = Positive End-Expiratory Pressure
PETAL = Prevention and Early Treatment of Acute Lung Injury
Pplat = Plateau pressure
PROSEVA = Proning Severe ARDS Patients study investigators
PTSD = Post-Traumatic Stress Disorder
PS = Pressure Support Ventilation
RASS = Richmond Agitation Sedation Scale
RM = recruitment maneuver
SAEs = Adverse events that are serious and unexpected and have a reasonable possibility that the event was due to a study procedure
SBP = Systolic Blood Pressure
SBT = Spontaneous Breathing Trial
SC = Steering Committee
SOFA = Sequential Organ Failure Assessment
SpO_2 = Oxygen Saturation via pulse oximetry
SUSAR = Serious and Unexpected Suspected Adverse Reactions
VFD = Ventilator-free Days
Vt = Tidal volume

TABLE OF CONTENTS

1. General information 13

1.1. Title of the research project 13

1.2. Sponsor code 13

1.3. Sponsor 13

1.4. Study coordination 13

1.5. Study investigators 13

1.5.1. Coordinating investigator 13

1.5.2. Local investigators 14

1.6. Data analysis 18

1.7. Trial monitoring and research coordinators 18

1.8. Steering committee 19

1.9. Scientific committee 20

1.10. Study management committee 20

1.11. Data monitoring and safety committee (DMSC) 20

1.12. Ethics committee 21

1.13. Participating centers 21

1.14. Data monitoring 23

1.15. Provisional study timeline 23

2. Study rationale / Scientific background and hypothesis 23

2.1. Background (current state of scientific knowledge) 23

2.2. Hypothesis 30

2.3. Summary of the benefits and foreseeable and known risks for subjects participating in the research 30

3. Study objectives 33

3.1. Main objective 33

3.2. Secondary objectives 33

4. Study design 34

4.1. Research methodology 34

4.2. Primary outcome measure: ventilator-free days through day 28 34

4.3. Key secondary outcome measure: all location, all-cause 90-day survival 34

4.4. Secondary outcome measures 34

4.4.1. All-location, all-cause 28-day mortality 34

4.4.2. All-cause hospital 28-day mortality 35

4.4.3. All-location, all-cause 14-day mortality 35

4.4.4. All-location, all-cause 7-day mortality 35

4.5. Exploratory outcome measures 35

4.5.1. Ventilator-free days through day 14 (VFD14) 35

4.5.2. Ventilator-free days through day 7 (VFD7) 35
4.5.3. Organ failure-free days through day 7 35
4.5.4. ICU-free days through day 28 35
4.5.5. Hospital-free days through day 28 35
4.5.6. Physiological measures to include: 35
4.5.7. Use of rescue procedures for refractory hypoxemia 36
4.5.8. ICU-acquired delirium 36
4.5.9. Long-term outcome assessments 36
4.5.10. Health economic analysis 36
4.6. Exploratory biologic outcome measures 36
4.7. Safety outcome measures 38
4.8. Subgroups 40
4.9. Measures taken to reduce the risk of bias 41
4.9.1. Randomization 41
4.9.2. Blinding 41
4.9.3. Training and evaluation 41
5. Study population and enrollment 42
5.1. Inclusion criteria 42
5.2. Non-inclusion criteria 43
5.3. Study discontinuation and patient withdrawal 44
5.4. Exclusion period and participation to other research 45
5.5. Financial compensation for participants 45
5.6. Enrollment of study participants 45
6. Study interventions 46
6.1. Detailed description of all acts performed on patients 46
6.2. Diagram of the study 56
6.3. Differences with routine clinical practice 56
6.4. Expected duration of participation for each patient and study schedule 57
7. Products (treatment/medical device/other) used for the study 58
7.1. Description of treatments 58
7.2. Dosage, administration and duration of treatment 58
7.3. Presentation of the study drugs 60
7.3.1. Experimental treatment 60
7.3.2. Non-experimental treatment 64
7.4. Drugs and treatment allowed and not allowed during the study 66
8. Data collection and registration 66
9. Statistics 70
9.1. Sample size estimation 70
Data analysis: generalities 71

9.1.1. Populations 72
9.1.2. Primary analysis 73
9.1.3. Secondary analysis 73
9.2. Method for missing data 75
9.3. Statistical analysis 76
10. Safety assessment – Management of adverse events 76
10.1. Definitions 76
10.2. Serious adverse event reporting 77
10.3. Independent data monitoring and safety committee (DMSC) 79
10.4. Termination of the study 79
10.5. Follow-up of patients presenting an adverse event 80
11. Right of access to source document and data 80
11.1. Access to data 80
11.2. Source data 80
11.3. Data confidentiality 80
11.4. Registration in the national file of biomedical research subjects 81
12. Quality control and assurance 81
12.1. Engagement of the investigators and the sponsor of the study 81
12.2. Quality assurance 81
12.3. Quality control 82
12.4. Case report form 82
13. Ethical considerations 83
13.1. Ethics Committee 83
13.2. Information for patients and written informed consent form 83
13.3. Protocol amendments 83
14. Data processing and storage of study documents 84
14.1. Data entry and processing 84
14.2. CNIL 84
14.3. Data retention and archiving 84
15. Funding and insurance 85
16. Communication - Rules for publication 85
17. Feasibility 85
18. References 88
19. Appendix list 95

1. General information

1.1. Title of the research project

Sevoflurane for Sedation in Acute Respiratory Distress Syndrome: A Multicenter Prospective Randomized Trial (SESAR study)

EudraCT identifying number: 2018-000763-83

ClinicalTrial.gov identifying number: NCT04235608

Version 8 19/04/2021

1.2. Sponsor code

RBHP 2018 JABAUDON

1.3. Sponsor

CHU Clermont-Ferrand
58 rue Montalembert 63003 Clermont-Ferrand cedex 1

Clinical Research Delegation (DRCI)
Tel: +33 (0) 473754963 / Fax: +33 (0) 473754730

1.4. Study coordination

Dr. Matthieu Jabaudon, MD, PhD
Department of Perioperative Medicine
CHU Clermont-Ferrand
Université Clermont Auvergne, CNRS UMR 6293, INSERM U1103, GReD
1, Place Lucie et Raymond Aubrac, 63003 Clermont-Ferrand Cedex 1
Email: mjabaudon@chu-clermontferrand.fr
Tel: +33 (0)4 73 750 501 / Fax: +33 (0)4 73 750 500

1.5. Study investigators

1.5.1. Coordinating investigator

Matthieu JABAUDON, MD, PhD
Department of Perioperative Medicine
CHU Clermont-Ferrand
Université Clermont Auvergne, CNRS UMR 6293, INSERM U1103, GReD
1, Place Lucie et Raymond Aubrac, 63003 Clermont-Ferrand Cedex 1
Email: mjabaudon@chu-clermontferrand.fr
Tel: +33 (0)4 73 750 501 / Fax: +33 (0)4 73 750 500

1.5.2. Local investigators

Dr. Raiko Blondonnet, MD, MSc
Department of Perioperative Medicine
CHU Clermont-Ferrand
Université Clermont Auvergne, CNRS UMR 6293, INSERM U1103, GReD
58, rue Montalembert, 63003 Clermont-Ferrand Cedex 1
Tel: +33 (0)4 73 751 585 / rblondonnet@chu-clermontferrand.fr

Prof. Jean-Michel Constantin, MD, PhD
Dept. of anesthesiology and critical care medicine
Pitié-Salpêtrière Hospital - Assistance Publique-Hôpitaux de Paris (AP-HP)
47-83, bd de l'Hôpital, 75013 Paris, France
Tel: + 33 (0)1 42 162 259 / jean-michel.constantin@psl.aphp.fr

Prof. Antoine Roquilly, MD, PhD
Dept. of anesthesiology and critical care medicine
Hotel Dieu Hospital – University Hospital of Nantes
Nantes, France
Tel: + 33 (0) 240083333 / antoine.roquilly@chu-nantes.fr

Prof. Samir Jaber, MD, PhD
Dept. of anesthesiology and critical care medicine B (DAR B)
Saint-Eloi Hospital – University Hospital of Montpellier
Montpellier, France
Tel: +33 (0) 467337271 / s-jaber@chu-montpellier.fr

Dr. Virginie Lemiale, MD, PhD
Dept. of intensive care medicine
Saint-Louis University Hospital - Assistance Publique-Hôpitaux de Paris (AP-HP)
Paris, France
Tel: +33 (0) 142499421 / virginie.lemiale@aphp.fr

Prof. Carole Ichai, MD, PhD
Dept. of anesthesiology and critical care medicine
Pasteur 2 Hospital - University Hospital of Nice
Nice, France
Tel: +33 (0) 492033627 / ichai@unice.fr

Prof. Lionel Velly, MD, PhD
Dept. of anesthesiology and critical care medicine
Timone Hospital – Assistance Publique-Hôpitaux de Marseille (AP-HM)
Marseille, France
Tel: +33 (0) 413429457 / lionel.velly@ap-hm.fr

Dr. Stéphanie Bulyez, MD
Dept. of anesthesiology, critical care, pain and emergency medicine
Carémeaux Hospital - University Hospital of Nîmes
Nîmes, France
Tel: +33 (0) 466683050 / stephanie.bulyez@chu-nimes.fr

Prof. Sigismond Lasocki, MD, PhD

Dept. of anesthesiology and critical care medicine
University Hospital of Angers
Angers, France
Tel: +33 (0) 241355940 / silasocki@chu-angers.fr

Prof. Jean-Pierre Quenot, MD, PhD
Dept. of intensive care medicine
University Hospital of Dijon
Dijon, France
Tel: +33 (0) 380293751 / jean-pierre.quenot@chu-dijon.fr

Dr. Thomas Lebouvier, MD
Dept. of anesthesiology and critical care medicine
University Hospital of Rennes
Rennes, France
Tel: +33 (0) 299284246 / thomas.lebouvier@chu-rennes.fr

Dr. François Legay, MD
Dept. of intensive care medicine
Hospital of Saint-Brieuc
Saint-Brieuc, France
Tel: +33 (0) 296017060 / francois.legay@ch-stbrieuc.fr

Prof. Arnaud W. Thille, MD, PhD
Dept. of intensive care medicine
University Hospital of Poitiers
Poitiers, France
Tel: +33 (0) 549442167 / aw.thille@gmail.com

Prof. Alexandre Lautrette, MD, PhD
Dept. of anesthesiology and critical care medicine
Jean Perrin Comprehensive Cancer Center
Clermont-Ferrand, France
Tel: + 33 (0) 473278080 / alexandre.lautrette@cjp.fr

Prof. Julien Pottecher, MD, PhD
Dept. of anesthesiology and critical care medicine
Hautepierre Hospital, University Hospitals of Strasbourg
Strasbourg, France
Tel: + 33 (0) 388127095 / julien.pottecher@chru-strasbourg.fr

Dr. Marc Garnier, MD
Dept. of anesthesiology and critical care medicine
Saint-Antoine University Hospital – Assistance Publique-Hôpitaux de Paris (AP-HP)
Paris, France
Tel: +33 (0) 149282362 / marc.garnier@aphp.fr

Dr. Christophe Vinsonneau, MD, MSc
Dept. of intensive care medicine
Hospital of Béthune
Béthune, France
Tel: +33 (0) 321644482 / cvinsonneau@ch-bethune.fr

Dr. Pierre-Marie Bertrand, MD
Cannes, France
Dept. of intensive care medicine
Hospital of Cannes
Cannes, France
Tel: + 33 (0) 493697025 / pm.bertrand@ch-cannes.fr

Dr. Mehran Monchi, MD
Dept. of intensive care medicine
Hospital of Melun-Sénart
Melun, France
Tel: + 33 (0) 181742258 / mn.monchi@gmail.com

Dr. Joël Cousson, MD
Dept. of critical care medicine
University Hospital of Reims
Reims, France
Tel: + 33 (0) 326788067 / jcousson@chu-reims.fr

Prof. Julien Maizel, MD, PhD
Dept. of intensive care medicine
University Hospital of Amiens
Amiens, France
Tel: + 33 (0) 322087840 / maizel.julien@chu-amiens.fr

Prof. Erwan L'Her, MD, PhD
Dept. of intensive care medicine
Cavale Blanche Hospital - University Hospital of Brest
Brest, France
Tel: + 33 (0) 298347181 / erwan.lher@chu-brest.fr

Prof. Belaïd Bouhemad, MD, PhD
Dept. of anesthesiology and critical care medicine
University Hospital of Dijon
Dijon, France
Tel: + 33 (0) 380293079 / belaid_bouhemad@hotmail.com

Prof. Boris Jung, MD, PhD
Dept. of intensive care medicine
Lapeyronie Hospital - University Hospital of Montpellier
Montpellier, France
Tel: + 33 (0) 467337736 / b-jung@chu-montpellier.fr

Prof. Claire Dahyot-Fizelier, MD, PhD
Dept. of anesthesiology and critical care medicine
University Hospital of Poitiers
Poitiers, France
Tel: + 33 (0) 549444587 / Claire.DAHYOT-FIZELIER@chu-poitiers.fr

Dr. Claire Lhommet, MD
Dept. of anesthesiology and critical care medicine
Diaconesses - La Croix Simon Hospital

Paris, France
Tel: + 33 (0) 144641738 / CLhommet@hopital-dcss.org

Dr. Caroline Varillon, MD
Dept. of intensive care medicine
Hospital of Dunkerque
Dunkerque, France
Tel: + 33 (0) 328285613 / caroline.varillon@ch-dunkerque.fr

Dr. Arthur Durand, MD
Dept. of critical care medicine
Salengro Hospital - University Hospital of Lille
Lille, France
Tel: + 33 (0) 320445962 / arthur.durand@chru-lille.fr

Prof. Marc Gainnier, MD, PhD
Dept. of intensive care medicine
Timone Hospital – Assistance Publique-Hôpitaux de Marseille (AP-HM)
Marseille, France
Tel: + 33 (0) 413429637 / marc.gainnier@ap-hm.fr

Dr. Julio Badie, MD
Dept. of intensive care medicine
Hôpital Nord de Franche Comté
Belfort, France
Tel : + 33 (0) 384982191 / Julio.BADIE@hnfc.fr

Dr. Julien Lorber, MD
Dept. of intensive care medicine
Hospital of Saint-Nazaire
Saint-Nazaire, France
Tel : + 33 (0) 272278050 / j.lorber@ch-saintnazaire.fr

Dr. Fabien Lambiotte
Dept. of intensive care medicine
Hospital of Valenciennes
Valenciennes, France
Tel: + 33 (0) 685577221 / lambiotte-f@ch-valenciennes.fr

Dr. Delphine Brégeaud
Dept. of intensive care medicine
Hospital of Saintes
Saintes, France
Tel: + 33 (0) 546951191 / d.bregeaud@ch-saintonge.fr

Dr. Aziz Berrouba, MD
Dept. of critical care medicine
Hospital of Martigues
Martigues, France
Tel: + 33 (0) 442432469 / aziz.berrouba@ch-martigues.fr

Dr. Alexandre Conia, MD
Dept. of intensive care medicine
Hospital of Chartres
Chartres, France
Tel: + 33 (0) 237303030 / aconia@ch-chartres.fr

Dr. Martine Ferrandière, MD
Dept. of anesthesiology and critical care medicine
University Hospital of Tours
Tours, France
Tel: + 33 (0) 247475997 / m.ferrandiere@chu-tours.fr

Dr. Thouy François, MD
Dept. of intensive care medicine
Gabriel Montpied Hospital – University hospital of Clermont-Ferrand
Clermont-Ferrand, France
Tel: + 33 (0) 473754492 / fthouy@chu-clermontferrand.fr

1.6. Data analysis

Bruno PEREIRA, PhD Biostatistics
Biostatistical Unit, Department of Clinical Research and Innovation (DRCI)
CHU Clermont-Ferrand
58, Rue Montalembert, 63003 Clermont-Ferrand Cedex 1
Tel: +33 (0) 473754964 / bpereira@chu-clermontferrand.fr

1.7. Trial monitoring and research coordinators

Dominique Morand
Délégation Recherche Clinique & Innovation (DRCI)
CHU Clermont-Ferrand,
Clermont-Ferrand, France
Tel: +33 (0) 473178409 / dmorand@chu-clermontferrand.fr

Lucile Borao
Department of Perioperative Medicine
CHU Clermont-Ferrand,
Clermont-Ferrand, France
Tel: +33 (0) 473750502 / lborao@chu-clermontferrand.fr

Nathalie Bourguignon
Department of Perioperative Medicine
CHU Clermont-Ferrand,
Clermont-Ferrand, France
Tel: +33 (0) 473750548 / n_bourguignon@chu-clermontferrand.fr

Lise Laclautre Perrier
Délégation Recherche Clinique & Innovation (DRCI)
CHU of Clermont-Ferrand,
Clermont-Ferrand, France

Tel: +33 (0) 473754963 / llacclautre_perrier@chu-clermontferrand.fr

1.8. Steering committee

Dr. Matthieu Jabaudon, MD, PhD
Department of Perioperative Medicine
CHU Clermont-Ferrand
Université Clermont Auvergne, CNRS UMR 6293, INSERM U1103, GReD
1, Place Lucie et Raymond Aubrac, 63003 Clermont-Ferrand Cedex 1
Email: mjabaudon@chu-clermontferrand.fr
Tel: +33 (0)4 73 750 501 / Fax: +33 (0)4 73 750 500

Dr. Raiko Blondonnet, MD, MSc
Department of Perioperative Medicine
CHU Clermont-Ferrand
Université Clermont Auvergne, CNRS UMR 6293, INSERM U1103, GReD
58, rue Montalembert, 63003 Clermont-Ferrand Cedex 1
Email: rblondonnet@chu-clermontferrand.fr
Tel: +33 (0)4 73 751 585 / Fax: +33 (0)4 73 751 614

Pr. Emmanuel Futier, MD, PhD
Department of Perioperative Medicine
CHU Clermont-Ferrand
Université Clermont Auvergne, CNRS UMR 6293, INSERM U1103, GReD
58, rue Montalembert, 63003 Clermont-Ferrand Cedex 1
Email: efutier@chu-clermontferrand.fr
Tel: +33 (0)4 73 751 590 / Fax: +33 (0)4 73 750 500

Pr. Jean-Etienne Bazin, MD, PhD
Department of Perioperative Medicine
CHU Clermont-Ferrand
Université Clermont Auvergne, CNRS UMR 6293, INSERM U1103, GReD
58, rue Montalembert, 63003 Clermont-Ferrand Cedex 1
Email: rblondonnet@chu-clermontferrand.fr
Tel: +33 (0)4 73 751 590 / Fax: +33 (0)4 73 750 500

Prof. Jean-Michel Constantin, MD, PhD
Dept. of anesthesiology and critical care medicine
Pitié-Salpêtrière Hospital, - Assistance Publique-Hôpitaux de Paris (AP-HP)
47-83, bd de l'Hôpital, 75013 Paris, France
Email: jean-michel.constantin@aphp.fr
Tel: + 33 (0)1 42 162 259

Dominique Morand (scientific project manager)
Délégation Recherche Clinique & Innovation (DRCI)
CHU Clermont-Ferrand
58, rue Montalembert, 63003 Clermont-Ferrand Cedex 1
Tel: +33 (0) 473178409 / dmorand@chu-clermontferrand.fr

1.9. Scientific committee

The scientific committee is composed of: **Matthieu Jabaudon** (Department of Perioperative Medicine, CHU Clermont-Ferrand, Université Clermont Auvergne, CNRS UMR 6293, INSERM U1103, GReD), **Raiko Blondonnet** (Department of Perioperative Medicine, CHU Clermont-Ferrand, Université Clermont Auvergne, CNRS UMR 6293, INSERM U1103, GReD), **Jules Audard** (Department of Perioperative Medicine, CHU Clermont-Ferrand, Université Clermont Auvergne, CNRS UMR 6293, INSERM U1103, GReD), **Emmanuel Futier** (Department of Perioperative Medicine, CHU Clermont-Ferrand, Université Clermont Auvergne, CNRS UMR 6293, INSERM U1103, GReD), and **Jean-Etienne Bazin** (Department of Perioperative Medicine, CHU Clermont-Ferrand), **Christophe Quesnel** and **Marc Garnier** (Department of Anesthesiology and Critical Care Medicine, Saint-Antoine University hospital, INSERM UMR 1152, Paris), **Antoine Monsel** and **Jean-Michel Constantin** (Department of Anesthesiology and Critical Care Medicine, Pitié-Salpêtrière University hospital, Paris), **E. Wesley Ely**, **Pratik P. Pandharipande, and Christopher G. Hughes** (Vanderbilt University School of Medicine).

1.10. Study management committee

The study management committee is composed of: **Matthieu Jabaudon** (Department of Perioperative Medicine, CHU Clermont-Ferrand, Université Clermont Auvergne, CNRS UMR 6293, INSERM U1103, GReD), **Raiko Blondonnet** (Department of Perioperative Medicine, CHU Clermont-Ferrand, Université Clermont Auvergne, CNRS UMR 6293, INSERM U1103, GReD), and **Dominique Morand** (Délégation Recherche Clinique & Innovation, CHU Clermont-Ferrand).

1.11. Data monitoring and safety committee (DMSC)

The DMSC will be composed of two clinician-scientists (Prof. Todd W. Rice, Vanderbilt University Medical Center, Nashville, USA and Prof. Laurent Papazian, Assistance-Publique Hôpitaux de Marseille, France) and a biostatistician (Prof. Nicolas Molinari, INSERM, Montpellier, France) that, collectively, have experience in the management of ICU patients, have specific expertise in mechanical ventilation, and in the conduct, monitoring and analysis of randomized clinical trials.

The DMSC will be responsible for safeguarding the interests of trial participants, assessing the safety and efficacy of the interventions during the trial, and for monitoring the overall conduct of the clinical trial. The DMSC will provide recommendations about stopping or continuing the trial to the Steering Committee (SC) of the SESAR trial. To contribute to enhancing the integrity of the trial, the DMSC may also formulate recommendations relating to the selection/recruitment/retention of participants, their management, improving adherence to protocol-specified regimens and retention of participants, and the procedures for data management and quality control.

The DMSC will be empowered to communicate interim findings directly to public health authorities if the committee thinks the additional interim analyses, as planned since **version 6** (Covid-19) of the protocol, produce any results that may be of

relevance to public health with regard to the current Covid-19 outbreak.

1.12. Ethics committee

CPP Ile-de-France II

1.13. Participating centers

In this Covid-19 pandemics situation, the start and conduct of the trial will be managed remotely by the sponsor (CHU Clermont-Ferrand) and the coordinating investigator, including the official study implementation at each participating center, in accordance with the latest guidance of the Agency (ANSM) issued on March 20, 2020 (www.ansm.sante.fr/Activites/Essais-cliniques/Covid-19-Essais-cliniques-en-cours).

- **Estaing and Gabriel Montpied Hospitals, CHU Clermont-Ferrand,** Dept. of Perioperative Medicine
- **Hotel Dieu Hospital, University Hospital of Nantes,** Dept. of Anesthesiology and Critical Care Medicine B
- **Saint Eloi Hospital, University Hospital of Montpellier,** Dept. of Anesthesiology and Critical Care Medicine B (DAR B)
- **Saint-Louis Hospital, Assistance Publique-Hôpitaux de Paris (AP-HP),** Dept. of Intensive Care Medicine
- **Pasteur Hospital, Nice University Hospital,** Dept. of Anesthesiology and Critical Care Medicine
- **Timone hospital, Assistance Publique-Hôpitaux de Marseille (AP-HM),** Dept. of Anesthesiology and Critical Care Medicine
- **Carémeaux Hospital, University Hospital of Nîmes,** Dept. of Anesthesiology, Critical Care, Pain and Emergency Medicine
- **University Hospital of Angers,** Dept. of Anesthesiology and Critical Care Medicine
- **Pitié Salpêtrière University hospital, Assistance Publique-Hôpitaux de Paris (AP-HP),** Dept. of Anesthesiology and Critical Care Medicine
- **University Hospital of Dijon,** Dept. of Intensive Care Medicine
- **University Hospital of Rennes,** Dept. of Anesthesiology and Critical Care Medicine
- **Hospital of Saint-Brieuc,** Dept. of Intensive Care Medicine

- **University Hospital of Poitiers,** Dept. of Intensive Care Medicine
- **Jean Perrin Comprehensive Cancer Center,** Dept. of Anesthesiology and Critical Care Medicine
- **Hautepierre Hospital, University Hospitals of Strasbourg,** Dept. of Anesthesiology and Critical Care Medicine
- **Saint-Antoine Hospital, Assistance Publique-Hôpitaux de Paris (AP-HP),** Dept. of Anesthesiology and Critical Care Medicine
- **Hospital of Béthune**, Dept. of Intensive Care Medicine
- **Hospital of Cannes,** Dept. of Intensive Care Medicine
- **Hospital of Melun-Sénart,** Dept. of Intensive Care Medicine
- **University Hospital of Reims,** Dept. of Critical Care Medicine
- **University Hospital of Amiens,** Dept. of Intensive Care Medicine
- **Cavale Blanche Hospital, University Hospital of Brest,** Dept. of Intensive Care Medicine
- **University Hospital of Dijon,** Dept. of Anesthesiology and Critical Care Medicine
- **Lapeyronie Hospital, University Hospital of Montpellier,** Dept. of Intensive Care Medicine
- **University Hospital of Poitiers,** Dept. of Anesthesiology and Critical Care Medicine
- **Diaconesses - La Croix Saint Simon Hospital,** Dept. of Anesthesiology and Critical Care Medicine
- **Hospital of Dunkerque,** Dept. of Intensive Care Medicine
- **Salengro Hospital, University Hospital of Lille,** Dept. of Critical Care Medicine
- **Timone Hospital, Assistance Publique-Hôpitaux de Marseille (AP-HM),** Dept. of Intensive Care Medicine
- **Hôpital Nord de Franche Comté,** Dept. of Intensive Care Medicine
- **Hospital of Saint-Nazaire,** Dept. of Intensive Care Medicine
- **Hospital of Valenciennes,** Dept. of Intensive Care Medicine

➢ **Hospital of Saintes,** Dept. of Intensive Care Medicine

➢ **Hospital of Martigues,** Dept. of Intensive Care Medicine

➢ **Hospital of Chartres,** Dept. of Intensive Care Medicine

➢ **University Hospital of Tours,** Dept. of Anesthesiology and Critical Care Medicine

➢ **Gabriel Montpied Hospital – University Hospital of Clermont-Ferrand,** Dept. of Intensive Care Medicine

1.14. Data monitoring

Clinical Research Delegation (DRCI), CHU Clermont-Ferrand, Clermont-Ferrand, France

1.15. Provisional study timeline

- Submission to Ethics committee (CPP): September 2018
- Approval by Ethics committee (CPP): December 2018
- Regulatory authorization (Agence National de Sécurité du Médicament et des Produits de Santé-ANSM): December 2018
- Start of the study: May 2020
- Inclusion period: 3 years
- Planned study completion: May 2023
- End of study report: October 2024*

*The DMSC will be empowered to communicate interim findings directly to public health authorities if the committee thinks the additional interim reports, as planned since version 6 (Covid-19) version of the protocol, produce any results that may be of relevance to public health with regard to the current Covid-19 outbreak.

2. Study rationale / Scientific background and hypothesis

(See the accompanying “NOTICE - SESAR trial v5 (Covid-19)” document for the specific rationale of investigating inhaled sedation with sevoflurane in Covid-19-related ARDS)

2.1. Background (current state of scientific knowledge)

Acute respiratory distress syndrome (ARDS) is defined using the clinical criteria of bilateral pulmonary opacities on chest radiograph, arterial hypoxemia [partial pressure of arterial oxygen (PaO_2) to fraction of inspired oxygen (FiO_2) ratio ≤300 mmHg with positive end-expiratory pressure (PEEP) ≥5 cmH_2O] within one week of a clinical insult or new or worsening respiratory symptoms, and the exclusion of cardiac

failure as the primary cause[1,2]. ARDS is a deadly condition for intensive care unit (ICU) patients, with mortality rates of 35-45%, and a frequently under-recognized challenge for clinicians [2]. ARDS represents more than 10% of ICU admissions and nearly 25% of ICU patients requiring mechanical ventilation, with a hospital mortality rate of 35-45%[2]. Beyond such a high mortality rate, ARDS is associated with greater healthcare utilization, reduced quality of life among survivors and worse long-term physical and cognitive outcomes[39–46]. Fifty years after its first clinical description[47], and despite intense research, the identification of an effective ARDS therapy has failed[6]. The field has mainly advanced in terms of limiting mechanical injury from ventilation (e.g., with the use of lower tidal volumes[5]), but the benefit on major outcomes of early systematic neuromuscular blockade[48] has recently been challenged, and some interventions such as prone positioning may be beneficial in most severe forms only[29].

Volatile agents have been used for more than 150 years to provide general anesthesia[51]. Expansion of their role as sedatives with potentially other therapeutic properties for critical care patients has gained increasing interest over the last 30 years. Current sedation practice predominantly relies on benzodiazepines (midazolam, lorazepam, and diazepam), propofol, and ketamine, which are commonly combined with opioids to provide analgesia and cosedation[52]. The sedative and hypnotic properties of benzodiazepines and propofol are mediated by promoting central type-A ɣ-aminobutyric acid (GABAa) receptor activity, although propofol has wider effects on glycine, nicotinic, and muscarinic receptors[52,53]. Ketamine possesses hypnotic and analgesic effects by directly blocking N-methyl-D-aspartate receptors and hyperpolarization-activated cyclic nucleotide channels but also has wider action on cholinergic, opioid, and aminergic systems[54]. Benzodiazepines are widely available, inexpensive, and familiar to critical care health professionals. However, there is growing concern surrounding the consequences of oversedation from high doses of these agents with slow metabolism and clearance, which can impact awakening times, duration of mechanical ventilation, hemodynamic stability, and perhaps even mortality[52,55,56]. Prolonged and heavy use of benzodiazepines may also promote drug tolerance, withdrawal, delirium, and long-term neuropsychiatric disorders (depression, anxiety, and post-traumatic stress disorders)[52,57–59]. Propofol may induce propofol infusion syndrome and is associated with greater cost, hemodynamic instability, and hypertriglyceridemia during prolonged use in comparison to benzodiazepines[52,60].

Greater awareness of these effects has led to suggestions to use alternative nonbenzodiazepine strategies (Grade +2B) within the revised PAD (pain, agitation, delirium) guidelines published in 2013[52]. Dexmedetomidine is a newer agent that provides analgesia with "lighter" sedation promoting greater patient interaction. Limitations of dexmedetomidine include high cost, common adverse effects of bradycardia and hypotension, inability to potentially provide deeper sedation as a single agent when clinically indicated, and limited license of use[61,62].

Modern-day volatile agents consist of sevoflurane, desflurane, and isoflurane. These small fluorinated hydrocarbons possess subtle structural differences that impact their physicochemical properties, onset speed, potency, dosing, metabolism, and clearance[63]. Their mechanisms of action have been previously described elsewhere[64]; briefly, modern theory of volatile activity involves complex interaction with multiple proteins on the pre- and postsynaptic nerve membrane as well as non-neural tissue. Volatiles reduce presynaptic excitation and neurotransmitter release through inhibition of sodium (Na^+) and several isoforms of calcium (Ca^{2+}) voltage-gated

channels and promote repolarization through activation of potassium (K^+) channels. Volatiles reduce neurotransmitter activity in the postsynaptic membrane by enhancing inhibitory ion channel activity mediated by GABAa and glycine receptors as well as inhibiting excitatory ion channels mediated by nicotinic acetylcholine, serotonin type 3 (5HT3), glutamate (glut), N-methyl-D-aspartate, and ɑ-amino-3-hydroxy-5-methyl-4-isoazolepropionic acid receptors. Volatiles are also likely to possess widespread effects on G-protein–coupled receptors and intracellular signaling pathways on nerve and other cell types[12]. Volatiles have a rapid onset of action, with no significant concerns of drug tolerance or tachyphylaxis[65]. Rapid offset is aided by drug clearance via simple pulmonary exhalation with low levels of hepatic metabolism (sevoflurane 5%, isoflurane 0.2%, desflurane 0.02%) and production of no significant active metabolites[63]. This contrasts with benzodiazepines, propofol, and dexmedetomidine, which rely on adequate hepatic and renal synthetic function for metabolism and clearance. Systemic accumulation of these intravenous agents, particularly among elderly and ICU patients who often display hepatic and renal dysfunction, leads to reduced clearance and "drug hangover" that can slow patient awakening and extubation[52]. Desflurane undergoes the least biotransformation and displays the fastest onset/offset, followed by sevoflurane and isoflurane. However, desflurane is not commonly used in the ICU, given its higher cost and need for specialist equipment, because its boiling point is close to room temperature. Despite the faster onset and elimination times, it is important to note that, like intravenous agents, volatile anesthetics are capable of causing deep sedation levels that can lead to respiratory depression and reduced patient activity during the sedation period. Thus, whatever the type of drug, even if it has faster onset and elimination times, all recommended guidelines for sedation-analgesia management should be used along with fast-acting agents[52]. This includes the use of validated sedation and pain scales, prescription of a sedation target, implementation of bedside nurse-driven sedation algorithms, as well as checking safety criteria for daily awakening test to avoid inappropriate deep or prolonged sedation. Volatiles are available in liquid formulations that require vaporization before inhalation. Sedation for ICU patients is often achievable at doses approximately one-third of those required for general anesthesia (0.2–0.3 minimum alveolar concentration), although higher doses may be required, particularly in those patients requiring deeper sedation levels when clinically indicated[66]. Their administration involves routine bedside gas monitoring, which provides capnography and unique ability to accurately monitor breath-by-breath volatile concentrations delivered to and exhaled by the patient. The expired end-tidal concentration provides an excellent real-time method to monitor the cerebral concentration, which aids dose titration and minimizes risk of drug overdosing.

Volatiles have been reserved in the ICU to manage medically intractable status asthmaticus, status epilepticus, and complex sedation scenarios in patients with high sedation requirements, such as burns, chronic pain, multiple surgeries, and history of drug abuse[65–68]. It is recognized that this class of agents has powerful dose-dependent hypnotic, bronchodilator, and anticonvulsant properties. Wider ICU uptake has been limited due to technical challenges of needing large anesthesia machines, scavenging systems to minimize atmospheric pollution, and limited familiarity with this class of drugs among intensivists. Over the past 20 years, the availability of specialized ventilators and miniature vaporizers, such as the Anesthesia Conserving Device (AnaConDa; Sedana Medical, Danderyd, Sweden) or the more recently investigated MIRUS® system (Pall Medical, Dreieich, Germany), have simplified bedside volatile administration (**Figure 1**)[12,66]. AnaConDa is more commonly available and is placed between the endotracheal tube and Y-piece of the ventilator circuit. Sevoflurane or

isoflurane is infused into the device for vaporization before inhalation. Desflurane cannot be used with this device, given this agent's low boiling point. AnaConDa has a built-in carbon layer that allows for more than 80% recycling of the expired agent, which facilitates low infusion rates of 1 to 5 mL/h of volatile agent[66]. As recommended by the manufacturer, this device is replaced every 24 hours. The AnaConDa must be used with a separate bedside gas analyzer and gas scavenging system. Currently, the MIRUS device is available in Europe, and AnaConDa is available in 20 countries predominantly located in Europe, Canada, and Australia (excluding United States). The addition of the AnaConDa or MIRUS® device to the breathing circuit will increase dead space by approximately 100 mL. Very recently, a miniaturized version of the AnaConDa (namely AnaConDa-S, Sedana Medical, Danderyd, Sweden) has been recently developed, thus decreasing the device dead space to approximately 50 mL, and allowing a minimal tidal volume of 200 mL (vs. 350 mL with the AnaConDa). Recent work from our group demonstrated an increased work of breathing when the AnaConDa device is placed in the breathing circuit without volatile sedation in adult patients with no history of chronic pulmonary disease[19]. However, these altered respiratory parameters were normalized when low doses of sevoflurane were used with the AnaConDa device, which may be partially due to the bronchodilator effects of these agents and reduction in respiratory drive. Ventilator weaning of patients with low doses of volatile agents is feasible, but removal of the AnaConDa device from the breathing circuit when no sedation is required may be advisable to improve patient comfort and respiratory parameters.

Because of historical data linking high atmospheric volatile levels with infertility and spontaneous abortions, gas scavenging of expired volatiles has become routine in the operating room to ensure occupational atmospheric levels are maintained below recommended national safety standards of less than 2 parts per million in North America and less than 50 parts per million in the United Kingdom[69]. Similarly, atmospheric pollution is minimized by combining standard room air exchanges with capturing expired waste gases using passive or active scavenging systems in conjunction with AnaConDa to ensure workplace safety. Passive gas adsorption uses charcoal canisters (Contrafluran, Novasorb) attached to the ventilator expiratory port[66]. Excellent safety profiles have been demonstrated using active scavenging systems, which siphon waste gases to the main central hospital waste gas outlet system or use suction-assisted adsorption systems[70–72]. Atmospheric pollution can be further reduced by connecting the gas analyzer's output using a Y-connector to the passive charcoal adsorber or active system. Room air recycling varies among institutions, between the operating room and critical care unit, and thus it would be advisable to check air recycling within the ICU rooms and also monitor volatile atmospheric levels using infrared spectrophotometric monitors or dosimeters[70,72]. To minimize drug spillage and inhalation of volatile agents, filling of the AnaConDa syringe should be performed by trained personnel.

Cost analyses of the use of volatiles for ICU sedation have been performed by several European centers where the AnaConDa has been more commonly used and retails for 70 to 80€. In a series of 15 patients who received isoflurane for an average of 4 days, the cost of midazolam/sufentanil sedation (171 ± 101€) was comparable to isoflurane/sufentanil (122 ± 44€), which was inclusive of drug, device, and scavenging costs[73]. A short-term postoperative sedation randomized controlled trial comparing desflurane to propofol sedation showed overall drug costs for volatiles was lower (95€ desflurane vs. 171€ propofol) and cost neutral with the addition of the AnaConDa device[74]. Other centers using sevoflurane for short-term sedation have shown that

volatile sedation is more expensive than intravenous propofol sedation[75]. Currently, we lack a cost-effectiveness analysis that takes into account any beneficial clinical outcomes such as faster awakening, extubation times, and lengths of ICU stay.

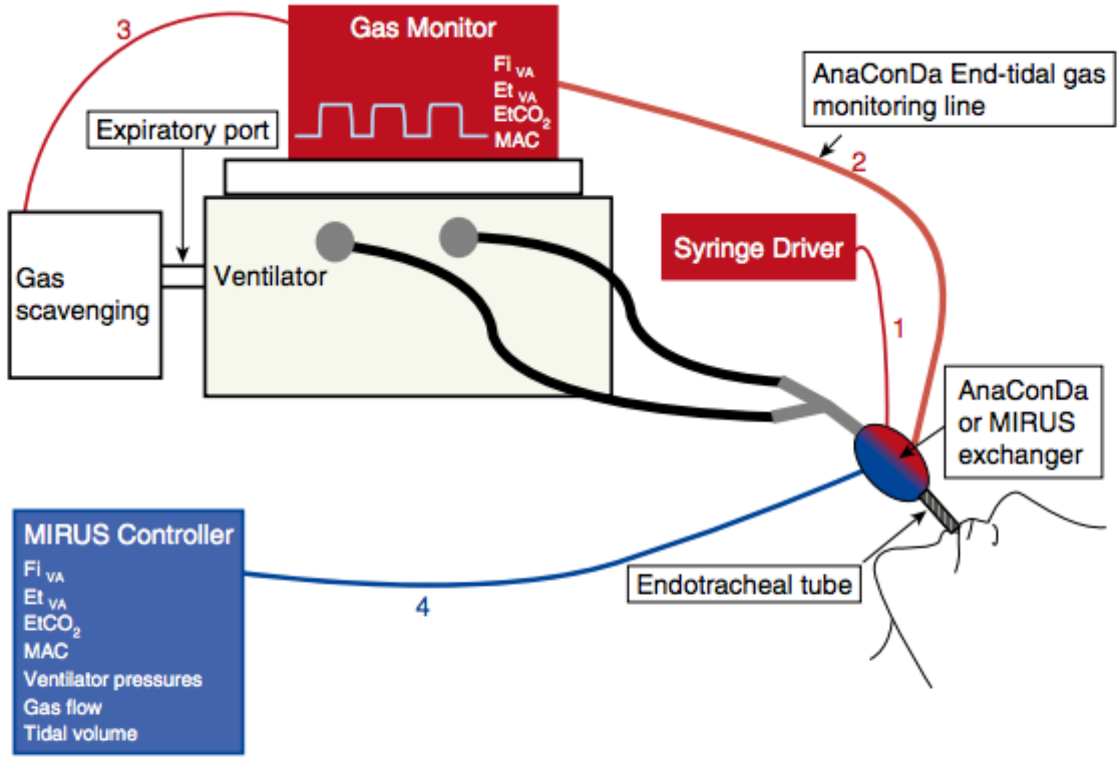

Figure 1. Bedside equipment setup for administering volatile agents. AnaConDa or MIRUS exchanger is placed between the endotracheal tube and Y-piece of the ventilator breathing circuit. These devices contain a reflector, which recycles expired volatile agent and humidifier/antibacterial filter. Use of these devices does not require any additional humidification and increases dead space of the breathing circuit by approximately 100 ml. Expired volatile agent is scavenged at the ventilator expiratory port. Equipment setup for AnaConDa is marked in *red* and MIRUS system in *blue*.[12]

Volatile halogenated anesthetics such as sevoflurane and desflurane are widely used to provide general anesthesia in the operating room. Worldwide, more than 230 million patients undergoing major surgery each year require general anesthesia and mechanical ventilation[76], and postoperative pulmonary complications adversely affect clinical outcomes and healthcare utilization[77]. The use of sevoflurane was associated with reduced lung inflammation in patients undergoing thoracic surgery, compared to propofol, and with significant reductions in composite adverse events including ARDS, pneumonia, atelectasis, pleural effusion, and bronchopleural fistula[78]. Although further studies are warranted to address the impact of volatile anesthetics on outcome in noncardiac surgery, a similar reduction in pulmonary complications has been demonstrated in a recent meta-analysis showing that volatile as opposed to intravenous anesthesia is associated with a significant reduction in overall mortality for cardiac surgical patients[79].

Volatile anesthetics have been shown in preclinical models to both prevent and minimize the extent of inflammatory lung injury[12,15]. The most commonly proposed mechanism for these effects in various clinical studies and animal models of lung injury, including inhaled endotoxin, ventilator-induced lung injury (VILI), sepsis, and hemorrhagic shock[8,78] is through a reduction in pro-inflammatory cytokine release. Fortis and colleagues recently reported that sevoflurane suppressed pulmonary inflammation in a two-hit experimental model of lung injury (acid instillation and VILI)

exerting a lung-protective effect, which is likely mediated by pulmonary GABAa receptors[80]. Incubation of human airway epithelial cells with sevoflurane after anoxia reduces the mRNA expression of interleukin 6 (IL-6), IL-8, and monocyte chemoattractant protein 1a through an inhibition in the nuclear translocation of nuclear factor kappa beta (NFKb)[81]. In lipopolysaccharide (LPS)-induced ARDS, sevoflurane is associated with decreased pulmonary release of inflammatory mediators (tumor necrosis factor (TNF)-α, cytokine-induced neutrophil chemoattractant protein-1 (CINC-1), macrophage-inflammatory protein-2 (MIP-2) and monocyte chemoattractant protein-1 (MCP-1)) and increased expression of anti-inflammatory protein phosphorylated extracellular-regulated kinase (pErk)[11,82]. Additional work using a rat model of ARDS showed that preconditioning with isoflurane prevents increases in inflammatory mediators found in bronchoalveolar lavage (BAL) samples and reduces histological evidence of lung injury via upregulation of reactive oxygen species in the pre-exposure period[83]. Arguably the mechanism of this effect could stem from a systemic reduction in inflammation also seen with intravenous anesthetics. However, Ferrando's work in a porcine model of ARDS showing that pre-exposure to sevoflurane results in reduced levels of BAL inflammatory markers and neutrophil cells compared to propofol suggests a local effect rather than a systemic one[7]. Of note, macrophage-mediated neutrophil migration seems to be attenuated by sevoflurane either in a preconditioning or postconditioning setting[10,11]. Recently, a novel pathway of lung protection for volatile anesthetics has been suggested. In a murine model mimicking a patient with ARDS who is then exposed to VILI, Englert's group found that administration of volatile anesthetics during the mechanical ventilation phase reduced the degree of physiologic lung dysfunction not through reductions of inflammatory mediators but rather through preservation of alveolar-epithelial integrity[84]. A schematic representation summarizing the proposed mechanisms of lung protection for volatile anesthetics is found in **Figure 2**.

Taken together, preclinical studies have shown that inhaled sevoflurane improves gas exchange[7–9], reduces alveolar edema[9] and attenuates pulmonary and systemic inflammation[10,11] in experimental models of ARDS. Interestingly, these effects point out major features that contribute to mortality in ARDS, namely impaired lung epithelial function and integrity[4], and subphenotypes of severe inflammation[21].

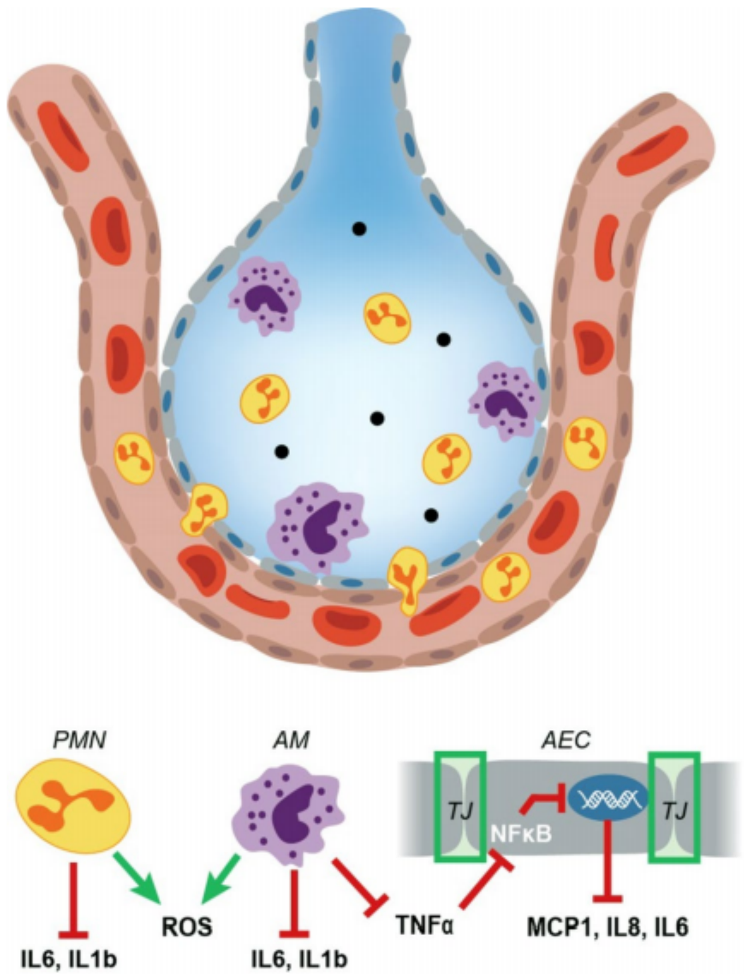

Figure 2. Proposed mechanisms of lung protection for the volatile anesthetics. Depicted is a representation of the alveolar capillary interface with the relevant cell types and mediators involved in lung injury. Red arrows indicate processes inhibited by volatile anesthetics, green arrows indicate processes enhanced by volatile anesthetics. The green boxes represent preservation of tight junctions. PMN: polymorphonuclear cell; A: alveolar macrophage; AEC: alveolar epithelial cell; IL6: interleukin 6; IL1b: interleukin 1 beta; ROS: reactive oxygen species; TNFa: tumor necrosis factor alpha; TJ: tight junction; NFKb: nuclear factor kappa b; MCP1: monocyte chemoattractant protein 1; IL8: interleukin 8.[15]

Specific prospective data regarding the use of inhaled sedation in the ICU to prevent or treat lung injury is lacking[12,15]. However, a retrospective analysis of patients receiving inhaled sedation suggests an association between its use and reductions in 1-year and in-hospital mortality, perhaps related to a significant increase in ventilator-free days compared to sedation with intravenous agents[85]. Numerous trials support the efficacy and safety of inhaled sevoflurane for the sedation of ICU patients[50,86], and sevoflurane is associated with shorter wake-up and extubation times[13,14]. As the use of volatile agents gains popularity in the ICU setting, evidence suggesting that sevoflurane may also protect against inflammatory lung injury may provide insight into the potential additional benefit these agents can offer for the lung-injured patient.

A recent monocenter randomized controlled trial (n=50 patients) from our group found that early use of inhaled sevoflurane in ARDS (through the AnaConDa device) was associated with improved oxygenation, reduced levels of some pro-inflammatory markers and reduced lung epithelial injury[16], compared to intravenous midazolam. In this study, as in others[13,14,18,19], sevoflurane inhalation through dedicated device AnaConDa was well tolerated, with no major adverse effect, e.g. on renal function or

respiratory mechanics. Although this first pilot study was not adequately powered to assess major clinical outcomes (day-30 mortality of 36% with sevoflurane versus 40% with midazolam, P = 0.9), there was a stimulating, yet non-significant, signal towards less ventilator-free days at day 30 with sevoflurane than with midazolam (median, interquartile, 13.0 [1.0-20.0] versus 5.5 [0.0-28.0], respectively, P = 0.4).

However, and because no prospective data from multicenter randomized clinical trials are available to date, there remains an important gap in knowledge on the efficacy and safety of sedation with inhaled sevoflurane in reducing mortality and morbidity in ARDS patients, compared to current intravenous sedation practice. Thus, the benefits and risks of such a strategy of inhaled sedation with sevoflurane in patients with ARDS require further evaluation.

2.2. Hypothesis

We hypothesized that a strategy of inhaled sedation with sevoflurane could be more effective than current intravenous sedation practice in improving a composite outcome of mortality and time off the ventilator at 28 days in patients with ARDS.

2.3. Summary of the benefits and foreseeable and known risks for subjects participating in the research

See the accompanying “NOTICE - SESAR trial v5 (Covid-19)” document for the summary of the potential and specific benefits during the Covid-19 pandemics.

The findings from a large international observational study recently emphasized that ARDS is still a frequent and deadly condition. ARDS represents more than 10% of ICU admissions and nearly 25% of ICU patients requiring mechanical ventilation, with a hospital mortality rate of 35-45%[2]. Beyond such a high mortality rate, ARDS is associated with greater healthcare utilization, reduced quality of life among survivors and worse long-term physical and cognitive outcomes[39–46]. Although the field has mainly advanced in terms of limiting mechanical injury from ventilation (e.g., with the use of lower tidal volumes[5]), the identification of an effective ARDS therapy has failed[6] and few therapies or interventions have proven beneficial in ARDS.

Available evidence from previous preclinical and clinical studies indicate that:

- Inhaled sedation with sevoflurane is associated with shorter awakening and extubation times in ICU patients in comparison with current intravenous sedation agents (propofol and benzodiazepines)[13,14]

- Inhaled sevoflurane improves gas exchange[7–9], reduces alveolar edema[9] and attenuates pulmonary and systemic inflammation[10,11] in experimental models of ARDS

- Inhaled sevoflurane was associated with improved oxygenation, reduced levels of some pro-inflammatory markers and reduced lung epithelial injury in patients with ARDS, compared to intravenous midazolam, in a first monocenter randomized controlled trial from our group[16]

The results from this study can have significant clinical and important public health implications, especially:

- Reduced morbidity and mortality
- Reduced ICU and hospital lengths of stay
- Reduced healthcare utilization

There are also limitations and controversies of using volatile agents within the ICU.

First, volatile agents are well known triggers for patients genetically predisposed to malignant hyperthermia. This condition is hallmarked by sudden-onset hemodynamic instability, hypercarbia, hyperthermia, muscle rigidity, and extremely high serum creatine kinase. Suspicion of this syndrome requires early intervention with immediate change of the ventilator circuit, dantrolene infusion, artificial cooling with specialist follow up, and genetic and muscle biopsy testing. A case of malignant hyperthermia has been identified during sevoflurane therapy in a patient with pneumonia[87]. However, this condition remains rare (1/50,000–100,000) in comparison to propofol infusion syndrome, which affects up to 1% of ICU patients[60].

Second, some types of patients may be unsuitable for inhalational sedation secondary to equipment limitations. The ideal weight-based tidal volume with the AnaConDa is unknown, but a minimum tidal volume of 350 mL for pediatric patients is recommended to overcome device dead space and avoid rebreathing of carbon dioxide. This may not be feasible in smaller patients who require lung-protective (6 mL/kg predicted body weight (PBW)) or even ultra-protective (<4 mL/kg PBW) ventilation protocols and those who need one-lung ventilation strategies. This device may also become impractical in patients with high-volume bronchial secretions, which may occlude and prevent optimal drug delivery. Hopefully, a miniaturized version of the AnaConDa (AnaConDa-S, Sedana Medical, Danderyd, Sweden) has been recently developed, thus decreasing the device dead space to approximately 50 mL and allowing a minimum tidal volume of 200 mL. Of note, our recent experience of inhaled sevoflurane using the AnaConDa in ICU patients with ARDS supports its feasibility and safety, in particular with regards to its respiratory and hemodynamic effects[16]. For example, arterial pH, $PaCO_2$, expired tidal volume, respiratory rate, FiO_2, PEEP level, inspiratory plateau pressure, static pulmonary compliance, airway resistance, doses of infused norepinephrine, mean arterial pressure and heart rate did not significantly differ between ARDS patients receiving inhaled sevoflurane and those receiving intravenous midazolam[16].

Third, the true impact of volatiles on delirium has not been directly studied using the currently recommended measurement tools of Confusion Assessment Method for the ICU or the Intensive Care Delirium Screening Checklist[88]. Several sedation trials have performed other cognitive and psychological assessment by measuring the level of postextubation agitation and applying 14-point ICU Memory tool, which assesses delusion, negative feelings, and factual ICU memories[13,89,90]. These studies compared isoflurane, sevoflurane, and desflurane to either midazolam or propofol and

demonstrated a predominantly non–statistically significant trend in the reduction of these events in patients who received volatile sedation. Investigation of the development of long-term neuroaffective disorders was assessed by Sackey and colleagues in a trial of 40 patients who received isoflurane or midazolam sedation[90]. Anxiety and depression were assessed using the well-validated Hospital Anxiety and Depression Scale, and post-traumatic stress disorder was assessed using the Impact of Event Scale at 6 months post ICU discharge. This study showed no difference in the psychological morbidity between these two groups.

Fourth, fluoride ions are constituents of all volatile agents. Methoxyflurane is an older-generation volatile agent, no longer in use, that shows high lipid solubility and undergoes 50 to 70% biotransformation to form fluoride. Historical work conducted by Cousins and Mazze during the 1970s identified that fluoride levels beyond 50 µmol/L can impair the renal tubular concentrating ability leading to high-output renal failure[91]. This safety threshold has continued to be used despite modern-day anesthetic drugs displaying markedly different pharmacokinetic profiles. The elimination half-life of serum fluoride ions is 21.4 to 24.8 hours. Sevoflurane, isoflurane, and desflurane have 7, 5, and 6 fluoride ions, respectively, but undergo low levels of metabolism to produce inorganic fluoride. Serum fluoride levels do rise during both short anesthetic and longer ICU duration of use, with sevoflurane displaying higher levels than isoflurane given its greater metabolism and fluoride content[67,92]. However, no significant association between renal dysfunction and fluoride levels has been identified despite several patients displaying levels beyond 50 µmol/L[92]. Taken together, no significant effect of inhaled sevoflurane on renal function has been observed to date in clinical studies of ICU patients.

As there is an urgent need for developing novel ARDS therapies to improve survival and decrease its morbidity, an innovative approach based on inhaled sedation with sevoflurane has the potential, in case of positive results, to make a significant breakthrough in the management of patients with ARDS. Given the number of ICU patients with ARDS for whom the question of sedation applies each year worldwide, the study can have significant clinical and public health implications. In addition, and by study design, data from this first multicenter RCT will be highly valuable to further investigate specific ARDS subphenotypes (as defined by various clinical, radiological and/or biological measures), and their specific response to sevoflurane (ARDS endotypes). On the other hand, and based on previous clinical studies[13,16,18,19,71,75,92], there should be only limited serious adverse events (SAEs) for patients in this research. In addition, the study protocol stresses that inhaled sevoflurane should be interrupted in case of significant sevoflurane-induced changes in hemodynamic or respiratory parameters.

3. Study objectives

3.1. Main objective

To assess the efficacy of a sedation with inhaled sevoflurane in improving a composite outcome of mortality and time off the ventilator at 28 days in patients with moderate-severe ARDS in comparison to a control group receiving intravenous sedation with propofol.

3.2. Secondary objectives

- To evaluate the safety (clinical adverse events) of the two sedation strategies
- To describe the effects of inhaled sevoflurane, compared to intravenous sedation, on respiratory mechanics, gas exchange, and physiologic measures
- To describe the effects of inhaled sevoflurane, compared to intravenous sedation, on duration of mechanical ventilation
- To describe the effects of inhaled sevoflurane, compared to intravenous sedation, on organ dysfunction
- To describe the effects of inhaled sevoflurane, compared to intravenous sedation, on the use of rescue procedures (e.g., nitric oxide, epoprostenol sodium, high frequency ventilation, extracorporeal membrane oxygenation (ECMO), and neuromuscular blockade use after 48h from randomization
- To describe the effects of inhaled sevoflurane, compared to intravenous sedation, on ICU-acquired delirium
- To describe the effects of inhaled sevoflurane, compared to intravenous sedation, on long-term outcomes (e.g., disability, health-related quality of life, self-rated health, pain-interference, post-traumatic stress-like symptoms, cognitive function, subsequent return to work and healthcare use)
- To assess between-group healthcare-related costs during ICU stay and hospital stay
- To assemble a biological collection of plasma, alveolar, and urine samples for future mechanistic studies of the effects of sevoflurane:
    - o on plasma levels of biological markers of a hyperinflammatory ARDS phenotype (IL-8, TNF-receptor 1, bicarbonates)[22], of ventilator-induced lung injury (IL-6)[5], of lung endothelial injury (angiopoietin (ANG)-2)[93,94], and of epithelial injury and impaired AFC (sRAGE)[17,95]
    - o on urine levels of biological markers of acute kidney injury (tissue inhibitor of metalloproteinase 2 (TIMP-2) and insulin-like growth factor binding protein7 (IGFBP-7)[96])
    - o on plasma levels of total fluoride ions and hexafluoroisopropanol
- To assess the presence of subphenotypes among patients with ARDS, based on distinct clinical, imaging[25,27,28], and/or biological[21,22] profiles (endotypes), and their differential therapeutic response to sevoflurane, if any
- To assemble, in a subset of patients from both groups enrolled in selected centers (because of logistical considerations), a biological collection of undiluted pulmonary edema fluid for future studies of the biological effects of sevoflurane
- To assemble, in a subset of patients from both groups enrolled in selected centers (because of logistical considerations), a biological collection of heat moisture exchanger and AnaConDa-S filters in patients enrolled in the control and intervention groups, respectively, for future studies of biomarker

measurement in the filter fluid as representative of biomarker measurement in the distal airspace fluid in ARDS patients receiving inhaled sedation[97]

- To assess the specific effects of inhaled sevoflurane in patients with Covid-19-related ARDS

4. Study design

4.1. Research methodology

Investigator-initiated multicenter, prospective, randomized, stratified, parallel-group clinical trial with blinded outcome assessment and concealed allocation of patients with moderate-to-severe ARDS to a strategy of inhaled sedation with sevoflurane or to a strategy of intravenous sedation with propofol.

4.2. Primary outcome measure: ventilator-free days through day 28

In this study, the primary outcome is the number of days alive and off the ventilator at 28 days (VFD28, for ventilator-free days to day 28), thereby considering death as a competing event.

Ventilator-free days through day 28 are defined as the number of days from the time of initiating unassisted breathing to day 28 after randomization, assuming survival for at least two consecutive calendar days after initiating unassisted breathing and continued unassisted breathing to day 28. If a patient returns to assisted breathing and subsequently achieves unassisted breathing to day 28, VFDs will be counted from the end of the last period of assisted breathing to day 28. A period of assisted breathing lasting less than 24 hours and for the purpose of a surgical procedure will not count against the VFD calculation. If a patient was receiving assisted breathing at day 27 or dies prior to day 28, VFDs will be zero. Patients transferred to another hospital or other health care facility will be followed to day 28 to assess this endpoint.

4.3. Key secondary outcome measure: all location, all-cause 90-day survival

All location, all-cause 90-day survival will be assessed on study day 91.

4.4. Secondary outcome measures

4.4.1. All-location, all-cause 28-day mortality

All location, all-cause 28-day mortality will be assessed on study day 29.

4.4.2. All-cause hospital 28-day mortality

All location hospital 28-day mortality will be assessed on study day 29.

4.4.3. All-location, all-cause 14-day mortality

All location, all-cause 14-day mortality will be assessed on study day 15.

4.4.4. All-location, all-cause 7-day mortality

All location, all-cause 7-day mortality will be assessed on study day 8.

4.5. Exploratory outcome measures

4.5.1. Ventilator-free days through day 14 (VFD14)

The number of days alive and off the ventilator at 14 days, thereby considering death as a competing event.

4.5.2. Ventilator-free days through day 7 (VFD7)

The number of days alive and off the ventilator at 7 days, thereby considering death as a competing event.

4.5.3. Organ failure-free days through day 7

Organ failure is defined as present on any date when the most abnormal vital signs or clinically available lab value meets the definition of clinically significant organ failure according to SOFA scores (Appendix G). Patients will be followed for development of organ failures to death, hospital discharge or study day 7, whichever comes first. Each day a patient is alive and free of a given organ failure will be scored as a failure-free day. Any day that a patient is alive and free of all organ failures will represent days alive and free of all organ failure.

4.5.4. ICU-free days through day 28

4.5.5. Hospital-free days through day 28

4.5.6. Physiological measures to include:

a. Change in Oxygenation Index from day 1 to day 7 (defined as a continuous time-dependent variable)
b. Change in PaO_2/FiO_2 ratio from day 1 to day 7 (defined as a continuous time-dependent variable)
c. Change in $PaCO_2$ and arterial pH from day 1 to day 7 (defined as a continuous time-dependent variable)
d. Change in the level of PEEP from day 1 to day 7 (defined as continuous time-dependent variables)

e. Change in plateau pressure, and static compliance of the respiratory system from day 1 to day 7 (defined as continuous time-dependent variables)
f. Change in ventilatory ratio, as defined as [minute ventilation (mL/min) * arterial PCO_2 (mmHg)]/[predicted body weight * 100 * 37.5][99], from day 1 to day 7 (defined as a continuous time-dependent variable)

4.5.7. Use of rescue procedures for refractory hypoxemia

Rescue procedures will be chosen according to the practice at the clinical site. We will record the use of the following rescue procedures (yes/no) through study day 28: nitric oxide, epoprostenol sodium, high frequency ventilation, ECMO, and neuromuscular blockade use after 48h from randomization.

4.5.8. ICU-acquired delirium

The Confusion Assessment Method for the ICU (CAM-ICU)[103] will be assessed daily from study entry to study day 7, death or ICU discharge, whichever comes first.

4.5.9. Long-term outcome assessments

We will assess seven measures after hospitalization:

a. **Disability**: using Katz Activities of Daily Living (ADL)[106]
b. **Health-Related Quality of Life** (including utilities): Short Form-36 (SF-36)
c. **Self-rated health**: 1 standard item
d. **Pain-interference**: 1 standard item
e. **Post-traumatic Stress-like Symptoms**: Post-Traumatic Stress Symptoms (PTSS-14)[107,108], Hospital Anxiety and Depression Scale[109,110]
f. Cognitive function: the Alzheimer's Disease 8 (AD8)[111]
g. Subsequent return to work, hospital and ED use, and location of residence

These measures will be collected through mail surveys sent to patients or their legally authorized representatives (LARs). Surveys will be performed by basic research staff using detailed scripts appropriate for each survey instrument. Manuals of operations will be developed for training, reference and quality assurance review.

4.5.10. Health economic analysis

Healthcare-related costs during ICU stay and hospital stay will be assessed in both groups in collaboration with Mrs. Charline Mourgues, hospital engineer and health economist at CHU Clermont-Ferrand.

4.6. Exploratory biologic outcome measures

Plasma and urine samples will be collected from indwelling catheters (when available) at study entry and on days 1, 2, 4, 6 and 14 or ICU discharge (whichever

occurs first) in order to assemble a biological collection aimed at further investigating the effects of inhaled sedation with sevoflurane in patients with ARDS.

We will measure, among others, plasma biological markers of a hyperinflammatory ARDS phenotype (IL-8, TNF-receptor 1, bicarbonates)[22], of VILI (IL-6)[5,49], of lung endothelial injury (ANG-2)[93,94], and of epithelial injury and impaired AFC (sRAGE)[17,95]. We will also assess urine levels of biological markers of acute kidney injury (tissue inhibitor of metalloproteinase 2 (TIMP-2) and insulin-like growth factor binding protein 7 (IGFBP-7)[96]). In addition, we will also assess plasma total fluoride and hexafluoroisopropanol levels in order to further investigate the production of sevoflurane metabolites, and their relationships with renal function, in ICU patients under inhaled sedation with sevoflurane.

We will also collect whole blood samples at study entry and on day 2 for future RNA and DNA studies.

The main objectives of these future biological analyses are:

- to assess the effects of sevoflurane on major pathways of lung injury and repair (such as inflammation and epithelial or endothelial injury),
- to assess the presence of subphenotypes among patients with ARDS enrolled in the SESAR trial, based on distinct clinical, imaging[25,27,28], and/or biological[21,22] profiles (endotypes), and their differential therapeutic response to sevoflurane, if any,
- to verify the safety of sevoflurane on renal function,
- to identify therapy response traits to inhaled sevoflurane in patients with ARDS.

In particular, transcriptomics studies will be performed to investigate how inhaled sevoflurane can affect the expression of genes involved in mechanisms of lung injury and repair. Genomics studies will be used to investigate whether single-nucleotide polymorphisms (within genes relevant to mechanisms of lung injury and repair) could identify patients at highest risk of worse outcome and/or patients who are more likely to benefit from inhaled sevoflurane.

Undiluted pulmonary edema fluid samples will be collected at study entry and 24 hours later in a total of 100 patients (n=50 patients from each group) enrolled in selected centers (because of logistical considerations).

In addition, heat moisture exchanger and AnaConDa-S filters will be collected at 24 hours in those patients randomized to the control and intervention groups (n=30 patients from each group), respectively. These samples will be used for future analyses:

- of the biological effects of sevoflurane on lung injury and repair
- of biomarker measurement in the filter fluid as representative of biomarker measurement in the distal airspace fluid in ARDS patients receiving inhaled sedation[97]

Additional alveolar fluid samples (in intubated patients, through bronchoalveolar lavage) within 48 hours from study entry and between day 4 and day 6, in a total of 25 patients enrolled in 5 selected centers (because of logistical considerations; expected number of patients per center=5). These samples will be prepared for protein biomarker studies.

It is anticipated that the biological collection may not be done as initially planned (i.e., without alveolar samples at least), or even not done at all, during

the Covid-19 pandemics, in order to minimize the risk for intensive care providers to be contaminated with the virus and to ensure no additional work overload is needed from intensive care providers.

Because the current Covid-19 outbreak and ICU surge may be at high risk of staff shortage with extreme material and organizational difficulties for healthcare providers to take care of all patients admitted to the ICU, priority will be given, whenever possible, to the collection of plasma samples because those samples are very likely those that will more easily better inform mechanistic investigations on the effects of inhaled sevoflurane on inflammation and acute lung injury.

Importantly, neither alveolar fluid (undiluted edema fluid or through bronchoalveolar lavage) nor HME/AnaConDa sampling will be mandatory during the Covid-19 pandemics and, when performed, sampling will aim at minimizing the virus spreading and protect all intensive care providers, as currently recommended (Recommandations d'experts SRLF-SFAR-SFMU-GFRUP-SPILF sur la prise en charge en réanimation des patients en période d'épidémie à SARS-CoV2).

4.7. Safety outcome measures

- Change in hemodynamic measures (mean arterial pressure, dose of infused norepinephrine or other vasopressor, serum lactate level) from day 1 to day 7 (defined as continuous time-dependent variables)
- Changes in KDIGO criteria for acute kidney injury[26] from day 1 to day 7 (defined as continuous time-dependent variables) (Appendix H)
- Supraventricular tachycardia (SVT) or new onset atrial fibrillation (the occurrence of one or more episodes during the ICU stay will be recorded)
- Severe hypercapnic acidosis with pH <7.15, in the absence of metabolic acidosis and despite further tidal volume and/or respiratory rate increase, as described in the protocol (AnaConDa-S device)
- Development of malignant hyperthermia through day 7 (sevoflurane and propofol)
- Development of propofol-related infusion syndrome through day 7 (propofol)
- Development of pneumothorax or bronchopleural fistula persistent despite drainage, through day 7

SESAR trial outcome measures

Primary outcome	Ventilator-free days through day 28 (VFD28), as defined as the number of days alive and off the ventilator at 28 days, thereby considering death as a competing event*
Key secondary outcome	All-location, all-cause 90-day survival (assessed on study day 91)
Secondary outcomes	• All-location, all-cause 28-day mortality (assessed on study day 29)<br>• All-cause hospital 28-day mortality (assessed on study day 29)<br>• All-location, all-cause 14-day mortality (assessed on study day 15)<br>• All-location, all-cause 7-day mortality (assessed on study day 8)
Exploratory outcomes	• Ventilator-free days through day 14 (VFD14)<br>• Ventilator-free days through day 7 (VFD7)<br>• Organ failure-free days through day 7**<br>• ICU-free days through day 28<br>• Hospital-free days through day 28<br>• Physiological measures to include:<br>o Changes in oxygenation index, PaO_2/FiO_2, $PaCO_2$, and arterial pH from day 1 to day 7 (defined as continuous time-dependent variables)<br>o Changes in the level of PEEP, plateau pressure and compliance of the respiratory system, and in ventilatory ratio# from day 1 to day 7 (defined as a continuous time-dependent variable)<br>• Use of rescue procedures for refractory hypoxemia through day 28: nitric oxide, epoprostenol sodium, high frequency ventilation, ECMO, and neuromuscular blockade use after 48h from randomization.<br>• ICU-acquired delirium: CAM-ICU assessed daily from study entry to study day 7, death or ICU discharge, whichever comes first.<br>• Long-term outcome assessments at 3 and 12 months:<br>o Disability: Katz Activities of Daily Living<br>o Health-Related Quality of Life: Short Form-36<br>o Self-rated health: 1 standard item<br>o Pain-interference: 1 standard item<br>o Post-traumatic Stress-like Symptoms: Post-Traumatic Stress Symptoms-14, Hospital Anxiety and Depression Scale<br>o Cognitive function: Alzheimer's Disease 8<br>o Subsequent return to work, hospital and ED use, and location of residence<br>• Healthcare-related costs during ICU stay and hospital stay
Exploratory biologic outcomes	• Change in plasma biomarkers of IL-8, sTNFr1, bicarbonates (hyperinflammatory ARDS phenotype), IL-6 (VILI), ANG-2 (endothelial activation), and sRAGE (alveolar epithelial injury) (defined as continuous time-dependent variables)§<br>• Change in urine biomarkers of TIMP-2 and IGFBP-7 (acute kidney injury)§<br>• Change in plasma total fluoride and hexafluoroisopropanol (sevoflurane metabolism)<br>• Genetic analysis: DNA and RNA at baseline and 48 h<br>• Change in total protein within undiluted pulmonary edema fluid at baseline and 24 h (alveolar fluid clearance)£<br>• Composition of fluid from HME filter (control group) and AnaConDa-S device (intervention group) at baseline and 24 h$<br>• Composition of BAL fluid within 48 h from study entry and between day 4 and day 6€
Safety outcomes	• Changes in hemodynamic measures (mean arterial pressure, dose of infused norepinephrine or other vasopressor, serum lactate level) and in KDIGO criteria for acute kidney injury from day 1 to day 7 (defined as continuous time-dependent variables)<br>• Supraventricular tachycardia or new onset atrial fibrillation during the ICU stay

	• Severe hypercapnic acidosis with pH <7.15[%] (AnaConDa-S device)<br>• Development of malignant hyperthermia through day 7 (sevoflurane and propofol)<br>• Development of propofol-related infusion syndrome through day 7 (propofol)<br>• Development of pneumothorax or bronchopleural fistula persistent despite drainage, through day 7

Definition of abbreviations: SESAR = sevoflurane for sedation in acute respiratory distress syndrome; VFD = ventilator-free days; ICU = intensive care unit; PEEP = positive end-expiratory pressure; ECMO = extracorporeal membrane oxygenation; CAM-ICU = confusion assessment method for the ICU; ED = emergency department; IL-8 = interleukin 8; sTNFR1 = soluble tumor necrosis factor receptor 1; IL-6 = interleukin 6; ANG-2 = angiopoietin 2; sRAGE = soluble receptor for advanced glycation end-products; TIMP-2 = tissue inhibitor of metalloproteinase 2; IGFBP-7 = insulin-like growth factor binding protein 7; DNA = deoxyribonucleic acid; RNA = ribonucleic acid; HME = heat moisture exchanger; BAL = bronchoalveolar lavage; KDIGO = kidney disease improving global outcomes.

**Ventilator-free days through day 28 are defined as the number of days from the time of initiating unassisted breathing to day 28 after randomization, assuming survival for at least two consecutive calendar days after initiating unassisted breathing and continued unassisted breathing to day 28. If a patient returns to assisted breathing and subsequently achieves unassisted breathing to day 28, VFDs will be counted from the end of the last period of assisted breathing to day 28. A period of assisted breathing lasting less than 24 hours and for the purpose of a surgical procedure will not count against the VFD calculation. If a patient was receiving assisted breathing at day 27 or dies prior to day 28, VFDs will be zero. Patients transferred to another hospital or other health care facility will be followed to day 28 to assess this endpoint.*

***Organ failure is defined as present on any date when the most abnormal vital signs or clinically available lab value meets the definition of clinically significant organ failure according to SOFA scores. Patients will be followed for development of organ failures to death, hospital discharge or study day 7, whichever comes first. Each day a patient is alive and free of a given organ failure will be scored as a failure-free day. Any day that a patient is alive and free of all organ failures will represent days alive and free of all organ failure.*

[#]*Ventilatory ratio = [minute ventilation (mL/min)*$PaCO_2$ (mmHg)]/[predicted body weight*100*37.5]*

[§]*Plasma and urine samples will be collected from indwelling catheters (when available) at study entry and on days 1, 2, 4, 6 and 14 or ICU discharge (whichever occurs first).*

[£]*In 50 patients from each group.*

[$]*In 30 patients from each group.*

[€]*In a total of 25 patients.*

[%]*In the absence of metabolic acidosis and despite further tidal volume and/or respiratory rate increase, as described in the protocol.*

4.8. Subgroups

A priori subgroups will include:

- Pre-randomization PaO_2/FiO_2 <100 mmHg
- Pre-randomization presence vs. absence of shock (defined as intravenous infusion of vasoactive drugs)
- Time from meeting ARDS severity criteria for study enrollment to start of sedation with inhaled sevoflurane
- Patients with hypoinflammatory vs hyperinflammatory subphenotypes[21,22,112,113]
- Patients with higher vs. lower degrees of lung epithelial injury or of impaired AFC (as assessed by baseline plasma sRAGE)
- Patients with higher vs. lower degrees of lung endothelial injury (as assessed by baseline plasma ANG-2)
- Patients with focal vs. non focal loss of aeration as evaluated by chest radiographs, when available
- Pre-randomization presence vs. absence of suspected or Covid-19 infection

4.9. Measures taken to reduce the risk of bias

4.9.1. Randomization

Enrolled patients will be randomized by local investigators using a dedicated, password-protected, SSL-encrypted website (CSOnline, Clinsight) accessible 24-hour around-the-clock to allow immediate and concealed allocation.

Each patient will be given a unique patient-number and a randomization number. Randomization sequence will be generated by minimization, and will be stratified by study center, by the degree of ARDS severity (PaO_2/FiO_2 <100 mmHg), by the suspicion or presence of Covid-19 as a cause for ARDS, and by the presence of shock (defined as intravenous infusion of vasoactive drugs) or not at randomization.

4.9.2. Blinding

It will not be possible to mask the assigned sedation strategy from the treating clinicians because they have an ethical responsibility to ensure patient safety during the emergency procedures. However, procedures will be put in place to minimize the possibility of bias arising because research staff becomes aware of trial group allocation. At each participating center, patients will be followed up for primary and secondary endpoints by members of the research staff who will be unaware of the trial group allocation. Information on whether the primary and secondary outcomes occur will be collected and entered into the electronic web-based case report form (eCRF) by trial or clinical trained personal (clinical research associate), blinded to the allocation group, under the supervision of the local principal investigator (PI) or designee who will also be unaware of the trial group allocation.

Moreover, in addition to the aforementioned risk of allocation/selection bias (controlled by randomization) and performance bias, the study protocol will control for the risks of attrition bias (all analyses will be performed on data from the intention-to-treat population), whereas the use of well-defined and pre-specified primary/secondary outcome measures will control for the risk of reporting bias. Finally, the independent trial statistician and the members of the data monitoring and safety committee (DMSC) will also remain blinded for the allocation during analysis. However, the observation of differences in serious adverse events between the two groups (see sections 10.2 and 10.3) will allow, for safety reasons may the DMSC deem necessary, to unblind allocation groups.

4.9.3. Training and evaluation

Although all critical care professionals who will participate in the study and deliver inhaled sedation to ARDS patients are certified and allowed to manage ICU sedation, their previous use and expertize level of inhaled sedation with sevoflurane may largely vary due to limited dissemination of this technique among ICUs to date.

Therefore, an educational program will be conducted prior to patient recruitment to ensure that all participating centers have sufficient training to ensure patient safety and reach study goals.

First, online-based theoretical presentations (on inhaled sedation, sevoflurane, and the AnaConDa-S) and practical training (text and video tutorials, clinical scenarios, proposed protocol for inhaled sedation with sevoflurane; see Appendix N) will be provided to local investigators and a group of super-users (e.g., among nurses, research nurses, and research associates) at each site. This mandatory training will be evaluated using an online-based evaluation module.

Then, on-site training sessions will be organized to ensure that all aspects of inhaled ICU sedation have been mastered and that all potential issues related to inhaled sedation with sevoflurane in ARDS patients have been extensively addressed before patient recruitment starts. In addition, data and safety monitoring will be more frequently performed for the first patients enrolled in each center, in order to ensure strict adherence to the study protocol and intended use of inhaled sedation in patients with ARDS.

Finally, methods to assess whether the study results might be associated with some degree of "learning effect" (due to the specific training on inhaled sedation) will be used, e.g. through sensitivity analyses (described in the Statistics section).

Because the *in situ* training of all centers, as planned initially, will not be feasible in practice due to the recent self-confinement measures related to the current context of widespread disease resulting from the Covid-19 pandemics, the trial will first start in centers with previous experience in the clinical use of inhaled sedation in critically ill patients (N=14). Other centers will be trained as described above and allowed to participate only when the pandemic situation will allow it.
In this Covid-19 pandemics situation, the start and conduct of the trial will be managed remotely by the sponsor (CHU Clermont-Ferrand) and the coordinating investigator, including the official study implementation at each participating center, in accordance with the latest guidance of the Agency (ANSM) issued on March 20, 2020 (www.ansm.sante.fr/Activites/Essais-cliniques/Covid-19-Essais-cliniques-en-cours).

5. Study population and enrollment

5.1. Inclusion criteria

1. Age ≥18 years
2. Presence for ≤24 hours of all of the following conditions, within one week of a clinical insult or new or worsening respiratory symptoms:
    a. PaO_2/FiO_2 <150 mmHg with positive end-expiratory pressure (PEEP) ≥8 cmH_2O[i,ii,iii]
    or, if arterial blood gas not available, SpO_2/FiO_2 ratio that is equivalent to a PaO_2/FiO_2 <150 mmHg with PEEP ≥8 cmH_2O (Appendix A1), and a confirmatory SpO_2/FiO_2 ratio between 1-6 hours after the initial SpO_2/FiO_2 ratio determination[iii,iv]
    b. Bilateral opacities not fully explained by effusions, lobar/lung collapse, or nodules
    c. Respiratory failure not fully explained by cardiac failure or fluid overload; need objective assessment (e.g., echocardiography) to exclude hydrostatic edema if no risk factor present

i. If altitude >1,000 m, then PaO_2/FiO_2 <150 x (PB/760).
ii. These inclusion criteria ensure a non-transient, established hypoxia that persists despite elevated PEEP and time. Initial, post-intubation, PEEP is typically <8 cmH_2O.
iii. The qualifying PaO_2/FiO_2 or the SpO_2/FiO_2 must be from intubated patients receiving at least 8 cmH_2O PEEP.
iv. When hypoxia is documented using pulse oximetry, a confirmatory SpO_2/FiO_2 ratio is required to further establish persistent hypoxia. Qualifying SpO_2/FiO_2 must use SpO_2 values less than or equal to 96% Qualifying SpO_2 must be measured at least 10 minutes after any change to FiO_2.

The 24-hour enrollment time window begins when criteria a-c are met. Criteria may be

met at either the Network or referring hospital.
The first qualifying SpO_2/FiO_2 (not the confirmatory SpO_2/FiO_2) is used to determine this time window.

5.2. Non-inclusion criteria

- Absence of affiliation to the French *Sécurité Sociale*
- Patient under a tutelage measure or placed under judicial protection
- Continuous sedation with inhaled sevoflurane at enrollment
- Known pregnancy
- Currently receiving ECMO therapy
- Chronic respiratory failure defined as $PaCO_2$ >60 mmHg in the outpatient setting
- Home mechanical ventilation (non-invasive ventilation or via tracheotomy) except for CPAP/BIPAP used solely for sleep-disordered breathing
- Body mass index >40 kg/m^2
- Chronic liver disease defined as a Child-Pugh score of 12-15 (Appendix A2)
- Expected duration of mechanical ventilation <48 hours
- Moribund patient, i.e. not expected to survive 24 hours despite intensive care
- Burns >70% total body surface
- Previous hypersensitivity or anaphylactic reaction to sevoflurane or cisatracurium
- Medical history of malignant hyperthermia
- Long QT syndrome at risk of arrhythmic events
- Medical history of liver disease attributed to previous exposure to a halogenated agent (including sevoflurane)
- Known hypersensitivity to propofol or any of its components
- Known allergy to eggs, egg products, soybeans, and soy products
- Suspected or proven intracranial hypertension
- Tidal volume of 6 mL/kg predicted body weight (PBW) below 200 mL (as recommended by the manufacturer for the use of the AnaConDa-S (Sedana Medical, Danderyd, Sweden)
- Enrollment in another interventional ARDS trial with direct impact on sedation and mechanical ventilation
- Endotracheal ventilation for greater than 120 hours (5 days)
- Persistent bronchopleural fistula despite chest tube drainage
- PaO_2/FiO_2 (if available) >200 mmHg after meeting inclusion criteria and before randomization

As oxygenation may improve during the 24-hour enrollment window, this exclusion criterion ensures that patients with mild ARDS are not included in the study.

Pregnancy testing (based on urine measurement of human chorionic gonadotropin (hCG), or on its serum measurement in anuric patients) will be systematically performed to rule out pregnancy in female patients of reproductive age, as per current practice in all participating centers.

Because patients with ARDS are very likely to receive deep sedation and invasive mechanical ventilation (or to need emergent tracheal intubation and invasive mechanical ventilation if not already the case), they are very likely to lack capacity to provide informed consent when eligible to enrolment into the study and the study protocol provides for a waiver of informed consent from the patient. In addition, because in emergency situations, sedation and ventilation must be initiated as early

as possible, the study protocol implies a short enrollment time window of 24 hours since ARDS Berlin criteria are met. Therefore, the consent from the patient's next of kin will be sought actively. In case the patient's next of kin cannot be reached during this short time window, the investigator will decide to include the patient in the study using an emergent consent procedure; no consent from the patient's next of kin will be required in this very specific case, but the investigator will inform the patient's next-of kin of his/her decision to include the patient in the study whenever possible.

Deferred informed consent will be obtained as soon as possible from participants for potential continuation of the research.

In the specific context of the Covid-19 pandemics, because of the isolation measures in place to limit the spread virus and of the ICU staff work overload, it is very likely that the patient's next of kin or family will not be easily reachable, even by phone. Therefore, it is anticipated that the number of patients enrolled using the emergent consent procedure could be much higher than usual. However, local investigators will always be committed to obtain deferred informed consent from the patient or its next of kin when, and as soon as, possible.

Patients <18 years old are excluded because of limited clinical trial data with sevoflurane in these individuals. In addition, we will only be enrolling patients from adult ICUs, and the staff may be less well-trained in sedation and neuromuscular blockade practices in children. Patients with ARDS for >24 hours or on mechanical ventilation for >120 hours are excluded because our study aims at testing early treatment. Criteria 3 excludes patients who are already receiving inhaled sevoflurane as part of their clinical care. Exclusion criterion 4 is included because there are not sufficient data to support the use of sevoflurane in pregnant women during treatment for severe ARDS. Criteria 5-12 exclude patients who may not survive to important study endpoints or whose underlying condition or ventilator management complicates assessment of the secondary endpoint of ventilator free days. Patients with large burns (criterion 12) are also excluded as conservative fluid management may be contraindicated.

5.3. Study discontinuation and patient withdrawal

No formal criteria will be set for stopping the study. Nevertheless, a participant who no longer agrees to participate in the clinical trial may withdraw its consent at any time without need of further explanation. Patients who are withdrawn from the trial protocol will be followed up and analyzed as with the remaining patients. In order to conduct intention-to-treat analyses with as little missing data as possible, it is in the interest of the trial to collect as much data from each participant as possible. Therefore, the investigator may ask the participant which aspects of the trial he/she wishes to withdraw from (participation in the remaining follow-up assessments or use of already collected data) and, whenever possible, the participant will be asked for permission to obtain data for the primary outcome measure. If this person declines, all data from that patient will be destroyed and a new patient will be randomized to obtain the full sample size. All randomized patients will be reported, and all data available with consent will be used in the analyses. If appropriate, missing data will be handled in accordance with multiple imputation procedures if missing data are greater than 5 %.

The study will be overseen by a steering committee and a data monitoring and safety committee (DMSC). The steering committee will be jointly responsible with the independent DMSC for safeguarding the interests of the participating patients.

Recommendations for pausing or stopping the study will be made by the DMSC in case of safety reasons (group-difference is found in suspected unexpected serious adverse reactions or serious adverse events). The steering committee will be responsible to continue, hold or stop the study based on the DMSC recommendations.

In case of early discontinuation of the study, the sponsor will inform the competent authorities (ANSM, CPP) within 15 days of the date on which the decision is made.

5.4. Exclusion period and participation to other research

There will be no exclusion period.

Except for interventional ARDS trials with direct impact on sedation and mechanical ventilation, the participation to other research will be allowed, after approval by the principal investigator, only if there is no influence on the primary outcome measure during the entire study period (28 days).

5.5. Financial compensation for participants

There will be no financial compensation for participants.

5.6. Enrollment of study participants

Patients are expected to be included from 37 centers during a 3-year period. Each of the 37 centers has to include 1 patient per month (holidays excluded) to finish inclusion within 3 years. High incidence of ARDS in the ICU setting (10% of ICU admissions, and nearly 25% of ICU patients requiring mechanical ventilation, in a large recent observational study[2]) and the available data from each participating study center indicate that the study is highly feasible.

Patients will be screened from the emergency departments, ICUs and other acute care areas of the participating centers. The overall strategy is to screen and enroll early, every newly intubated, acutely ill or postoperative, patient at each site, using clinically obtained pulse oximetry and blood gases.

Tactics will include:

i. Follow up each screened patient on a daily basis.

Epidemiological studies have demonstrated that 90% of at-risk patients will develop ARDS within 5 days. ARDS Network trials have successfully used this "*screen and follow*" strategy in facilitating early enrollment of eligible patients.

ii. ICU screening.

Every new ICU admission receiving mechanical ventilation will be screened. This will include but not be limited to admissions from the ED, wards, and operating room. We will also assess patients transferred from outside hospitals. The enrollment window for these patients will include the time elapsed since admission at the outside hospital including during transfer.

iii. Study clinician availability for consent.

Each site will have dedicated study physicians and coordinators who are certified and trained in human subjects protection and understand the study protocol.

After checking the eligibility and ineligibility criteria, study inclusion will be performed.

Because, in acute emergent situations, sedation must be initiated as early as possible, and because of a short (24-hour) time window for enrollment, the study protocol provides for a waiver of informed consent from the patient. In this case, the consent from the patient's next of kin will be sought actively. In case the patient's next of kin cannot be reached during this short time window, the investigator will decide to include the patient in the study using an emergent consent procedure and inclusion will be validated by both a local investigator and an independent physician from outside the ICU. The investigator will inform the patient's next-of kin of his/her decision to include the patient in the study whenever possible. Deferred informed consent will be obtained as soon as possible from participants for potential continuation of the research.

6. Study interventions

Trials should be conducted in a setting reflective of best practice that can be clearly described and reproduced in a clinical non-trial setting. We therefore (i) selected centers already providing high-quality standardized ICU care, (ii) will document the use of protocols and order sets at each center, (iii) monitor the provision and results of key processes of care, and (iv) implement strict protocols (with training, monitoring, and feedback) for the use of inhaled sevoflurane and mechanical ventilation. All participating centers have existing protocols and order sets for routine sedation management, glucose control, septic shock resuscitation, deep venous thrombosis prophylaxis, and other aspects of background care.

All patients must be randomized within 24 hours of meeting inclusion criteria. The window for randomization begins at the time of meeting all inclusion criteria, regardless of patient location. After randomization, the low tidal volume protocol must be initiated within two hours (if not already being used). In both arms, deep sedation followed by neuromuscular blockade must be initiated within four hours of randomization.

6.1. Detailed description of all acts performed on patients

Multicenter prospective, randomized, stratified, parallel-group clinical trial with concealed allocation to:

- Inhaled sedation with sevoflurane, as vaporized via the miniaturized Anesthesia Conserving Device (AnaConDa-S, Sedana Medical, Danderyd, Sweden). AnaConDa-S will be placed between the endotracheal tube and Y-piece of the ventilator breathing circuit (**Figure 1**), and sevoflurane will be infused into the device for vaporization before inhalation. Inspired sevoflurane fraction ($F_I$sevo), $F_E$sevo and expired CO_2 ($EtCO_2$) will be continuously monitored using a separate bedside gas analyzer. AnaConDa-S has a built-in carbon layer that allows for more than 80% recycling of the expired agent;

residual expired gas will be scavenged following manufacturer's instructions using an active carbon filter[66]; filling of the AnaConDa syringe will be performed by trained personnel using dedicated materials, and closed tracheal suctioning systems may be used to further prevent atmospheric pollution and exposure of healthcare workers to sevoflurane. Therefore, an additional active humidifier filter should not be used in association with the AnaConDa-S. As recommended by the manufacturer, AnaConDa will be replaced every 24 hours.

- Intravenous sedation with propofol, as already routinely used in participating ICUs.

In each group, patients will receive the allocated sedation strategy from randomization until sedation can be definitely interrupted or until day 7, whichever occurs first. If sedation needs to be applied again within 7 days after randomization, the sedative agent to use will be based on the randomization arm (inhaled sevoflurane vs. intravenous propofol). After day 7, decisions on further use of sedative agents, including type of and dosing of the agent, will be as per the treating clinicians.

All interventions, except for the sedation choice (either with intravenous propofol (considered as current standard practice) or with inhaled sevoflurane (study intervention)) are based on current guidelines on the management of severe ARDS in general and of patients with Covid-19 in particular (Surviving Sepsis Campaign: Guidelines on the Management of Critically Ill Adults with Coronavirus Disease 2019; The Australian and New Zealand Intensive Care Society (ANZICS) COVID-19 Guidelines; Recommandations d'experts SRLF-SFAR-SFMU-GFRUP-SPILF sur la prise en charge en réanimation des patients en période d'épidémie à SARS-CoV2).

In addition, details have been added to the interventions in both study arms, especially with regards to viral isolation and filtration methods, which are of paramount importance in the management of Covid-19 patients:

- The virus filtration capacity of the AnaConDa is >99.9% for 27 nm particles, less than one quarter of the size of the SARS-CoV-2 virus (120-160 nm), implying very low risk for SARS-CoV-2 virus passing the AnaConDa (**Appendix A of the accompanying "NOTICE - SESAR trial v5 (Covid-19)" document**). **The AnaConDa device is therefore safe to use in patients with Covid-19**.
- As currently recommended (Recommandations d'experts SRLF-SFAR-SFMU-GFRUP-SPILF sur la prise en charge en réanimation des patients en période d'épidémie à SARS-CoV2), **an additional "high-efficiency particulate air" (HEPA) filter will be connected to the expiratory port of the breathing circuit, to protect the ventilator for exhaled virus entry in all patients with suspected or confirmed Covid-19 (Appendix B of the accompanying "NOTICE - SESAR trial v5 (Covid-19)" document**).
  - In patients randomized to receive inhaled sedation through the AnaConDa device, it is therefore possible to return the waste gas from the gas monitor in the expiratory side of the breathing circuit, before the additional virus filter, by using the standard bypass connector (normally placed at the FlurAbsorb filter) (**Appendix C of the accompanying "NOTICE - SESAR trial v5 (Covid-19)" document**).
  - Exhaled air is sampled to the anesthetic gas monitor from the patient side of the filter and is thus not filtered. However, most gas monitors that will be used in SESAR have viral filters in the water trap, with different

degrees of virus filtering capacity (**Appendix D of the accompanying "NOTICE - SESAR trial v5 (Covid-19)"document**).

- **In patients from the control group (intravenous sedation with propofol), a heat and moisture exchanger (HME)-HEPA filter will be used and connected between the endotracheal tube and the Y-piece of the respiratory circuit**, as initially planned in the previous version of the protocol.

Study staff will ensure a Richmond Agitation-Sedation Scale (RASS) of -4 to -5 (Ramsay of 5-6, or Riker of 1-2) before starting, and during, the cisatracurium besylate infusion in both arms. Initiation of neuromuscular blockade, if not already being used, must begin within 4 hours of randomization. Patients will receive a cisatracurium besylate bolus of 15 mg, followed by a continuous infusion of 37.5 mg/hour for a maximum of 48 hours[48,49]. We chose this fixed, relatively high dosage for simplicity (train-of-four titration imperfect and with limited evidence base[114–116]) and to help ensure effective neuromuscular blockade (clinical observation and train-of-four monitoring can lead to under-dosing). This dosage is the same as used in the ACURASYS trial[48]. We chose cisatracurium as its metabolism is independent of hepatic and renal function. In the rare circumstance that neuromuscular blockade is deemed inadequate, (i) check the patient and the ventilator to confirm the correct reading, (ii) check the infusion rate and drug to confirm correct, (iii) rebolus, using the below recommendation.
Recommendation: If the end-inspiratory plateau pressure remains greater than 30 cmH_2O for at least 10 minutes, it is recommended that the patient receive the administration of increasing doses of sedatives and decreasing tidal volume and PEEP (if tolerated) before considering using an additional bolus of cisatracurium (intravenous injection of 20 mg of cisatracurium). If this rapid, intravenous injection results in a decrease of the end-inspiratory plateau pressure by less than 2 cmH2O, a second injection of 20 mg of cisatracurium will be allowed. If after injection, the end-inspiratory plateau pressure does not decrease or decreases by less than 2 cm of water, cisatracurium boluses should not be administered again during the following 24-hour period.

We will prepare and recommend safety plans to patients receiving neuromuscular blockade that include eye care, positioning, and pressure ulcer monitoring.

In participating centers in which the bispectral index (BIS®, Aspect Medical Systems) system is currently available, the level of sedation under neuromuscular blockade could be alternatively titrated and monitored using the BIS®, with targeted value of 40-50, as proposed in a previous pilot trial of sevoflurane in ARDS[16]. Data from the subset of patients in whom this strategy will be applied (patients from both groups enrolled in selected centers, because of logistical considerations) will be used in a future sub-study to further assess the value of the bispectral index to monitor the level of sedation in ARDS patients under neuromuscular blockade.

The cisatracurium besylate infusion will be continued until PaO_2/FiO_2 exceeds 150 mmHg for 4 hours with FiO_2 <0.6[29,30]; then, light sedation will be targeted in both groups (RASS of 0 to -1, Ramsay of 2-3, or Riker of 3-4), with prompt sedation interruption whenever possible. Higher doses of sedation (inhaled or intravenous, depending on the randomization group) will be allowed for respiratory distress, ventilator dyssynchrony, or hypoxia.

In both groups, we will use standardized, step-wise, startup procedures to collect hemodynamic and respiratory safety data in the first 6 hours following randomization. These procedures will allow comparison of hemodynamics and respiratory measures during study startup between groups, and will avoid simultaneous PEEP and sedation titration, which would render interpretation of hypotensive or hypoxic episodes challenging. Close oversight of study initiation should be provided by an intensive care attending and/or designee.

Study startup procedures can be summarized as follows:

1. Initiate the mechanical ventilation strategy within 2 hours (see specific paragraph below)
2. Adjust sedation (with inhaled sevoflurane or intravenous propofol, depending on the randomization arm), to target sedation score (if not already at target
3. Start cisatracurium within 4 hours of randomization
4. Before increasing PEEP, a local investigator or designee will determine hemodynamic appropriateness for PEEP increase using the following as guidelines: mean arterial pressure >55 or systolic blood pressure >80 mmHg, no fluid bolus or vasopressor increase for greater than 15 minutes.

Instrumental dead space of the respiratory circuit will be reduced to the minimum in both arms.

We will protocolize low tidal volume ventilation, the strategy for weaning from mechanical ventilation, including spontaneous breathing trials, and a high PEEP strategy in both arms (Appendix C)[35].

We will use a simplified version of the ARDS network 6 mL/kg PBW lung-protective ventilation protocol[5] except that controlled modes of ventilation will be required during the period of neuromuscular blockade. If not already being used, a low tidal volume protocol for mechanical ventilation will be initiated within two hours of randomization in all patients. Using volume-controlled ventilation, tidal volume (Vt) will be set at 6 mL/kg (+/- 2 mL/kg) of PBW[32] and PEEP will be adjusted based on airway pressure and kept as high as possible without increasing the maximal inspiratory plateau pressure above 28 to 30 cmH_2O, such as in the Expiratory Pressure (Express) Study[31]; therefore, PEEP will be individually titrated based on plateau pressure, regardless of its effect on oxygenation in contrast to the PEEP/FiO_2 scales used in some studies[49,117].

We will recommend sites to wait at least 12 hours (as per PROSEVA[29]) before proning. As recommended by recent international guidelines[32], proning will be applied in patients with severe ARDS for more than 12 hours/day; proning will eventually be applied more than once, as per the treating clinicians.

Since the time a patient achieves unassisted ventilation affects some secondary endpoints, and because recent evidence-based consensus recommendations have identified a best practice for weaning, a weaning strategy will also be controlled by protocol rules in accordance with these evidence-based recommendations. This will assure similar weaning methods and provide potential benefit to both study groups. This weaning strategy is a simplified version of the protocolized weaning strategy used in prior ARDS Network studies (Appendix C2).

The AnaConDa-S will be removed from the breathing circuit as soon as inhaled sedation is interrupted. The AnaConDa-S should be removed from the breathing circuit for spontaneous breathing trial.

In the intervention arm, we will only allow deviation from the inhaled sedation strategy (interruption of sevoflurane administration and removal of the AnaConDa-S from the breathing circuit) if severe acidemia (pH <7.15) is present, in the absence of metabolic acidosis, and despite further tidal volume and/or respiratory rate increase, or if malignant hyperthermia or a bronchopleural fistula that is persistent despite drainage (to limit room exposure) develops under inhaled sedation (Appendix C1). In this situation, patients from the intervention arm will be switched to an intravenous sedation strategy using propofol.

We will allow deviation from the high PEEP strategy, for limited situations:

- If there is clinical concern that the use of high PEEP may be worsening oxygenation (e.g., oxygenation worsens with PEEP increases) at an FiO_2 ≥0.5 for more than 2 hours, clinicians may trial lower PEEP.
- If oxygenation worsens or is unchanged at the lower level of PEEP, PEEP should be raised back to the previous level.
- If hypotension and/or high Pplat (>30 cmH_2O) are present despite further tidal volume reduction, fluid boluses, and/or respiratory rate increase, lower PEEP may be used. It will then be allowed to reduce PEEP 2 cmH_2O every 5-15 minutes, until the physiologic parameters of concern have improved, as per the treating clinician and/or responsible investigator (e.g., reduce PEEP to the level that lowers plateau pressure to 30 cmH_2O). Later, the clinician tries to return PEEP to a level consistent with the mechanical strategy described above.

Lower PEEP may also be used if a study participant develops a pneumothorax, is deemed at high risk for barotrauma (e.g., known multiple pulmonary cysts or bullae) or as per the treating clinicians.

Fluid management during shock will be unrestricted. However, in patients not in shock, a conservative fluid approach will be recommended for all patients enrolled in the study. This conservative fluid management approach will represent a simplification of the algorithm utilized in the ARDS Network FACTT study (see Appendix D)[118].

Hyperglycemia has been associated with worst outcomes in ICU patients. Each site will use their own standard management, including institution-specific insulin drip protocols, to maintain blood sugar with a target upper blood glucose level ≤180 mg/dL. This range avoids marked hyperglycemia, while minimizing the risk of both iatrogenic hypoglycemia and other harms associated with a lower blood glucose target.

If PaO_2 ≥55 mmHg or SpO2 ≥88% with FiO_2 of 1 cannot be maintained, clinicians may employ alternate therapies (rescue procedures). Rescue procedures will be chosen according to the practice at the clinical site, and may include repeated recruitment maneuvers, inhaled nitric oxide, inhaled epoprostenol sodium, high frequency ventilation, ECMO, or neuromuscular blockade use after 48h from randomization. The participants will continue to be followed and included in the analysis on an intention-to-treat basis. The use of rescue procedures will be assessed as a secondary outcome.

Patients will be assessed at least at baseline, once a day during the first week after randomization, then on days 14, 21, 28, 90 and 365, as described in the Time-Events schedule (Appendix B). Special attention will be paid to patients with acute and/or chronic kidney injury, because of the theoretical risks associated with the

accumulation of sevoflurane metabolites.

Some **changes to data collection** (section 8) have been made in response to the current Covid-19 pandemics:

- Data will be collected remotely from the (electronic) patient health record by clinical research associates at each center, so that:
    - 1) **the risk for intensive care providers to be contaminated with the virus is minimized**, as no data will be collected at patient bedside
    - 2) **no additional work overload is needed from intensive care providers**, as their staffing may be very challenged in this threatening situation.
- **Relevant information on Covid-19**, such as specific interventions that may be delivered in these patients (such as antiviral or other specific therapies) will be collected.

This strategy will allow the rigorous collection of most data relevant to major patient outcomes (survival, duration of mechanical ventilation, indices of lung function, and safety data) while ensuring the safety of both intensive care providers and clinical research associates, and preventing interaction to the priority care patients will receive in participating ICUs.
However, and as a result, a larger amount of missing data can be expected for some variables that may be considered less relevant to collect from the (electronic) patient health record in the current pandemic context due to the surge in patients with Covid-19.

- **Background and baseline assessments visit (Inclusion visit – Day 0)**

    - **Background assessments**

1. Demographic and Admission Data (including age, sex, race)
2. Pertinent Medical History and Physical Examination (including Charlson co-morbidity score[119] and McCabe classification[120])
3. Height; gender; measured Body Weight (mBW); calculated predicted body weight (PBW); body mass index (BMI)
4. Time on ventilator prior to enrollment
5. Risk factors for ARDS (sepsis, suspected of confirmed Covid-19, aspiration, trauma, pneumonia, other)
6. Ever smoker (>100 cigarettes in lifetime)?
    - If Yes, current smoker?
    - If ever smoker, estimate pack years [Pack years = (# packs per day) x (number of years smoked)]
    - If former smoker, when quit?
7. Basic assessment of prior functioning
8. SARS-Cov2 (Covid-19) specific treatment, such as antiviral, anti-inflammatory (e.g., corticosteroids), immunomodulatory and/or other drug(s)...

- **Baseline assessments**

The following information will be recorded during the 24-hour interval preceding randomization. If more than one value is available for this 24-hour period, the value closest to the time of randomization will be recorded. If no values are available from the 24 hours prior to randomization, then values will be measured post randomization

but prior to initiation of sevoflurane (intervention arm) and within 4 hours (control arm). All values will be derived from clinically available data.

1. History and physical examination
   Vital signs: heart rate (beats / min), systemic systolic and diastolic blood pressure (mmHg), body temperature (°C)
2. Ventilator mode, set rate, actual rate, minute ventilation, tidal volume, FiO_2, PEEP, plateau and airway resistance
3. Administration of the following medications (name)
   a) Intravenous sedative
   b) Intravenous opioids
   c) Neuromuscular blocking agent
   d) Intravenous or enteral corticosteroids (≥20 methylprednisolone equivalents)
   e) Statin
   f) Antibiotic
   g) SARS-Cov2 (Covid-19) specific treatment, such as antiviral, anti-inflammatory (e.g., corticosteroids), immunomodulatory and/or other drug(s)...
4. Presumed site of infection, if sepsis is the etiology of ARDS
5. SAPS II and APACHE II scores, including the acute physiology components and laboratory values
6. APACHE II demographics plus history of: hypertension, prior myocardial infarction, congestive heart failure, peripheral vascular disease, prior stroke with sequelae, dementia, chronic pulmonary disease, arthritis, peptic ulcer disease
7. SOFA Score: cardiovascular, renal, respiratory, hepatic, and hematology organ function will be assessed using the SOFA methodology as described in Appendix G
8. Serum liver function tests: aspartate transaminase (AST), alanine transaminase (ALT), bilirubin, international normalized ratio (INR)
9. Treatment with vitamin K antagonists in the last week (Yes / No)
10. Pneumothorax at time of randomization (Yes / No)
11. Chest radiograph used to diagnose ARDS (Image file): Radiographic Assessment of Lung Edema (RALE) score[121]

- **Assessments during study**

  - **Respiratory monitoring during study startup**

In the intervention arm, the addition of the AnaConDa-S device to the breathing circuit will increase dead space to approximately 50 mL, (compared to the 36 mL dead space of small HME filters such as the DAR™ adult-pediatric electrostatic filter HME (small) (Covidien, Medtronic, Minneapolis, USA), and the theoretical risk of carbon dioxide rebreathing may expose the patient to the risk of developing severe acidemia (pH <7.15). Therefore, respiratory parameters, including tidal volume, respiratory rate, $PaCO_2$, arterial pH, FiO_2, PEEP, compliance of the respiratory system, airway resistance, plateau, will be closely monitored during the first 4 hours after randomization and from day 0 to day 7.

  - **Atmospheric pollution monitoring**

At each center, but in a limited number of patients (n=6-10 at each center) due to technical issues), we will monitor volatile atmospheric levels daily using dosimeters,

during the duration of inhaled sedation with sevoflurane

- **Reference measurements**

The following data will provide the basis for assessing protocol compliance and safety as well as between-group differences in several efficacy variables. Data for each of the variables will be recorded on the days shown in the Time-Events schedule (Appendix B) or until death, or discharge from the ICU. Values will be derived from clinically available data.

1. Sedation dosing in the intervention arm
    a. Time and infusion rate of sevoflurane administration when started
    b. Time of initiation of cisatracurium continuous infusion
    c. Reason and duration of sevoflurane administration hold
2. Sedation dosing in the intervention arm

Name of any sedative agent received intravenously in the first 7 days after randomization.

In both arms, time of loading cisatracurium dose, reason and duration of infusion hold during the first 48 hours, of other neuromuscular blocking agent(s) during the first 120 hours, and will be recorded.

- **Reference measurements (Daily)**

The following parameters will be measured and recorded between 4:00 and 10:00 A.M. using the values closest to 8:00 A.M. on the days specified in the Time-Events schedule. The following conditions will be ensured prior to measurements: no endobronchial suctioning for 10 minutes; no invasive procedures or ventilator changes for 30 minutes. All vascular pressures will be zero-referenced to the mid-axillary line.

1. If receiving positive pressure ventilation: ventilator mode, set rate, actual rate, minute ventilation, tidal volume, FiO_2, PEEP, compliance of the respiratory system, airway resistance, plateau
2. PaO_2, $PaCO_2$, arterial pH, and SpO_2 on study days 1-7

Values for the following variables will be recorded for the dates shown in the Time-Events Schedule. If the measurements are not obtained during the 6-hour reference interval (4:00 to 10:00 A.M.), then the value obtained closest in time to the reference interval on the respective date will be recorded. If more than one value is obtained during the reference interval, then the value obtained closest to 8:00 A.M. will be recorded.

1. Rescue procedures used
    a) Inhaled nitric oxide
    b) Epoprostenol sodium
    c) High-frequency ventilation
    d) ECMO
    e) Neuromuscular blockade use after 48h from randomization
2. Serum electrolytes and glucose
3. Administration of the following medication infusions:
    a) Intravenous opioids
    b) Enteral or intravenous corticosteroids (≥20 methylprednisolone equivalents)
4. Sedation score: If RASS <-1 (or Riker <3, Ramsay >3), and sedation given, list reason given
5. Was a sedation interruption performed (Yes / No)?
6. Modified SOFA
7. Serum liver function tests: AST, ALT, bilirubin, INR
8. Central venous pressure (CVP) if available

9. Hemodynamic measures: mean arterial pressure, maximal dose of infused norepinephrine or other vasopressor, serum lactate level, supraventricular tachycardia (SVT) or new onset atrial fibrillation on study days 1-7
10. KDIGO criteria for acute kidney injury[26] on study days 1-7 (Appendix H)

- **Other reference measurements**

1. ICU-acquired delirium

The Confusion Assessment Method for the ICU (CAM-ICU)[103] will be assessed daily from study entry to study day 7, death or ICU discharge, whichever comes first.

- **Specimen collection**

<u>In all patients</u>

Plasma and urine will be collected within 2 hours of randomization (day 0) and on days 1, 2, 4, 6 and 14 or ICU discharge (whichever occurs first) for both arms, frozen (-80°C), and stored for further measurements (at each pre-specified timepoint) of biological markers of a hyperinflammatory ARDS phenotype (plasma IL-8, TNF-receptor 1, bicarbonates)[22], of ventilator-induced lung injury (plasma IL-6)[5], of lung endothelial injury (plasma angiopoietin (ANG)-2)[93,94], of epithelial injury and AFC (plasma sRAGE)[17,95] , and of acute kidney injury (urine TIMP-2 and IGFBP-7[96]). In addition, total fluoride and hexafluoroisopropanol levels will be measured in plasma samples.

Total blood volume for these draws is approximately 4 mL/day (1 EDTA tube of 4 mL), for a total of approximately 24 mL. Samples will be centrifuged (3,000 rpm at 4°C for 10 min in a refrigerated centrifuge), aliquoted (supernatant from each tube dispatched in 3 aliquots), before being frozen at -80°C for future proteomic studies.

Total urine volume for these draws is approximately 10 mL/day (1 Monovette urine tube), for a total of approximately 60 mL. Urine samples will be centrifuged (at 3,000 rpm at 4°C for 10 min in a refrigerated centrifuge), aliquoted (supernatant dispatched in 3 aliquots), and frozen at -80°C.

When consent obtained (specific emergent procedure of waiver of consent only when the patient's next of kin cannot be reached during the short time window for enrollment) we will also collect at baseline (day 0) and on day 2 an additional 5 mL/day (2 EDTA tubes of 2.5 mL each) of whole blood that will be frozen (-80°C) for future DNA/RNA studies.

<u>In a total of 100 patients (n=50 patients from each group) enrolled in selected centers because of logistical considerations</u>

Undiluted pulmonary edema fluid samples will be collected at study entry and 24 hours later. Undiluted edema fluid samples (3-5 mL maximum) will be obtained without bronchoscopy, through a soft 14-Fr-gauge suction catheter (PharmaPlast, Maersk Medical, Denmark) advanced into a wedged position in a distal bronchus via the endotracheal tube; edema fluid will then be collected in a suction trap by gentle suction, centrifuged (3,000 rpm at 4°C for 10 min), aliquoted (supernatant from each tube dispatched in 2 aliquots), before being frozen at -80°C.

<u>In a total of 60 patients (n=30 patients from each group) enrolled in selected centers because of logistical considerations</u>

Heat moisture exchanger and AnaConDa-S filters will be collected at 24 hours in those patients randomized to the control and intervention groups (n=30 patients from

each group), respectively. Condensed fluid from heat moisture exchanger (used in the intravenous sedation group) and AnaConDa-S (used in the inhaled sedation group) filters will be collected 24 hours after study treatment initiation. Filters will be transported to the laboratory on ice and centrifuged at 2,000 x g for 10 minutes to collect condensed fluid that will be subsequently aliquoted (4 aliquots of 1mL each) and stored at -80°C for further analysis.

These samples will be used for future analyses:

- of the biological effects of sevoflurane on lung injury and repair
- of biomarker measurement in the filter fluid as representative of biomarker measurement in the distal airspace fluid in ARDS patients receiving inhaled sedation[97]

<u>In a total of 25 patients enrolled in 5 selected centers (because of logistical considerations, expected number of patients per center = 5)</u>

Additional alveolar fluid samples (in intubated patients, through bronchoalveolar lavage) within 48 hours from study entry and between day 4 and day 6. Alveolar samples (a sample of approximately 10-20 mL obtained with bronchoscopy, after the instillation of 40 to 150 mL sterile isotonic saline) will be transferred in Falcon® tubes. Samples will be centrifuged (at 3,000 rpm at room temperature for 10 min in a centrifuge), aliquoted (supernatant dispatched in 3 aliquots), and frozen at -80°C, for future proteomics analyses.

Study samples will be prepared and stored at each participating center before being transferred to and stored in a central repository (*Centre de Ressources Biologiques Auvergne* (CRB-A), CHU Clermont-Ferrand), in accordance with good laboratory practices, prior to biological analyses. This storage is planned for the duration of the SESAR trial (48 months) and thereafter. Samples will be identified by a coded number during all phases, including shipment and storage in the central repository.

The funding needed to perform these biological analyses will be requested independently of the funding of the clinical trial itself.

<u>**It is anticipated that the biological collection may not be done as initially planned (i.e., without alveolar samples at least), or even not done at all, during the Covid-19 pandemics, in order to minimize the risk for intensive care providers to be contaminated with the virus and to ensure no additional work overload is needed from intensive care providers.**</u>

Because the current Covid-19 outbreak and ICU surge may be at high risk of staff shortage with extreme material and organizational difficulties for healthcare providers to take care of all patients admitted to the ICU, priority will be given, whenever possible, to the collection of plasma samples because those samples are very likely those that will more easily better inform mechanistic investigations on the effects of inhaled sevoflurane on inflammation and acute lung injury.

Importantly, neither alveolar fluid (undiluted edema fluid or through bronchoalveolar lavage) nor HME/AnaConDa sampling will be mandatory during the Covid-19 pandemics and, when performed, sampling will aim at the virus spreading and protect all intensive care providers, as currently recommended (<u>Recommandations d'experts SRLF-SFAR-SFMU-GFRUP-SPILF sur la prise en charge en réanimation des patients en période d'épidémie à SARS-CoV2</u>).

- **Assessments after hospitalization**

We will assess seven measures after hospitalization:

1. Disability using Katz Activities of Daily Living (ADL)[106]
2. Health-Related Quality of Life (including utilities): Short Form-36 (SF-36)
3. Self-rated health: 1 standard item
4. Pain-interference: 1 standard item
5. Post-traumatic Stress-like Symptoms: Post-Traumatic Stress Symptoms (PTSS-14)[107,108], Hospital Anxiety and Depression Scale (HADS)[109,110]
6. Cognitive function: the Alzheimer's Disease 8 (AD8)[111]
7. Subsequent return to work, hospital and ED use, and location of residence

These measures will be collected through telephone interviews with patients or their LARs. Informed consent process will include text to facilitate future ancillary long-term follow up studies and data collection. All will be obtained at 3 and 12 months. Most will be obtained from proxies when necessary, except as noted for self-rated health, pain interference and post-traumatic stress-like symptoms.

The Katz ADL is associated with multiple health outcomes among community-dwelling elders[129], and valid among nursing home residents[130]. The Lawton IADL is probably the most widely used self-report or informant-report IADL instrument. These assess a range of common functional activities, from walking and toileting to managing money and cooking meals. These scales have been specifically shown to perform well when assessed by proxies for ICU survivors[131]. We will not use the Visual Analog Scale as these follow-ups will be phone administered and the VAS is not necessary for health economic analysis.

Self-rated health and pain-interference are two common single-item scales that are widely used. Because of the highly subjective nature of these domains, these will only be assessed by self-respondents.

We will capture return to work status using the Improving Care of ALI Patients (ICAP) study questionnaire[132]. The Improving Care of Acute Lung Injury Patients employment instrument (ICAP-12) was designed to determine pre- and post-morbid employment status in a multi-center observational study of ARDS survivors[132,133] and used externally in the NHLBI ARDS Network's ARDS Long-term Outcomes Study (ALTOS) cohorts[134,135]. We will also ask about recent hospital and Emergency Department use, and whether the patient is residing in a nursing home, at home, or elsewhere.

We will collect contact information for the patient and alternative contact information on up to 2 individuals.

6.2. Diagram of the study

The CONSORT diagram of the study is provided in Appendix K.

6.3. Differences with routine clinical practice

With the exception of a sedation strategy based on inhaled sevoflurane in the intervention arm, the management of patients will be performed according to routine clinical practice for ICU patients with moderate-severe ARDS, and according to the

expertize of the staff at each study center.

A description of all acts and evaluations performed on patients is detailed in Section 6.1 above and in the Time-events schedule (Appendix B). Notably, some evaluations are specific to the study:

- Evaluation of delirium (CAM-ICU): daily from day 0 to day 7
- Plasma and urine collection: at study entry and on days 1, 2, 4, 6 and 14 or ICU discharge (whichever occurs first)
- Whole Blood Collection for future DNA/RNA and macrophage activation studies: on day 0 and day 2
- Evaluation of disability (KATZ ADL, health-Related Quality of Life (SF-36), self-rated health, pain-interference, cognitive function (AD-8), subsequent return to work, hospital and ED use, and location of residence: on days 90 and 365
- Evaluation of post-traumatic stress-like symptoms (PTSS-14, HADS): on days 90 and 365
- Evaluation of vital status: on days 7, 14, 28, 90 and 365
- Undiluted pulmonary edema fluid samples will be collected at study entry and 24 hours later in a total of 100 patients (n=50 patients from each group) samples will be collected at study entry and 24 hours later in some patients enrolled in selected centers (because of logistical considerations). In addition, heat moisture exchanger and AnaConDa-S filters will be collected at 24 hours in those patients randomized to the control and intervention groups (n=30 patients from each group), respectively. These samples will be used for future analyses:
  - of the biological effects of sevoflurane on lung injury and repair
  - of biomarker measurement in the filter fluid as representative of biomarker measurement in the distal airspace fluid in ARDS patients receiving inhaled sedation[97]

It is anticipated that the biological collection may not be done as initially planned (i.e., without alveolar samples at least), or even not done at all, during the Covid-19 pandemics, in order to minimize the risk for intensive care providers to be contaminated with the virus and to ensure no additional work overload is needed from intensive care providers.

Because the current Covid-19 outbreak and ICU surge may be at high risk of staff shortage with extreme material and organizational difficulties for healthcare providers to take care of all patients admitted to the ICU, priority will be given, whenever possible, to the collection of plasma samples because those samples are very likely those that will more easily better inform mechanistic investigations on the effects of inhaled sevoflurane on inflammation and acute lung injury.

Importantly, neither alveolar fluid (undiluted edema fluid or through bronchoalveolar lavage) nor HME/AnaConDa sampling will be mandatory during the Covid-19 pandemics and, when performed, sampling will aim at minimizing the virus spreading and protect all intensive care providers, as currently recommended (Recommandations d'experts SRLF-SFAR-SFMU-GFRUP-SPILF sur la prise en charge en réanimation des patients en période d'épidémie à SARS-CoV2).

6.4. Expected duration of participation for each patient and study schedule

Expected duration of the study: 36-month (recruitment period)
Start of the study (first inclusion): May 2020

End of the study (follow-up of the last patient): 4 years
Total duration of participation for a patient: 365 days

7. Products (treatment/medical device/other) used for the study

7.1. Description of treatments

After randomization, the low tidal volume protocol will be initiated (if not already being used) in patients from both arms. Deep sedation (as assessed with validated sedation scores) followed by neuromuscular blockade with cisatracurium besylate will be initiated (if not already applied) within four hours of randomization in both arms. All participating centers have existing protocols and order sets for routine sedation management, glucose control, septic shock resuscitation, deep venous thrombosis prophylaxis, and other aspects of background care.

Because this study aims at assessing the efficacy and safety of sedation with inhaled sevoflurane in improving a composite outcome of mortality and time off the ventilator at 28 days in patients with moderate-severe ARDS in comparison to a control group receiving intravenous sedation, the two study arms will only differ with regards to the sedation strategy:

- Control arm: intravenous propofol will be used for sedation, as already routinely used in participating ICUs.
- Intervention arm: inhaled sevoflurane, as vaporized via the Anesthesia Conserving Device (AnaConDa-S, Sedana Medical, Danderyd, Sweden) will be used for sedation. AnaConDa-S will be placed between the endotracheal tube and Y-piece of the ventilator breathing circuit (**Figure 1**), and sevoflurane will be infused into the device for vaporization before inhalation.

We will protocolize mechanical ventilation, the strategy for weaning from mechanical ventilation, including spontaneous breathing trials, in both arms (Appendix C).

Since the time a patient achieves unassisted ventilation affects some secondary endpoints, and because recent evidence-based consensus recommendations have identified a best practice for weaning, a weaning strategy will also be controlled by protocol rules in accordance with these evidence-based recommendations. This will ensure similar weaning methods and provide potential benefit to both study groups. This weaning strategy is a simplified version of the protocolized weaning strategy used in prior ARDS Network studies (Appendix C2).

7.2. Dosage, administration and duration of treatment

Patients will receive a cisatracurium besylate bolus of 15 mg, followed by a continuous infusion of 37.5 mg/hour for a maximum of 48 hours[48,49]. We chose this fixed, relatively high dosage for simplicity (train-of-four titration imperfect and with limited evidence base[114–116]) and to help ensure effective neuromuscular blockade (clinical observation and train-of-four monitoring can lead to under-dosing). This dosage is the same as used in the ACURASYS trial[48]. We chose cisatracurium as its

metabolism is independent of hepatic and renal function.

The cisatracurium besylate infusion will be continued until PaO_2/FiO_2 exceeds 200 mmHg for 4 hours with FiO_2 <0.6[29,30]; then, light sedation will be targeted in both groups (RASS of 0 to -1, Ramsay of 2-3, or Riker of 3-4), with prompt sedation interruption whenever possible. Higher doses of sedation (inhaled or intravenous, depending on the randomization group) will be allowed for respiratory distress, ventilator dyssynchrony, or hypoxia.

In participating centers in which the bispectral index (BIS®, Aspect Medical Systems) system is currently available, the level of sedation under neuromuscular blockade could be alternatively titrated and monitored using the BIS®, with targeted value of 40-50, as proposed in a previous pilot trial of sevoflurane in ARDS[16]. Data from the subset of patients in whom this strategy will be applied (patients from both groups enrolled in selected centers, because of logistical considerations) will be used in a future sub-study to further assess the value of the bispectral index to monitor the level of sedation in ARDS patients under neuromuscular blockade.

In the intervention arm, sevoflurane will be administered through the recently developed miniaturized AnaConDa-S (Sedana Medical, Danderyd, Sweden). Sevoflurane infusion rate will be adapted from manufacturer's instructions in order to reach the targeted sedation. $F_I$sevo, $F_E$sevo and $EtCO_2$ will be continuously monitored using a separate bedside gas analyzer. AnaConDa-S has a built-in carbon layer that allows for more than 80% recycling of the expired agent; residual expired gas will be scavenged following manufacturer's instructions using an active carbon filter[66]. As recommended by the manufacturer, AnaConDa-S will be replaced every 24 hours.

The addition of the AnaConDa-S device to the breathing circuit will increase dead space to approximately 50 mL, (compared to the 36 mL dead space of small HME filters such as the DAR™ adult-pediatric electrostatic filter HME (small) (Covidien, Medtronic, Minneapolis, USA), and the theoretical risk of carbon dioxide rebreathing may expose the patient to the risk of developing hypercapnic acidosis. Sevoflurane administration will be interrupted and the AnaConDa-S will be removed from the breathing circuit) if severe acidemia (pH <7.15) is present, in the absence of metabolic acidosis, and despite tidal volume and/or respiratory rate increase, or if malignant hyperthermia or a bronchopleural fistula that is persistent despite drainage (to limit room exposure)develops under inhaled sedation (Appendix C1). In this situation, patients from the intervention arm will be switched to an intravenous sedation strategy.

In each group, patients will receive the allocated sedation strategy from randomization until sedation can be definitely interrupted or until day 7, whichever occurs first. If sedation needs to be applied again within 7 days after randomization, the sedative agent to use will be based on the randomization arm (inhaled sevoflurane vs. intravenous propofol). After day 7, decisions on further decisions use of sedative agents, including type of and dosing of the agent, will be as per the treating clinicians.

All interventions, except for the sedation choice (either with intravenous propofol (considered as current standard practice) or with inhaled sevoflurane (study intervention)) are based on current guidelines on the management of severe ARDS in general and of patients with Covid-19 in particular (Surviving Sepsis Campaign: Guidelines on the Management of Critically Ill Adults with Coronavirus Disease 2019; The Australian and New Zealand Intensive Care Society (ANZICS) COVID-19 Guidelines; Recommandations d'experts SRLF-SFAR-SFMU-GFRUP-SPILF sur la prise en charge en réanimation des patients en période d'épidémie à SARS-CoV2).

In addition, details have been added to the interventions in both study arms, especially with regards to viral isolation and filtration methods, which are of paramount importance in the management of Covid-19 patients:

- The virus filtration capacity of the AnaConDa is >99.9% for 27 nm particles, less than one quarter of the size of the SARS-CoV-2 virus (120-160 nm), implying very low risk for SARS-CoV-2 virus passing the AnaConDa (**Appendix A of the accompanying "NOTICE - SESAR trial v5 (Covid-19)" document**). **The AnaConDa device is therefore safe to use in patients with Covid-19**.
- As currently recommended (Recommandations d'experts SRLF-SFAR-SFMU-GFRUP-SPILF sur la prise en charge en réanimation des patients en période d'épidémie à SARS-CoV2), **an additional "high-efficiency particulate air" (HEPA) filter will be connected to the expiratory port of the breathing circuit, to protect the ventilator for exhaled virus entry in all patients with suspected or confirmed Covid-19 (Appendix B of the accompanying "NOTICE - SESAR trial v5 (Covid-19)" document**).
    - In patients randomized to receive inhaled sedation through the AnaconDa device, it is therefore possible to return the waste gas from the gas monitor in the expiratory side of the breathing circuit, before the additional virus filter, by using the standard bypass connector (normally placed at the FlurAbsorb filter) (**Appendix C of the accompanying "NOTICE - SESAR trial v5 (Covid-19)" document**).
    - Exhaled air is sampled to the anesthetic gas monitor from the patient side of the filter and is thus not filtered. However, most gas monitors that will be used in SESAR have viral filters in the water trap, with different degrees of virus filtering capacity (**Appendix D of the accompanying "NOTICE - SESAR trial v5 (Covid-19)" document**).
- **In patients from the control group (intravenous sedation with propofol), a heat and moisture exchanger (HME)-HEPA filter will be used and connected between the endotracheal tube and the Y-piece of the respiratory circuit**, as initially planned in the previous version of the protocol.

7.3. Presentation of the study drugs

7.3.1. Experimental treatment

- **Sevoflurane** (Abbvie Inc., North Chicago, Illinois, USA)

Sevoflurane, USP, volatile liquid for inhalation, a nonflammable and nonexplosive liquid administered by vaporization, is a halogenated general inhalation anesthetic drug. Sevoflurane, USP is fluoromethyl 2,2,2,-trifluoro-1-(trifluoromethyl) ethyl ether and its structural formula is:

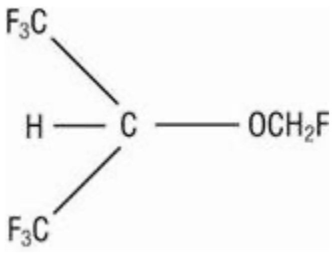

Sevoflurane, USP, Physical Constants are:	
Molecular weight	200.05
Boiling point at 760 mmHg	58.6°C
Specific gravity at 20°C	1.520 - 1.525
Vapor pressure in mmHg	157 mmHg at 20°C<br>197 mmHg at 25°C<br>317 mmHg at 36°C
Distribution Partition Coefficients at 37°C:	
Blood/Gas	0.63 - 0.69
Water/Gas	0.36
Olive Oil/Gas	47 - 54
Brain/Gas	1.15
Mean Component/Gas Partition Coefficients at 25°C for Polymers Used Commonly in Medical Applications:	
Conductive rubber	14.0
Butyl rubber	7.7
Polyvinylchloride	17.4
Polyethylene	1.3

The full package insert, which will serve as the Investigator Brochure for this trial, can be found here:

http://dailymed.nlm.nih.gov/dailymed/drugInfo.cfm?setid=ea8bf997-2c71-4014-b18d-4f7ab45dfa19 (English version)

http://www.ansm.sante.fr/searchengine/detail/(cis)/67329056 (French version)

- **<u>Miniaturized Anesthesia Conserving Device</u>** <u>(**AnaConDa-S**; Sedana Medical, Danderyd, Sweden)</u>

The new 50 mL AnaConDa-S (Sedana Medical, Danderyd, Sweden) is intended for administration and reflection of isoflurane and sevoflurane to invasively ventilated patients. The AnaConDa-S functions as an effective passive heat and moisture exchanger comparable to all similar volume HME devices on the market. The 50 mL AnaConDa-S will be used for the same clinical conditions or clinical indications than the existing 100 mL AnaConDa. The AnaConDa-S is a small device that is inserted between the endotracheal tube and the Y-piece, in the ventilator circuit. The simple design of the AnaConDa-S incorporates a unique high capacity miniature vaporizer, along with a high efficiency conserving medium. Essentially, the AnaConDa-S makes the CO_2 absorber, and one-way valves, normally included in an Anesthesia machine, unnecessary. The AnaConDa-S makes it possible to deliver anesthetic agents in an easy and safe way with a standard ventilator. The AnaConDa-S is intended for single use only and needs to be replaced every 24 hours. Administration of isoflurane and sevoflurane using AnaConDa-S should only be done in a setting fully equipped for the monitoring and support of respiratory and cardiovascular function and by persons specifically trained in the use of inhalational anesthetic drugs. The AnaConDa-S will be used on adult male and female patients with a tidal volume between 200 mL and 800 mL.

The AnaConDa-S evaporates and reflects Isoflurane and Sevoflurane in same manner and to the same concentration as the 100 mL device within its intended Vt range. The desired blood gas concentration in the patient (F_E) value will be titrated by adjusting the syringe pump in the same manner as the 100 mL AnaConDa. The reflection efficiency will be similar to within 2% points which is clinically insignificant.

The set-up of the AnaConDa-S system will follow the instructions for use provided by the manufacturer, including a number of specific security elements that minimize, if not eliminate, the risk of cognitive and manipulation errors. Each step of the AnaConDa-S system set-up will be described in both a detailed brochure specific to the study and a video tutorial provided to every medical and paramedical staff member from participating ICUs prior to the study.

These specific security elements are described below:

- Only medical devices which bear the CE mark and which comply with its applicable international standards, may be used
- AnaConDa-S must be used with the following equipment:
    - o AnaConDa-S syringe specific to the system (REF 26022). The AnaConDa-S syringe is the same dimension as the Becton Dickinson Plastipak and Monoject 50, 50/60, and 60 ml syringes. However, it has a unique coupling to fit the connector on the agent line of the AnaConDa-S, so that, for instance, this syringe cannot be, by design, connected to vascular lines. There are boxes to tick on the labelling, to indicate which volatile agent is being used (isoflurane or sevoflurane), and a specific location to indicate the date of filling. The syringes can be pre-filled and stored up to 5 days if stored in a dark environment at room temperature. The materials composing the AnaConDa-S system (including the syringe, agent line, and vaporizer filter) are specifically designed for the use of halogenated agents isoflurane or sevoflurane and to prevent their degradation.

- A filling adapter that is specific to the system (REF 26042, 26064) and used to fill the AnaConDa-S syringe. For safe filling of the AnaConDa-S syringe, only the correct filling adaptor can be used ; there are 2 types, one for standard threading bottles (REF 26064) and one for Sevorane (REF 26042) from AbbVie with QuikFil closure.

- Syringe pump with settings for BD Plastipak or Monoject 50, 50/60 or 60 ml syringes.

- A ventilator, an anesthetic gas monitor, which displays concentrations of carbon dioxide and anesthetic gases, and a gas scavenging system.

<u>Details for AnaConDa-S</u>:

- Pump rate required to reach 1.2% F_E at 500 mL Vt x respiratory rate (RR) of 15 b/min = 3.2 mL/hour
- Reflection Efficiency – 88%
- The Heat and Moisture characteristics of the 50ml device is similar to the 100 mL device and also compares favorably with specifications for similar size commercial HME devices.
- Resistance to Gas Flow @ 60 L/min – 3.0 cmH_2O
- Moisture Loss at 500 mL x 15 breaths/min – 5 mg/L
- Corresponding Moisture Output – 32 mg H_2O/L
- Moisture Loss at 800 mL x 12 breaths/min – 6.5 mg/L
- Corresponding Moisture Output – 31mg H_2O/L
- Length – 130 mm
- Max Width – 52 mm
- Depth – 33.5
- Weight – 35 g
- Dead Space – 50 mL
- Bacterial Filtration – 99.999
- Viral Filtration – 99.98 - The virus filtration capacity of the AnaConDa is >99.9% for 27 nm particles, less than one quarter of the size of the SARS-CoV-2 virus (120-160 nm), implying very low risk for SARS-CoV-2 virus passing the AnaConDa (see **<u>Appendix A of the accompanying “NOTICE - SESAR trial v5 (Covid-19)” document</u>**). **The AnaConDa device is therefore safe to use in patients with Covid-19**.
- Date of CE mark: January 2017

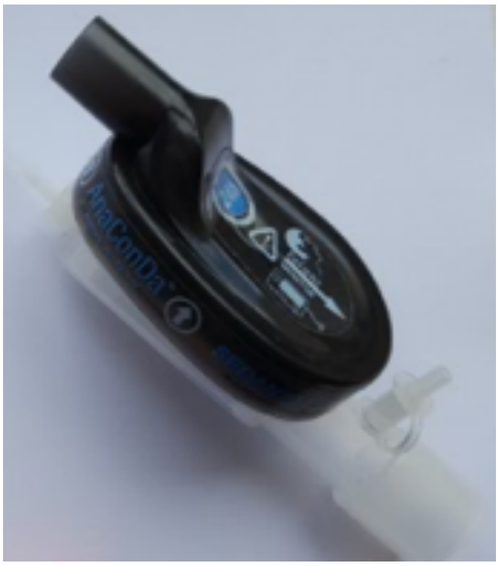

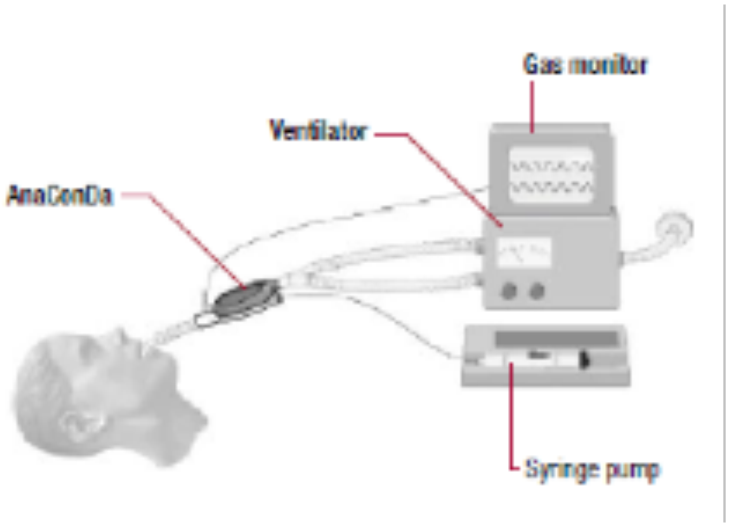

The full package insert of the AnaConDa-S, which will serve as the Investigator Brochure for this trial, can be found here:

http://www.sedanamedical.com/files/IFU_Anaconda26000_English_151101.pdf
(English version)

http://www.sedanamedical.com/files/IFU_Anaconda26000_French_151101.pdf
(French version)

7.3.2. Non-experimental treatment

NIMBEX (cisatracurium besylate) is a nondepolarizing skeletal muscle relaxant for intravenous administration. Compared to other neuromuscular blocking agents, it is intermediate in its onset and duration of action. Cisatracurium besylate is one of 10 isomers of atracurium besylate and constitutes approximately 15% of that mixture. Cisatracurium besylate is [1R-[1α,2α(1'R*,2'R*)]]-2,2'-[1,5-pentanediylbis[oxy(3-oxo-

3,1-propanediyl)]]bis[1-[(3,4-dimethoxyphenyl)methyl]-1,2,3,4-tetrahydro-6,7-dimethoxy-2-methylisoquinolinium] dibenzenesulfonate. The molecular formula of the cisatracurium parent bis-cation is C53H72N2O12 and the molecular weight is 929.2. The molecular formula of cisatracurium as the besylate salt is C65H82N2O18S2 and the molecular weight is 1243.50.

The full package insert, which will serve as the Investigator Brochure for this trial, can be found here:

http://dailymed.nlm.nih.gov/dailymed/drugInfo.cfm?setid=3db3b76c-3e5a-456e-46a8-456fde1e6195 (English version)

http://agence-prd.ansm.sante.fr/php/ecodex/rcp/R0223694.htm (French version)

In the control arm, intravenous sedation will be delivered using continuous intravenous infusion of propofol (10 mg/ml or 20 mg/ml), as already routinely used in participating ICUs, in a global strategy aimed at avoiding benzodiazepines and based on the latest "Clinical Practice Guidelines for the Prevention and Management of Pain, Agitation / Sedation, Delirium, Immobility, and Sleep Disruption in Adult Patients in the ICU » as published in Critical Care Medicine in 2018 (doi 10.1097/CCM.0000000000003299).
Propofol injectable emulsion is a sterile, nonpyrogenic emulsion containing 10 mg/mL of propofol suitable for intravenous administration. Propofol is chemically described as 2,6-diisopropylphenol. The molecular weight is 178.27 and the structural formula is $C_{12}H_{18}O$:

Propofol injectable emulsion is an intravenous general anesthetic and sedation drug. In the Intensive Care Unit (ICU), propofol injectable emulsion can be administered to intubated, mechanically ventilated adult patients to provide continuous sedation and control of stress responses only by persons skilled in the medical management of critically ill patients and trained in cardiovascular resuscitation and airway management.

The full package insert, which will serve as the Investigator Brochure for this trial, can be found here:

https://dailymed.nlm.nih.gov/dailymed/drugInfo.cfm?setid=28d7ba00-f824-4e55-139a-03f509c099db (English version)

http://agence-prd.ansm.sante.fr/php/ecodex/rcp/R0216298.htm (French version)

In the control group, heat and moisture exchanger filters will be those already in routine used in participating ICUs, such as the DAR™ adult-pediatric electrostatic filter HME (small) (Covidien, Medtronic, Minneapolis, USA):

Features:
- Combined filter / HME reduces set up time, and reduces dead space and weight on endotracheal tube and circuit while increasing efficiency
- Hygroscopic membrane traps patient's moisture providing effective airway humidification
- Low resistance to airflow reduces work of breathing

- Effective moisture control reduces the risk of colonization in breathing tubes and reduces sampling line blockage
- End tidal CO_2 sampling port offers convenient access to airway gases
- ISO standard 15 mm and 22 mm fitting connects with breathing system
- Supplied clean and individually packaged

Indications for usage: for adult (recommended tidal volume >150 ml), single patient use on anesthetized patients and respiratory care patients who require a breathing circuit. The product will be replaced at least once every 24 hours, as intended by the manufacturer and good practice.

Technical details for DAR™ adult-pediatric electrostatic filter HME (small):

- Tidal volume range: 150-1200 mL
- Resistance to Gas Flow @ 60 L/min – 2.1 cmH_2O
- Moisture Loss at 500 mL – 18mg H_2O/L
- Corresponding Moisture Output at 500 mL – 9mg H_2O/L
- Weight – 19 g
- Dead Space – 36 mL
- Bacterial Filtration – ≥99.999%
- Viral Filtration – ≥99.999%
- NaCl filtration – ≥97.100%

The full package insert, which will serve as the Investigator Brochure for this trial, can be found here:

https://www.medtronic.com/covidien/en-us/products/mechanical-ventilation/filters/dar-filters.html (English version)

https://www.theramed.ch/media/products/Intensivpflege/Beatmungszubehoer/Beatmungsfilter-HME-DAR/Doc_Beatmungsfilter_f.pdf (French version)

7.4. Drugs and treatment allowed and not allowed during the study

The study protocol stresses that patients should be managed according to the staff's expertize at each center and to routine clinical practice to minimize interference with the trial intervention. Treatments will be administered according to Good Clinical Practice. There will be no treatment/drugs forbidden during the study.

8. Data collection and registration

Some **changes to data collection** (section 8) have been made in response to the current Covid-19 pandemics:

- Data will be collected remotely from the (electronic) patient health record by clinical research associates at each center, so that:
  - 1) **the risk for intensive care providers to be contaminated with the virus is minimized**, as no data will be collected at patient bedside

        - 2) **no additional work overload is needed from intensive care providers**, as their staffing may be very challenged in this threatening situation.
    - **Relevant information on Covid-19**, such as specific interventions that may be delivered in these patients (such as antiviral, anti-inflammatory (e.g., corticosteroids), immunomodulatory and/or other therapies) will be collected.
    - A **"core dataset" within the SESAR eCRF** (**provided as a separate document**), including only variables that may be the most clinically relevant in the emergency context of Covid-19 outbreak will be blindly released and analyzed for the additional interim analyses planned in response to the Covid-19 outbreak. The DMSC will be empowered to communicate these interim findings directly to public health authorities if the committee thinks the trial produces any results that may be of relevance to public health with regard to the current Covid-19 outbreak.

This strategy will allow the rigorous collection of most data relevant to major patient outcomes (survival, duration of mechanical ventilation, indices of lung function, and safety data) while ensuring the safety of both intensive care providers and clinical research associates, and preventing interaction to the priority care patients will receive in participating ICUs.

However, and as a result, a larger amount of missing data can be expected for some variables that may be considered less relevant to collect from the (electronic) patient health record in the current pandemic context due to the surge in patients with Covid-19.

Data will be registered into the electronic web-based (Clinsight) report form (eCRF) by trial or clinical personnel under the supervision of the trial site investigators at each participating center. Paper CRF will be used in case of technical difficulties with the eCRF. Data registration will be monitored by trained research coordinators.

The following data will be registered:

- **<u>Pre-randomization and baseline characteristics</u>:**
    - Date (XX/YY/20ZZ) of admission to hospital and to the ICU
    - Date (XX/YY/20ZZ) of moderate-severe ARDS onset
    - Demographic data (age, sex, weight, height, body mass index)
    - History, physical examination and vital signs: heart rate (beats / min), systemic systolic and diastolic blood pressure (mmHg), body temperature (°C)
    - Comorbidities (arterial hypertension Y/N, diabetes Y/N, active smoking Y/N, alcohol abuse Y/N, chronic obstructive pulmonary disease Y/N, cancer Y/N)
    - Ventilator mode, set rate, actual rate, minute ventilation, tidal volume, FiO_2, PEEP, plateau and airway resistance
    - Administration of the following medications (name)
        - Intravenous sedatives
        - Intravenous opioids
        - Neuromuscular blocking agent
        - Intravenous or enteral corticosteroids (≥20 methylprednisolone equivalents)

        - Statin
        - Antibiotic
        - SARS-Cov2 (Covid-19) specific treatment, such as, anti-inflammatory (e.g., corticosteroids), immunomodulatory and/or other drug(s)...
    - Presumed site of infection, if sepsis is the etiology of ARDS
    - SAPS II and APACHE II scores, including the acute physiology components and laboratory values
    - APACHE II demographics plus history of: hypertension, prior myocardial infarction, congestive heart failure, peripheral vascular disease, prior stroke with sequelae, dementia, chronic pulmonary disease, arthritis, peptic ulcer disease
    - SOFA Score: cardiovascular, renal, respiratory, hepatic, and hematology organ function will be assessed using the SOFA methodology as described in Appendix G
    - Serum liver function tests: aspartate transaminase (AST), alanine transaminase (ALT), bilirubin, international normalized ratio (INR)
    - Treatment with vitamin K antagonists in the last week (Yes / No)
    - Pneumothorax at time of randomization (Yes / No)
    - Chest radiograph used to diagnose ARDS (Image file to upload)
    - Date (XX/YY/20ZZ) of initiation of non-invasive mechanical ventilation, if any
    - Date (XX/YY/20ZZ) of initiation of high flow oxygen through a nasal cannula, if any
    - Date (XX/YY/20ZZ) of initiation of invasive mechanical ventilation, if any
    - Time on ventilator prior to enrollment
    - Risk factors for ARDS (sepsis, suspected of confirmed Covid-19, aspiration, trauma, pneumonia, other)
    - Ever smoker (>100 cigarettes in lifetime)?
        - If Yes, current smoker?
        - If ever smoker, estimate pack years [Pack years = (# packs per day) x (number of years smoked)]
        - If former smoker, when quit?
    - Evaluation of frequent alcohol intake (every day): Yes/No/Unknown
    - Basic assessment of prior functioning

- **<u>Daily from day 0 to day 7</u>**:
    - If receiving positive pressure ventilation: ventilator mode, set rate, actual rate, minute ventilation, tidal volume, FiO_2, PEEP, compliance of the respiratory system, airway resistance, ventilatory ratio, plateau,
    - PaO_2, $PaCO_2$, arterial pH, and SpO_2 on study days 1-7
    - Rescue procedures used:
        - Inhaled nitric oxide
        - Epoprostenol sodium
        - High-frequency ventilation
        - ECMO
        - Neuromuscular blockade after 48h from randomization
    - Pneumothorax (Yes / No)
    - Serum electrolytes and glucose
    - Administration of the following medication infusions (name)
        - Intravenous sedatives

- Neuromuscular blocking agent
- Intravenous or enteral corticosteroids (≥20 methylprednisolone equivalents)
- Antibiotic
- SARS-Cov2 (Covid-19) specific treatment, such as antiviral, anti-inflammatory (e.g., corticosteroids), immunomodulatory and/or other drug(s)...

- Sedation score: If RASS <-1 (or Riker<3, Ramsay >3), and sedation given, list reason given
- Was a sedation interruption performed (Yes / No)?
- Modified SOFA
- Serum liver function tests: AST, ALT, bilirubin, INR
- Central venous pressure (CVP) if available
- Hemodynamic measures: mean arterial pressure, maximal dose of infused norepinephrine or other vasopressor, serum lactate level, supraventricular tachycardia (SVT) or new onset atrial fibrillation
- KDIGO criteria for acute kidney injury[26]
- Confusion Assessment Method for the ICU (CAM-ICU)

- **At study entry (day 0) and on days 1, 2, 4, 6 and 14 or ICU discharge (whichever occurs first)**:
  - Plasma collection (Y/N; if N, reason)
  - Urine collection (Y/N; if N, reason)

- **At study entry (day 0) and on day 2**:
  - Total blood collection (Y/N; if N, reason)

- **Reference measurements**

The following data will provide the basis for assessing protocol compliance and safety as well as between-group differences in several efficacy variables. Data for each of the variables will be recorded on the days shown in the Time-Events schedule (Appendix B) or until death, or discharge from the ICU. Values will be derived from clinically available data.

a. Sedation dosing in the intervention arm
   i. Reason and duration of sevoflurane administration hold
b. Sedation dosing in the intervention arm

Name and of any sedative agent received intravenously in the first 7 days after randomization.

In both arms, the continuous use of cisatracurium, its duration and the reason for interruption will be recorded during the first 48 hours

- **On day 14 (or ICU discharge, whichever comes first)**:
  - Vital status
  - Modified SOFA
  - Plasma collection (Y/N; if N, reason)
  - Urine collection (Y/N; if N, reason)

- **On day 28 (or ICU discharge, whichever comes first)**:
  - Vital status
  - Modified SOFA

- **<u>On day 90 (3 months)</u>**:
  - Vital status
  - Disability:
    - Katz Activities of Daily Living (ADL)
  - Health-Related Quality of Life (including utilities):
    - Short Form-36 (SF-36)
  - Self-rated health: 1 standard item
  - Pain-interference: 1 standard item
  - Post-traumatic Stress-like Symptoms:
    - Post-Traumatic Stress Symptoms (PTSS-14)
    - Hospital Anxiety and Depression Scale (HADS)
  - Cognitive function: Alzheimer's Disease 8 (AD8)
  - Subsequent return to work, hospital and ED use, and location of residence

- **<u>On day 365 (12 months)</u>**:
  - Vital status
  - Disability:
    - Katz Activities of Daily Living (ADL)
  - Health-Related Quality of Life (including utilities):
    - Short Form-36 (SF-36)
  - Self-rated health: 1 standard item
  - Pain-interference: 1 standard item
  - Cognitive function: Alzheimer's Disease 8 (AD8)
  - Subsequent return to work, hospital and ED use, and location of residence
  - Post-traumatic Stress-like Symptoms:
    - Post-Traumatic Stress Symptoms (PTSS-14)
    - Hospital Anxiety and Depression Scale (HADS)

9. Statistics

9.1. Sample size estimation

In this study, the event of interest (primary outcome) is the number of days alive and off the ventilator at 28 days (VFD28, for ventilator-free days through day 28), and the competing event is death.
The following assumptions were made:
- The variability of days free from ventilation would follow the properties of recently published studies:
  - a pilot study from our group: median [interquartile range], 13 [1–20] and 5 [0–28] VFDs at day 28 in patients receiving inhaled sedation with sevoflurane and those receiving intravenous sedation, respectively[16].
  - a recent multicenter randomized controlled trial of mechanical ventilation targeting transpulmonary pressure (EPVent-2 trial) (22 [15-24] and 21 [16.5-24] VFDs at day 28 in the intervention (n=102) and control (n=98) groups, respectively)[33]
  - a recent multicenter randomized controlled trial of early neuromuscular

blockade (ROSE trial) (9.6 ± 10.4 and 9.9 ± 10.9 VFDs at day 28 in the intervention (n=501) and control (n=505) groups, respectively)[34]
- 28-day mortality would be around 30-35%, based on data from recent ARDS trials[33–35]

To detect a difference of 2 days free from ventilation at day 28 for a standard-deviation at 8[36,37], a two-sided type I error at 5%, and a statistical power greater than 80%, we have estimated that 340 patients by group would be necessary. We therefore propose to include 700 patients (350 by group).

An interim analysis will be performed after data from 350 patients (175 by group) have been obtained. This trial will stop for superiority of either active or control and is designed with symmetric group sequential flexible stopping boundaries (O'Brien-Fleming type I error of 0.0054), as described by Lan and DeMets (*Reference Lan, K. K. G. and DeMets, D. L. (1983), Discrete sequential boundaries for clinical trials. Biometrika 70, 659-663)*[38]. Recommendations for pausing or stopping the study will be made by the DMSC if it is found that the conduct of the trial compromises patient safety. The steering committee will be responsible to continue, hold or stop the study based on the DMSC recommendations.

Because findings from the SESAR trial may be invaluable to inform the potential impact of inhaled sedation with sevoflurane on the outcome of ARDS patients, which could be a major breakthrough to decrease the burden of the current Covid-19 pandemics, interim safety reports to the DMSC will be performed each time 40 patients (20 by group) are enrolled, according to previous results published by our team[16]. These interim reports will include blinded variables on randomization strata (inclusions by center, number of patients enrolled with Covid-19, PaO_2/FiO_2 <100 mmHg or shock at study entry), SAEs, and the rates and causes of death at day 28 in both groups.

The DMSC will be empowered to communicate these interim findings directly to sponsor (CHU Clermont-Ferrand) and public health authorities if the committee thinks the trial produces any results that may be of relevance to public health with regard to the current Covid-19 outbreak.

At the end of the Covid-19 pandemics, we will check if the results obtained in patients enrolled during the pandemics do not alter the initial assumptions for sample size estimation. Standardized mean (or median) differences and statistical power will then be estimated.

Data analysis: generalities

All analyses will be performed with Stata software (version 15, StataCorp, College Station, USA), R version 4.0.5 (R Foundation for Statistical Computing, Vienna, Austria), and MPlus Version 8.6 (Muthen & Muthen) before the breaking of the randomization code, according to International Conference on Harmonization-Good Clinical Practice guidelines[136,137]. The primary analysis will be by intention-to-treat (ITT). Then, we will perform per-protocol and subgroup analyses on the primary outcome and the most important secondary outcomes. The criteria for including patients in the ITT and in the per-protocol populations, respectively, are provided below.

Continuous variables will be presented as means and standard-deviations (as medians and quartiles, otherwise) and will be compared with the use of the unpaired t test or the Mann-Whitney U test when appropriate. The Shapiro-Wilk test will be used to assess normality, and the Fisher-Snedecor test to assess homoscedasticity. Categorical data will be presented as exact number and percentage and will be compared using unadjusted Chi-squared or Fisher's exact tests.

A two-sided P value of less than 0.05 will be considered for statistical significance of all analyses (except for interim analysis). Because of the potential for type 1 error due to multiple comparisons, findings for analyses of secondary endpoints will be interpreted as exploratory. As described by some authors[139], systematic correction of type I error will not be applied. As presented by Feise in 2002[140], "it must be careful to focus not only upon statistical significance (adjusted or not), but also upon the quality of the research within the study and the magnitude of difference".

9.1.1. Populations

- **Intention-to treat (ITT) population:** All randomized patients except those who withdraw their consent for the use of data.

- **Per-protocol populations:**

Per-protocol #1: All randomized patients except patients having one or more major protocol violations defined as:

1. Inhaled sevoflurane was not administered in patients randomly allocated to the intervention arm
OR
2. Inhaled sevoflurane was not administered during the whole duration of sedation (within a maximum of 7 days from randomization) in patients randomly allocated to the intervention arm
OR
3. Monitoring revealed that a tidal volume higher than 8 mL/kg PBW was applied
OR
4. Monitoring revealed that one or more inclusion or exclusion criteria were violated
OR
5. Patients withdrawn from the protocol because the patient would have withdrawn consent

Per-protocol #2: All randomized patients except patients having one or more major protocol violations defined as:

1. Inhaled sevoflurane was not administered in patients randomly allocated to the intervention arm
OR
2. Inhaled sevoflurane was not administered during the whole duration of

sedation (within a maximum of 7 days from randomization) in patients randomly allocated to the intervention arm

- **Subgroups:** subgroup analyses will be performed in

1. Patients with shock (defined as the need for intravenous vasopressor infusion to maintain arterial pressure) at randomization
2. Patients with pre-randomization PaO_2/FiO_2 <100 mmHg
3. Patients with hypoinflammatory vs hyperinflammatory subphenotypes[21,22] at randomization
4. Patients with higher vs. lower degrees of lung epithelial injury or of impaired AFC at randomization (as assessed by baseline plasma sRAGE; thresholds to be determined according to univariate analyses and clinical relevance)
5. Patients with higher vs. lower degrees of lung endothelial injury at randomization (as assessed by baseline plasma ANG-2; thresholds to be determined according to univariate analyses and clinical relevance)
6. Patients treated with lower (5-10 cmH_2O), moderate (11-15 cmH_2O) or higher (>15 cmH_2O) levels of PEEP during the first 3 days after enrollment
7. Pre-randomization presence vs. absence of suspected or Covid-19 infection

According to clinical relevance and to European Medicines (EMA) and Consolidated Standards of Reporting Trials (CONSORT) recommendations, subgroup analyses will be proposed after the study of subgroup × randomization group interaction in multivariable regression models.

9.1.2. Primary analysis

The primary outcome is days alive and off the ventilator at 28 days (VFD28, for ventilator-free days through day 28), thereby considering death as a competing event. The primary analysis will be based on a mixture of generalized gamma distributions to concatenate the overall frequency and distribution of the times. Intention-to-treat analysis will be considered for the primary outcome (*Checkley W et al. for the NIH Acute Respiratory Distress Syndrome Network Investigators. Inference for mutually exclusive competing events through a mixture of generalized gamma distributions. Epidemiology. 2010 Jul; 21(4): 557–565*)[138].

9.1.3. Secondary analysis

The analysis of <u>the primary outcome</u> will be complemented by multivariable analyses using generalized gamma distribution mixed models:

1) A first model including only the randomization-stratification variables (severe ARDS, COVID-19, and shock at enrollment) as covariates and center as random-effect (to measure between- and within-center variability);

2) A second model with both covariates from the first model (including center as random-effect) and covariates with anticipated relationships with the outcome and confirmed by univariate analyses. A generalized gamma distribution mixed model will be used with these covariates (criterion for entering variables tested in the model will be selected if P<0.10).

Results will be expressed as regression coefficients and 95% confidence

intervals. According to usual recommendations, the interactions between possible predictive factors will also be tested before considering unplanned subgroup analyses.

The <u>key secondary outcome</u> of day-90 survival will be estimated using Kaplan-Meier approach and will be compared using log-rank test in univariate analysis and marginal Cox proportional hazard regression in multivariable analysis. Adjusted analyses will be conducted using the same two models described above: the first model with only randomization-stratification variables as covariates and center as random-effect, and the second model with covariates with anticipated relationships with the outcome. Results will be expressed as hazard-ratios with 95% confidence intervals and proportional-hazard assumption will be verified using the Schoenfeld test and plotting residuals.

Categorical variables will be analyzed using chi-squared test or Fisher's exact test, as appropriate. For multivariable analysis, adjusted analyses will be performed with the use of random-effects robust Poisson generalized linear model (Stata commands glm, link=log and offset) to take into account within- and between-center variability with center as random-effect. Results will be expressed as Relative Risks and 95% confidence intervals. Adjusted analyses will be conducted using the same adjustment variables described for the first model of the primary endpoint analysis.

Continuous parameters outcomes will be compared between groups using Student's t-test or Mann-Whitney U test. Normality will be studied by the Shapiro-Wilk test and homoscedasticity using the Fisher-Snedecor test. Multivariable analyses will be performed with linear mixed models. Adjusted analyses will be conducted using the same adjustment variables described for the first model of the primary endpoint analysis. We will minimize the AIC criteria to determine the adequate relationship (logarithm, square root, linear, quadratic or cubic polynomial, or terciles or quartiles categorization). Results will be expressed as regression coefficients and 95% confidence intervals. For variables such as organ failure-free, ICU-free or hospital-free days, generalized linear models will initially use Poisson distribution or, alternatively, negative binomial distribution. When assumptions for these distributions are not met, we will analyze data using the nonparametric Van Elteren test, adjusted only for the center.

Longitudinal analysis of repeated data (such as changes in respiratory variables, in ventilatory settings, clinical and biological indices or scores over time) will be performed using mixed models in order to study fixed effects group, time-point evaluation, and their interaction taking into account between- and within-subject variability.

A learning curve analysis will be performed to evaluate if an improvement in terms of primary outcome is observed over time, in other words to assess whether the study results might be associated with some degree of "learning effect" (due to the specific training on inhaled sedation). A sensitivity analysis excluding the five first patients of each center will be conducted for the primary outcome.

An ancillary analysis will also be conducted to assess the presence of subphenotypes among patients with ARDS, based on distinct clinical, imaging[25,27,28], and/or biological[21,22] profiles (subphenotypes), and their differential therapeutic response to sevoflurane, if any, using multidimensional analyses as factorial analysis and latent-class analysis.

Further details will be provided in the predefined statistical analysis plan published before the randomization of the last participant.

9.2. Method for missing data

A sensitivity analysis will be performed and the nature of missing data will be studied (missing at random or not). The most appropriate approach to the imputation of missing data will be proposed accordingly.

Missing primary outcome data
We do not expect missing data for the primary outcome measure and only complete case-analysis will be performed.

Missing secondary outcomes data
Initially, a complete case-analysis will be performed.
If the frequency of missing data is >5%, an additional analysis will be performed using the multiple imputation method (STATA command mi). We will use outcomes and the most important baseline characteristics in the multiple imputation. The exact variables to be used to estimate the missing values will be outlined in the detailed statistical analysis plan; if multiple imputation is used, then the primary result of the trial will be based on the imputed data.

Supplementary analyses using imputed data will be also performed as described below:

Missing baseline data

- SAPS II: The score is based on values measured in the first 24 hours of hospital admission but we register SAPS II as a baseline score including values from the 24 hours prior to randomization, so that patients randomized in a few hours after hospital admission (emergency department, surgical ward or ICU) may have missing values.
- SOFA score: The score does not depend on when the patient is admitted to the hospital but we register SOFA at baseline including values from the first 24 hours prior to randomization. Patients randomized within a few hours after hospital admission may thus have missing values.

To put significant results into perspective, a sensitivity analysis will be conducted by defining a worst-case scenario in which patients with missing data do not react to the study treatment (whatever it may be). In addition, if multiple imputation is used, we will also provide a best-worst, worst-best case scenario as a sensitivity analysis to assess the potential impact of any pattern of missing data. In the best-worst case scenario, it is assumed that patients lost to follow-up in the experimental group have had a beneficial outcome and those with missing outcomes in the control group have had a harmful outcome. Conversely, in the worst-best case scenario, it is assumed that patients who were lost to follow-up in the experimental group have had a harmful outcome and that those lost to follow-up in the control group have had a beneficial outcome.

In this new version of the protocol elaborated in response to the Covid-19 outbreak, a large amount of missing data is anticipated for variables that can be considered less relevant, given the current context and the urgent priority to improve survival and decrease the need for invasive mechanical ventilation in patients with ARDS.

In case the enrollment of numerous patients with Covid-19-related ARDS is associated with too many missing data concerning secondary variables that could not be collected remotely from the patient's medical record, the investigators may consider increasing the total number of patients (without Covid-19) to be enrolled, after the end of the pandemics.

9.3. Statistical analysis

Bruno PEREIRA, PhD Biostatistics
Biostatistics unit, Department of Clinical Research and Innovation (DRCI)
CHU Clermont-Ferrand
58, Rue Montalembert, 63003 Clermont-Ferrand Cedex 1, France
Tel: +33 (0) 473754964 / bpereira@chu-clermontferrand.fr

The SESAR trial statistical analysis plan (and its successive versions) will be kept in the study records. The statistical analysis plan may be revised during the study, e.g. in order to take into account amendments to the protocol or any change in the conduct of the study that may have an impact on the statistical analysis plan as described in its current version.

10. Safety assessment – Management of adverse events

The investigator is responsible for reporting all adverse events.

10.1. Definitions

Adverse event: any untoward medical occurrence in a patient or clinical investigation subject administered a pharmaceutical product and which does not necessarily have to have a causal relationship with the research or with this treatment.

Adverse effect: any untoward response related to the research.

Serious adverse effects are sub-grouped as follows:

- ***Expected*** *serious adverse event*: any event that is described in the most recent version of the Investigator's Brochure, or in the Summary of Product Characteristics for marketed medicinal products, or in the instruction notice when the research concerns a medical device which is subject to CE marking. This definition also applies to an investigational medicinal product when administered for a same population outside the labeled indications.

- ***Unexpected*** *serious adverse event*: any event, the nature, severity or outcome of which is not consistent with the information in the most recent version of the Investigator's Brochure or the Summary of Product Characteristics for a marketed medicinal product or the information notice for a medical device.

Serious adverse event or effect: any undesirable event or effect which results in death, is life-threatening, requires in-patient hospitalization or prolongation of existing hospitalization, results in persistent or significant disability/incapacity, or is a congenital anomaly/birth defect.

The term "life-threatening" refers to an event in which the patient was at risk of death at the time of the event, independently of the consequences of corrective or palliative treatment.

The terms "*disability"* or "*incapacity"* refer to any clinically significant, temporary or persistent disability.

Death, regardless of the cause, including when it corresponds to progression of the disease under treatment, is considered a serious adverse event.

Other events which do not correspond to the above definitions can be considered "*potentially serious*", in particular certain laboratory anomalies. The investigator or sponsor's medical judgement can result in such events being reported in the same manner as "serious" events. It is necessary for study protocols to specify the characteristics of "potentially serious" events that are subject to reporting.

New information: event concerning the conduct of the research or the development of the medicinal product or related product, which is the object of the research, when said new information may jeopardize the safety of the research subjects. Examples include:

- an increase in the rate of occurrence of serious events;
- results of interim analyses, when relevant to the safety of the research subjects (notably a lack of efficacy);
- serious adverse events related to the clinical trial procedures;
- lack of efficacy with a medicinal product used to treat life-threatening disease;
- a major safety finding from animal studies that provides new information on the safety of the product;
- and generally, any new information that could lead to an unfavorable reassessment of the benefit/risk ratio of the research.

Any new information concerning the research (or the product used) which may jeopardize the safety of the research subjects will be subjected to appropriate urgent measures and prompt and timely notification by the Sponsor to the competent authority and the Ethics Committee.

10.2. Serious adverse event reporting

It is the investigator's obligation to report within 24 hours any serious adverse event occurring in any patient enrolled in a study:

- During the active phase of the study,
- In the weeks following cessation of treatment,
- Within the deadlines established for safety monitoring off treatment, before (wash-out or withdrawal phase) or after the active phase,
- After termination of the study, regardless of the time of the event, when no cause other than the research can reasonably be incriminated,

- On the “Serious adverse event report form”, indicating the date of onset, the severity, the causal relationship with the treatment (or product), and the follow-up/outcome.

The narrative describing the event should be completed and transmitted to the sponsor as soon as new, relevant information is received. Depending on the nature and seriousness of the event, copies of the patient’s anonymized medical record can be attached, as well as laboratory results.

When a serious adverse event persists at the end of the study, the investigator will continue to follow the patient until said event is considered resolved.

In accordance with the implementing decree 2006-477 of 26/04/2006 amending chapter 1 of title II of Book I of the first part of the Public Health Code relating to biomedical research, all suspected unexpected serious adverse effects must be reported by the sponsor to ANSM and to the Ethics Committee at first knowledge and no later than:

- 7 days after occurrence in case of death or a life-threatening event
- 15 days after occurrence for all other unexpected serious adverse events (SAE).

In the framework of this study, and because the study population is composed of critically ill patients admitted in intensive care units, deaths are expected as hospital mortality attributable to ARDS has been reported as high as 35-45% in most severe cases. Death within 28 days is a major component of the primary endpoint of the study and this event will be followed up very closely and data will be collected in the eCRF. Deaths related to the progression of the primary disease or to limitation of care will therefore not be systematically declared to the sponsor by the investigator(s), but these events will be collected in the eCRF.

Only unexpected events, such as <u>unexpected deaths</u> (i.e., not related to the progression of the primary disease or to limitation of care) or <u>severe hypercapnic acidosis that may be related to the study intervention</u> (pH <7.15, in the absence of metabolic acidosis and despite further tidal volume and/or respiratory rate increase, as described in the protocol) or <u>the development of malignant hyperthermia</u>, <u>propofol-related infusion syndrome</u> or <u>bronchopleural fistula persistent despite drainage</u> will be declared to the sponsor by the investigator(s).

The sponsor will decide upon the significance of the serious adverse events that it reports and the consequences thereof, in particular with respect to the conduct of the research.

The sponsor will also assess the causality of the adverse event with the research by means of a joint analysis with the Regional Pharmacovigilance Center.

The sponsor will maintain a detailed list of all adverse events reported by the investigator(s).

Once per year, or on request, the sponsor will submit an annual safety update report to ANSM and to the Ethics Committee containing all available safety information.

The sponsor will also provide the investigators with any information that may affect the safety of the research subjects.

10.3. Independent data monitoring and safety committee (DMSC)

The DMSC will be responsible for safeguarding the interests of trial participants, assessing the safety and efficacy of the interventions during the trial, and for monitoring the overall conduct of the clinical trial. The DMSC will provide recommendations about stopping or continuing the trial to the Steering Committee (SC) of the SESAR trial. To contribute to enhancing the integrity of the trial, the DMSC may also formulate recommendations relating to the selection/recruitment/retention of participants, their management, improving adherence to protocol-specified regimens and retention of participants, and the procedures for data management and quality control.

The DMSC will be advisory to the SC. The SC will be responsible for promptly reviewing the DMSC recommendations, to decide whether to continue or terminate the trial, and to determine whether amendments to the protocol or changes in trial conduct are required.

The DMSC is an independent multidisciplinary group consisting of two clinician-scientists (Prof. Todd W. Rice, Vanderbilt University Medical Center, Nashville, USA and Prof. Laurent Papazian, Assistance-Publique Hôpitaux de Marseille, France) and a biostatistician (Prof. Nicolas Molinari, INSERM, Montpellier, France) who ~~that~~, collectively, have experience in the management of ARDS ~~surgical~~ patients, have specific expertize in mechanical ventilation, sedation in general and inhaled sedation in particular, and in the conduct, monitoring and analysis of randomized clinical trials. The members of the DMSC have been chosen among experts without conflicts of interest that could be perceived as inferring with the study, in order to ensure their independence towards the study and the industry or other commercial entities; a declaration of conflicts of interest will be filled and made accessible upon request.

This independent DMSC will meet a first time at study initiation and then throughout the duration of the study at its own initiative or at the sponsor’s request, and at least for every 120 recruited patients. The DMSC’s opinion reports will be submitted in writing to the sponsor. The DMSC will remain blinded for the allocation during analysis; however, the observation of differences in serious adverse events between the two groups will allow, for safety reasons may the DMSC deem necessary, to unblind allocation groups.

The DMSC will be empowered to communicate interim findings directly to public health authorities if the committee thinks the additional interim analyses, as planned since version **7** (Covid-19) of the protocol, produce any results that may be of relevance to public health with regard to the current Covid-19 outbreak.

10.4. Termination of the study

No formal criteria will be set for stopping the study.

Nevertheless, an interim analysis will be performed after data from 300 patients (150 by group) have been obtained. This trial will stop for superiority of either active or control and is designed with symmetric group sequential flexible stopping boundaries as described by Lan and DeMets[38].

Recommendations for pausing or stopping the study will be made by the DMSC if it is found that the conduct of the trial compromises patient safety. The steering committee will be responsible to continue, hold or stop the study based on the DMSC

recommendations.

Because findings from the SESAR trial may be invaluable to inform the potential impact of inhaled sedation with sevoflurane on the outcome of ARDS patients, which could be a major breakthrough to decrease the burden of the current Covid-19 pandemics, interim safety reports to the DMSC will be performed each time 40 patients (20 by group) are enrolled. The DMSC will be empowered to communicate these interim findings directly to sponsor (CHU Clermont-Ferrand) and public health authorities if the committee thinks the trial produces any results that may be of relevance to public health with regard to the current Covid-19 outbreak.

10.5. Follow-up of patients presenting an adverse event

Patients with persistent adverse events will continue to be followed-up until the event is considered resolved or stabilized. Patients who had non-serious adverse events will be followed-up until the final study visit.

11. Right of access to source document and data

11.1. Access to data

The sponsor is responsible for obtaining the agreement of all parties involved in the research in order to guarantee direct access to all study sites, source data, source documents and reports for purposes of the sponsor’s quality control and audit.

The investigators will provide access to the documents and individual data that are strictly necessary for purposes of monitoring, quality control and audit of the biomedical research, to the persons authorized to consult said documents pursuant to the legislative and regulatory provisions in force (articles L.1121-3 and R.5121-13 Public Health Code).

11.2. Source data

Source documents, defined as any original document or object which proves the existence or accuracy of data or information recorded during the clinical study, will be stored for a period of 15 years by the investigator or by the hospital in the case of a hospital medical record.

11.3. Data confidentiality

Subject to the provisions relating to the confidentiality of data to which persons in charge of quality control of biomedical research have access (article L.1121-3 Public Health Code), and subject to the provisions relating to the confidentiality of information as concerns in particular the nature of the products being studied, the trials, the

persons undergoing the research and the results obtained (article R.5121-13 Public Health Code), persons having direct access shall take all necessary precautions to ensure the confidentiality of the information relating to the products being studied, the trials, the persons undergoing the research and notably their identity, and the results obtained.

These persons, as well as the investigators themselves, are bound by professional secrecy (in accordance with the conditions laid down in articles 226-13 and 226-14 of the penal code).

During the biomedical research or upon its completion, the data collected on the research subjects and transmitted to the sponsor by the investigators (or any other specialized study staff) shall be rendered anonymous.

In no case shall the names or addresses of the persons undergoing the research appear.

Anonymity of the subjects will be guaranteed by the creation of a subject identifying number.

The sponsor will ensure that each research subject has given his written consent allowing access his personal data, which is strictly necessary for quality control of the research.

11.4. Registration in the national file of biomedical research subjects

Not applicable.

12. Quality control and assurance

12.1. Engagement of the investigators and the sponsor of the study

The investigator undertakes to conduct the study in compliance with public health law 2004-806 of 9 August 2004 relating to biomedical research, the implementing decree 2006-477 of 26/04/2006 amending chapter 1 of title II of book 1 of the first part of the Public Health Code relating to biomedical research, and with the bylaws in force.

The study will also be conducted in compliance with Good Clinical Practices for biomedical research on medicinal products for human use, as laid down in article L.1121-3 Public Health Code and the decree of 24 November 2006.

The investigator also undertakes to comply with the Declaration of Helsinki of the World Medical Assembly (Tokyo 2004, revision).

12.2. Quality assurance

Clinical Research Associates (CRAs) designated by the sponsor will ensure the proper conduct of the study, the collection of data generated in writing, and their documentation, recording and reporting, as per the Standard Operating Procedures in effect at the CHU Clermont-Ferrand and in compliance with Good Clinical Practices and legislative and regulatory provisions in force.

12.3. Quality control

The investigator guarantees the authenticity of the data collected during the study and accepts the legal provisions authorizing the study sponsor to implement quality control.

The coordinating investigator and associated investigators therefore agree to make themselves available during Quality Control visits by the Clinical Research Associate that will be scheduled at regular intervals. The following items will be examined at each visit:

- Informed consent
- Compliance with the study protocol and procedures
- Quality of data recorded in the case report forms: accuracy, missing data, coherence with source documents (medical records, appointment calendars, original copies of laboratory results, etc.)
- Management of any study products

To facilitate the logistics of Quality Control visits, the sponsor might rely on the use of remote solutions and/or outsourcing of data monitoring in accordance with current regulatory, including recommendations from the CNIL on remote Quality Control of COVID-19 clinical trials published in April 2021 https://www.cnil.fr/sites/default/files/atoms/files/recommandations_provisoires_-_controle_qualite_a_distance_des_essais_cliniques_pendant_la_crise_sanitaire_liee_a_la_covid-19.pdf). When appropriate, patients will be informed about such a remote data monitoring, in accordance with the CNIL recommendations.

12.4. Case report form

At each participating center, data will be collected and entered into a dedicated, password-protected, SSL-encrypted electronic web-based case report form (eCRF) by trial or clinical trained personal, blinded to the allocation group, under the supervision of the trial site investigators.

Some **changes to data collection** (section 8) have been made in response to the current Covid-19 pandemics:

- Data will be collected remotely from the (electronic) patient health record by clinical research associates at each center, so that:
  - 1) **the risk for intensive care providers to be contaminated with the virus is minimized**, as no data will be collected at patient bedside
  - 2) **no additional work overload is needed from intensive care providers**, as their staffing may be very challenged in this threatening situation.
- **Relevant information on Covid-19**, such as specific interventions that may be delivered in these patients (such as antiviral, anti-inflammatory (e.g., corticosteroids), immunomodulatory and/or other drug(s)...) will be collected.

This strategy will allow the rigorous collection of most data relevant to major patient outcomes (survival, duration of mechanical ventilation, indices of lung function, and safety data) while ensuring the safety of both intensive care providers and clinical research associates, and preventing interaction to the priority care patients will receive in participating ICUs.

However, and as a result, a larger amount of missing data can be expected for some variables that may be considered less relevant to collect from the (electronic) patient health record in the current pandemic context due to the surge in patients with Covid-19.

13. Ethical considerations

13.1. Ethics Committee

The study protocol, patient information notice and consent form will be submitted to the designated *Comité de Protection des Personnes* (CPP).

Notification of a favorable opinion from the Ethics Committee will be transmitted to the sponsor and to ANSM. The sponsor of the study will send an authorization request to ANSM prior to study start.

13.2. Information for patients and written informed consent form

Because patients with ARDS are very likely to receive deep sedation and invasive mechanical ventilation (or to need emergent tracheal intubation and invasive mechanical ventilation if not already the case), they are very likely to lack capacity to provide informed consent when eligible to enrolment into the study and the study protocol provides for a waiver of informed consent from the patient. In addition, because in emergency situations, sedation and ventilation must be initiated as early as possible, the study protocol implies a short enrollment time window of 24 hours since ARDS Berlin criteria are met. Therefore, the consent from the patient's next of kin will be sought actively during the 24-hour enrollment time window. In case the patient's next of kin cannot be reached during this time window, the investigator will decide to include the patient in the study using an emergent consent procedure; no consent from the patient's next of kin will be required in this very specific case, but the investigator will inform the patient's next-of kin of his/her decision to include the patient in the study whenever possible.

Deferred informed consent will be obtained as soon as possible from participants for potential continuation of the research.

All information appears in an information notice and consent form given to the patient. Written informed consent will be obtained by the investigator.

These documents will be approved by the competent Ethics Committee.

Two original copies will be co-signed by both the investigator and the patient. The second copy is to be kept in the patient's medical record.

13.3. Protocol amendments

Protocol amendments must be qualified as substantial or non-substantial. According to their nature, they will be the object of a new Ethics Committee opinion and/or authorization from the competent authority.

14. Data processing and storage of study documents

14.1. Data entry and processing

At each participating center, data will be collected and entered into the electronic web-based case report form (eCRF) by trial or clinical trained personal (clinical research associate), blinded to the allocation group, under the supervision of the trial site investigators.

Data analysis will be carried out at the Biostatistical Unit, Department of Clinical Research and Innovation (DRCI), CHU Clermont-Ferrand.

14.2. CNIL

This study enters within the scope of “Reference Methodology” in application of the provisions of the law of 6 August 2004 relating to the protection of natural persons with regard to the processing of personal data and amending the law of 6 January 1978 relating to computer processing, data files and civil liberties. This change was approved by decision of 5 January 2006. The CHU Clermont-Ferrand, which sponsored the study, has signed a commitment to comply with this “Reference Methodology” on March, 15, 2007.

14.3. Data retention and archiving

The following documents will be archived under the study name in the Department of Perioperative Medicine (Prof. Jean-Etienne Bazin, Head of the Department of Perioperative Medicine, CHU Clermont-Ferrand, Clermont-Ferrand, France) until the end of the period of practical usefulness (60 months, including inclusion of patients and data analysis).

These documents are:

- Protocol and appendices, and any amendments,
- Signed, original information notices and consent forms,
- Individual data (authenticated copies of raw data),
- Follow-up documents
- Statistical analyses
- Final study report

At the end of the period of practical usefulness, all documents to be archived, such as defined in procedure PG.06.005 “Management of documentation relating to protocols” of Clermont-Ferrand Hospital will be transferred to the central archives and placed under the sponsor’s responsibility for a period of 15 years after study completion, in accordance with institutional practices. These documents cannot be moved or destroyed without the sponsor’s permission. After the 15 years are up, the sponsor will be consulted for destruction. All the data as well as all documents and reports may be subject to audit or inspection.

15. Funding and insurance

A detailed budget for the SESAR trial is given on the INNOVARC platform. Approximate level of total funding required is 766 k€.

AnaConDa-S devices will be graciously provided to all participating centers by Sedana Medical (Danderyd, Sweden). Sedana Medical has no influence on the study design, conduct, and analysis.

In accordance with regulatory provisions, the CHU Clermont-Ferrand, in its capacity as sponsor, has taken out civil liability insurance covering any damages resulting from the research with the *Société Hospitalière d'Assurances Mutuelles* (SHAM).

It should be noted that non-observance of the legal conditions of the research (absence of Ethics Committee opinion, absence of ANSM authorization, non-consent of subjects, and continuation of a suspended or prohibited study) shall render this coverage void.

16. Communication - Rules for publication

All trial sites including patients will be acknowledged, and all investigators at these sites will appear with their names under the 'SESAR investigators' in an Appendix to the final manuscript. The Steering Committee will grant authorship depending on personal involvement according to the Vancouver definitions. The listing of authors will be as follows: M Jabaudon (principal investigator) will be responsible for the writing of the manuscript and the first author, the second author will be R Blondonnet, and the next authors will be trial site investigators dependent on the number of included patients per site. B Pereira will appear as the second to last author, JM Constantin will appear as the last author, and then 'for the SESAR study group'.

Funding sources will have no influence on data handling or analysis or writing of the manuscript. Side studies will be allowed if supported by the Steering committee.

The study will be registered and declared at ClinicalTrials.gov.

17. Feasibility

We have estimated that 700 patients (350 by group) will be necessary for a two-sided type I error at 5% and a statistical power greater than 80%.

To ensure the feasibility of the study, the following were taken into consideration:

- Patients will be recruited from 37 centers during a 3-year period. Each center has 0.55 patient per month to include (holidays excluded) to finish inclusion within 3 years (see table below). Given the incidence of ARDS and available

data from these centers, a study termination by that deadline is highly feasible.
- Inclusion/non-inclusion criteria are consistent with ICU patient characteristics in the setting of ARDS. A mean number of patients meeting non-inclusion criteria (including refusal to participate) of no more than 15% is anticipated (worse scenario).
- Decisions about most aspects of patient care will be performed according to the expertize and routine clinical practice at each center. Little differences with standard practice set the stage for good adherence to the study protocol.
- A steering committee will insure the supervision of the trial. Regular meetings will be planned to evaluate the progress of the trial and adherence to the protocol.
- Dedicated clinical research associates will be made available at each center for follow-up and data registration.
- Most study endpoints are commonly evaluated in the ICU setting.

Expected number of patients eligible in each center during the study period

Last name	First name	Town	Country	Expected recruitment/month	Total (*)
Blondonnet	Raïko	Clermont-Ferrand	France	0.53	19
Roquilly	Antoine	Nantes	France	0.53	19
Jaber	Samir	Montpellier	France	0.53	19
Lemiale	Virginie	Paris	France	0.53	19
Ichai	Carole	Nice	France	0.53	19
Gainnier	Marc	Marseille	France	0.53	19
Velly	Lionel	Marseille	France	0.53	19
Bulyez	Stéphanie	Nîmes	France	0.53	19
Lasocki	Sigismond	Angers	France	0.53	19
Constantin	Jean-Michel	Paris	France	0.53	19
Quenot	Jean-Pierre	Dijon	France	0.53	19
Lebouvier	Thomas	Rennes	France	0.53	19
Legay	François	Saint-Brieuc	France	0.53	19
Thille	Arnaud	Poitiers	France	0.53	19
Lautrette	Alexandre	Clermont-Ferrand	France	0.53	19
Pottecher	Julien	Strasbourg	France	0.53	19
Garnier	Marc	Paris	France	0.53	19
Vinsonneau	Christophe	Béthune	France	0.53	19
Bertrand	Pierre-Marie	Cannes	France	0.53	19
Monchi	Mehran	Melun	France	0.53	19
Cousson	Joël	Reims	France	0.53	19
Maizel	Julien	Amiens	France	0.53	19
L'Her	Erwan	Brest	France	0.53	19
Bouhemad	Belaïd	Dijon	France	0.53	19
Jung	Boris	Montpellier	France	0.53	19
Dahyot-Fizelier	Claire	Poitiers	France	0.53	19
Amathieu	Roland	Paris	France	0.53	19
Varillon	Caroline	Dunkerque	France	0.53	19
Durand	Arthur	Lille	France	0.53	19
Lorber	Julien	Belfort	France	0.53	19
Badie	Julio	Saint-Nazaire	France	0.53	19
Lambiotte	Fabien	Valenciennes	France	0.53	19
Brégeaud	Delphine	Saintes	France	0.53	19
Berrouba	Aziz	Martigues	France	0.53	19
Conia	Alexandre	Chartres	France	0.50	18
Ferrandière	Martine	Tours	France	0.50	18
Thouy	François	Clermont-Ferrand	France	0.50	18
					700

** holidays excluded*

18. References

1. ARDS Definition Task Force *et al.* Acute respiratory distress syndrome: the Berlin Definition. *JAMA* **307**, 2526–2533 (2012).
2. Bellani, G. *et al.* Epidemiology, Patterns of Care, and Mortality for Patients With Acute Respiratory Distress Syndrome in Intensive Care Units in 50 Countries. *JAMA* **315**, 788–800 (2016).
3. Ware, L. B. & Matthay, M. A. The acute respiratory distress syndrome. *N. Engl. J. Med.* **342**, 1334–1349 (2000).
4. Ware, L. B. & Matthay, M. A. Alveolar fluid clearance is impaired in the majority of patients with acute lung injury and the acute respiratory distress syndrome. *Am. J. Respir. Crit. Care Med.* **163**, 1376–1383 (2001).
5. Acute Respiratory Distress Syndrome Network *et al.* Ventilation with lower tidal volumes as compared with traditional tidal volumes for acute lung injury and the acute respiratory distress syndrome. The Acute Respiratory Distress Syndrome Network. *N. Engl. J. Med.* **342**, 1301–1308 (2000).
6. Frank, A. J. & Thompson, B. T. Pharmacological treatments for acute respiratory distress syndrome. *Curr. Opin. Crit. Care* **16**, 62–68 (2010).
7. Ferrando, C. *et al.* Sevoflurane, but not propofol, reduces the lung inflammatory response and improves oxygenation in an acute respiratory distress syndrome model: A randomised laboratory study. *Eur. J. Anaesthesiol.* **30**, 455–463 (2013).
8. Voigtsberger, S. *et al.* Sevoflurane ameliorates gas exchange and attenuates lung damage in experimental lipopolysaccharide-induced lung injury. *Anesthesiology* **111**, 1238–1248 (2009).
9. Schläpfer, M. *et al.* Sevoflurane reduces severity of acute lung injury possibly by impairing formation of alveolar oedema. *Clin. Exp. Immunol.* **168**, 125–134 (2012).
10. Suter, D. *et al.* The immunomodulatory effect of sevoflurane in endotoxin-injured alveolar epithelial cells. *Anesth. Analg.* **104**, 638–645 (2007).
11. Steurer, M. *et al.* The volatile anaesthetic sevoflurane attenuates lipopolysaccharide-induced injury in alveolar macrophages. *Clin. Exp. Immunol.* **155**, 224–230 (2009).
12. Jerath, A., Parotto, M., Wasowicz, M. & Ferguson, N. D. Volatile Anesthetics. Is a New Player Emerging in Critical Care Sedation? *Am. J. Respir. Crit. Care Med.* **193**, 1202–1212 (2016).
13. Mesnil, M. *et al.* Long-term sedation in intensive care unit: a randomized comparison between inhaled sevoflurane and intravenous propofol or midazolam. *Intensive Care Med.* **37**, 933–941 (2011).
14. Jerath, A. *et al.* Safety and Efficacy of Volatile Anesthetic Agents Compared With Standard Intravenous Midazolam/Propofol Sedation in Ventilated Critical Care Patients: A Meta-analysis and Systematic Review of Prospective Trials. *Anesth. Analg.* **124**, 1190–1199 (2017).
15. O'Gara, B. & Talmor, D. Lung protective properties of the volatile anesthetics. *Intensive Care Med.* **42**, 1487–1489 (2016).
16. Jabaudon, M. *et al.* Sevoflurane for Sedation in Acute Respiratory Distress Syndrome. A Randomized Controlled Pilot Study. *Am. J. Respir. Crit. Care Med.* **195**, 792–800 (2017).
17. Jabaudon, M. *et al.* Soluble Receptor for Advanced Glycation End-Products Predicts Impaired Alveolar Fluid Clearance in Acute Respiratory Distress Syndrome. *Am. J. Respir. Crit. Care Med.* **192**, 191–199 (2015).
18. Bourdeaux, D. *et al.* Simple assay of plasma sevoflurane and its metabolite hexafluoroisopropanol by headspace GC–MS. *Journal of Chromatography B* **878**, 45–50 (2010).
19. Chabanne, R. *et al.* Impact of the anesthetic conserving device on respiratory parameters and work of breathing in critically ill patients under light sedation with sevoflurane. *Anesthesiology* **121**, 808–816 (2014).
20. Perbet, S. *et al.* A pharmacokinetic study of 48-hour sevoflurane inhalation using a

disposable delivery system (AnaConDa®) in ICU patients. *Minerva Anestesiol.* **80**, 655–665 (2014).

21. Calfee, C. S. *et al.* Subphenotypes in acute respiratory distress syndrome: latent class analysis of data from two randomised controlled trials. *Lancet Respir Med* **2**, 611–620 (2014).
22. Famous, K. R. *et al.* Acute Respiratory Distress Syndrome Subphenotypes Respond Differently to Randomized Fluid Management Strategy. *Am. J. Respir. Crit. Care Med.* **195**, 331–338 (2017).
23. Prescott, H. C., Calfee, C. S., Thompson, B. T., Angus, D. C. & Liu, V. X. Toward Smarter Lumping and Smarter Splitting: Rethinking Strategies for Sepsis and Acute Respiratory Distress Syndrome Clinical Trial Design. *Am. J. Respir. Crit. Care Med.* **194**, 147–155 (2016).
24. Beitler, J. R. *et al.* Personalized medicine for ARDS: the 2035 research agenda. *Intensive Care Med.* **42**, 756–767 (2016).
25. Mrozek, S. *et al.* Elevated Plasma Levels of sRAGE Are Associated With Nonfocal CT-Based Lung Imaging in Patients With ARDS: A Prospective Multicenter Study. *Chest* **150**, 998–1007 (2016).
26. Khwaja, A. KDIGO clinical practice guidelines for acute kidney injury. *Nephron Clin. Pract.* **120**, c179–84 (2012).
27. Constantin, J.-M. *et al.* Lung morphology predicts response to recruitment maneuver in patients with acute respiratory distress syndrome. *Crit. Care Med.* **38**, 1108–1117 (2010).
28. Puybasset, L. *et al.* Regional distribution of gas and tissue in acute respiratory distress syndrome. III. Consequences for the effects of positive end-expiratory pressure. *Intensive Care Med.* **26**, 1215–1227 (2000).
29. Guérin, C. *et al.* Prone positioning in severe acute respiratory distress syndrome. *N. Engl. J. Med.* **368**, 2159–2168 (2013).
30. Constantin, J. M. Lung Imaging for Ventilatory Setting in ARDS (LIVE Study). *ClinicalTrials.gov* Available at: http://clinicaltrials.gov/ct2/show/NCT02149589. (Accessed: 31st October 2014)
31. Mercat, A. *et al.* Positive end-expiratory pressure setting in adults with acute lung injury and acute respiratory distress syndrome: a randomized controlled trial. *JAMA* **299**, 646–655 (2008).
32. Fan, E. *et al.* An Official American Thoracic Society/European Society of Intensive Care Medicine/Society of Critical Care Medicine Clinical Practice Guideline: Mechanical Ventilation in Adult Patients with Acute Respiratory Distress Syndrome. *Am. J. Respir. Crit. Care Med.* **195**, 1253–1263 (2017).
33. Beitler, J. R. *et al.* Effect of Titrating Positive End-Expiratory Pressure (PEEP) With an Esophageal Pressure-Guided Strategy vs an Empirical High PEEP-Fio2 Strategy on Death and Days Free From Mechanical Ventilation Among Patients With Acute Respiratory Distress Syndrome: A Randomized Clinical Trial. *JAMA* (2019). doi:10.1001/jama.2019.0555
34. National Heart, Lung, and Blood Institute PETAL Clinical Trials Network *et al.* Early Neuromuscular Blockade in the Acute Respiratory Distress Syndrome. *N. Engl. J. Med.* **380**, 1997–2008 (2019).
35. Jabaudon, M. *et al.* Rationale, study design and analysis plan of the lung imaging morphology for ventilator settings in acute respiratory distress syndrome study (LIVE study): Study protocol for a randomised controlled trial. *Anaesth Crit Care Pain Med* **36**, 301–306 (2017).
36. Wan, X., Wang, W., Liu, J. & Tong, T. Estimating the sample mean and standard deviation from the sample size, median, range and/or interquartile range. *BMC Med. Res. Methodol.* **14**, 135 (2014).
37. Hozo, S. P., Djulbegovic, B. & Hozo, I. Estimating the mean and variance from the median, range, and the size of a sample. *BMC Med. Res. Methodol.* **5**, 13 (2005).
38. K. K. Gordon Lan & DeMets, D. L. Discrete Sequential Boundaries for Clinical Trials. *Biometrika* **70**, 659–663 (1983).
39. Herridge, M. S. *et al.* Functional disability 5 years after acute respiratory distress syndrome. *N. Engl. J. Med.* **364**, 1293–1304 (2011).

40. Adhikari, N. K. J. *et al.* Self-reported depressive symptoms and memory complaints in survivors five years after ARDS. *Chest* **140**, 1484–1493 (2011).
41. Dowdy, D. W. *et al.* Quality of life after acute respiratory distress syndrome: a meta-analysis. *Intensive Care Med.* **32**, 1115–1124 (2006).
42. Hopkins, R. O. *et al.* Two-year cognitive, emotional, and quality-of-life outcomes in acute respiratory distress syndrome. *Am. J. Respir. Crit. Care Med.* **171**, 340–347 (2005).
43. Marti, J. *et al.* One-year resource utilisation, costs and quality of life in patients with acute respiratory distress syndrome (ARDS): secondary analysis of a randomised controlled trial. *J. Intensive Care Med.* **4**, 56 (2016).
44. Herridge, M. S. *et al.* Recovery and outcomes after the acute respiratory distress syndrome (ARDS) in patients and their family caregivers. *Intensive Care Med.* **42**, 725–738 (2016).
45. Pfoh, E. R. *et al.* Physical declines occurring after hospital discharge in ARDS survivors: a 5-year longitudinal study. *Intensive Care Med.* **42**, 1557–1566 (2016).
46. Mehta, S. & Povoa, P. Long-term physical morbidity in ARDS survivors. *Intensive Care Med.* **43**, 101–103 (2017).
47. Ashbaugh, D. G., Bigelow, D. B., Petty, T. L. & Levine, B. E. Acute respiratory distress in adults. *Lancet* **2**, 319–323 (1967).
48. Papazian, L. *et al.* Neuromuscular blockers in early acute respiratory distress syndrome. *N. Engl. J. Med.* **363**, 1107–1116 (2010).
49. Huang, D. T. *et al.* Design and Rationale of the Reevaluation of Systemic Early Neuromuscular Blockade Trial for Acute Respiratory Distress Syndrome. *Ann. Am. Thorac. Soc.* **14**, 124–133 (2017).
50. Bisbal, M. *et al.* [Efficacy, safety and cost of sedation with sevoflurane in intensive care unit]. *Ann. Fr. Anesth. Reanim.* **30**, 335–341 (2011).
51. Ford, W. W. Ether inhalers in early use. *N. Engl. J. Med.* **234**, 713–726 (1946).
52. Barr, J. *et al.* Clinical practice guidelines for the management of pain, agitation, and delirium in adult patients in the intensive care unit. *Crit. Care Med.* **41**, 263–306 (2013).
53. Barr, J. Propofol: a new drug for sedation in the intensive care unit. *Int. Anesthesiol. Clin.* **33**, 131–154 (1995).
54. Sleigh, J., Harvey, M., Voss, L. & Denny, B. Ketamine – More mechanisms of action than just NMDA blockade. *Trends in Anaesthesia and Critical Care* **4**, 76–81 (2014/6).
55. Jackson, D. L., Proudfoot, C. W., Cann, K. F. & Walsh, T. S. The incidence of sub-optimal sedation in the ICU: a systematic review. *Crit. Care* **13**, R204 (2009).
56. Girard, T. D. *et al.* Efficacy and safety of a paired sedation and ventilator weaning protocol for mechanically ventilated patients in intensive care (Awakening and Breathing Controlled trial): a randomised controlled trial. *Lancet* **371**, 126–134 (2008).
57. Wade, D. M. *et al.* Investigating risk factors for psychological morbidity three months after intensive care: a prospective cohort study. *Crit. Care* **16**, R192 (2012).
58. Girard, T. D. *et al.* Risk factors for post-traumatic stress disorder symptoms following critical illness requiring mechanical ventilation: a prospective cohort study. *Crit. Care* **11**, R28 (2007).
59. Long, A. C., Kross, E. K., Davydow, D. S. & Curtis, J. R. Posttraumatic stress disorder among survivors of critical illness: creation of a conceptual model addressing identification, prevention, and management. *Intensive Care Med.* **40**, 820–829 (2014).
60. Roberts, R. J. *et al.* Incidence of propofol-related infusion syndrome in critically ill adults: a prospective, multicenter study. *Crit. Care* **13**, R169 (2009).
61. Jakob, S. M. *et al.* Dexmedetomidine vs midazolam or propofol for sedation during prolonged mechanical ventilation: two randomized controlled trials. *JAMA* **307**, 1151–1160 (2012).
62. Pandharipande, P. P. *et al.* Effect of sedation with dexmedetomidine vs lorazepam on acute brain dysfunction in mechanically ventilated patients: the MENDS randomized controlled trial. *JAMA* **298**, 2644–2653 (2007).
63. Preckel, B. & Bolten, J. Pharmacology of modern volatile anaesthetics. *Best Pract. Res. Clin. Anaesthesiol.* **19**, 331–348 (2005).
64. Campagna, J. A., Miller, K. W. & Forman, S. A. Mechanisms of actions of inhaled anesthetics. *N. Engl. J. Med.* **348**, 2110–2124 (2003).

65. Breheny, F. X. & Kendall, P. A. Use of isoflurane for sedation in intensive care. *Crit. Care Med.* **20**, 1062–1064 (1992).
66. Soukup, J. *et al.* State of the art: sedation concepts with volatile anesthetics in critically Ill patients. *J. Crit. Care* **24**, 535–544 (2009).
67. Sackey, P. V., Martling, C.-R. & Radell, P. J. Three cases of PICU sedation with isoflurane delivered by the 'AnaConDa'. *Paediatr. Anaesth.* **15**, 879–885 (2005).
68. Redaelli, S. *et al.* Prolonged sedation in ARDS patients with inhaled anesthetics: our experience. *Crit. Care* **17**, P386 (2013).
69. Woebkenberg, M. L. & Doemeny, L. J. NIOSH's criteria for a recommended standard--occupational exposure to waste anesthetic gases and vapors. *Am. Ind. Hyg. Assoc. J.* **39**, 598–600 (1978).
70. Pickworth, T., Jerath, A., DeVine, R., Kherani, N. & Wąsowicz, M. The scavenging of volatile anesthetic agents in the cardiovascular intensive care unit environment: a technical report. *Can. J. Anaesth.* **60**, 38–43 (2013).
71. Migliari, M. *et al.* Short-term evaluation of sedation with sevoflurane administered by the anesthetic conserving device in critically ill patients. *Intensive Care Med.* **35**, 1240–1246 (2009).
72. Sackey, P. V., Martling, C.-R., Nise, G. & Radell, P. J. Ambient isoflurane pollution and isoflurane consumption during intensive care unit sedation with the Anesthetic Conserving Device. *Crit. Care Med.* **33**, 585–590 (2005).
73. L'her, E. *et al.* Feasibility and potential cost/benefit of routine isoflurane sedation using an anesthetic-conserving device: a prospective observational study. *Respir. Care* **53**, 1295–1303 (2008).
74. Meiser, A. *et al.* Desflurane compared with propofol for postoperative sedation in the intensive care unit. *Br. J. Anaesth.* **90**, 273–280 (2003).
75. Röhm, K. D. *et al.* Short-term sevoflurane sedation using the Anaesthetic Conserving Device after cardiothoracic surgery. *Intensive Care Med.* **34**, 1683–1689 (2008).
76. Weiser, T. G. *et al.* An estimation of the global volume of surgery: a modelling strategy based on available data. *Lancet* **372**, 139–144 (2008).
77. Khuri, S. F. *et al.* Determinants of long-term survival after major surgery and the adverse effect of postoperative complications. *Ann. Surg.* **242**, 326–41; discussion 341–3 (2005).
78. De Conno, E. *et al.* Anesthetic-induced improvement of the inflammatory response to one-lung ventilation. *Anesthesiology* **110**, 1316–1326 (2009).
79. Uhlig, C. *et al.* Effects of Volatile Anesthetics on Mortality and Postoperative Pulmonary and Other Complications in Patients Undergoing Surgery: A Systematic Review and Meta-analysis. *Anesthesiology* (2016). doi:10.1097/ALN.0000000000001120
80. Fortis, S. *et al.* Effects of anesthetic regimes on inflammatory responses in a rat model of acute lung injury. *Intensive Care Med.* **38**, 1548–1555 (2012).
81. Watanabe, K. *et al.* Sevoflurane suppresses tumour necrosis factor-α-induced inflammatory responses in small airway epithelial cells after anoxia/reoxygenation. *Br. J. Anaesth.* **110**, 637–645 (2013).
82. Yao, Y., Li, L., Li, L., Gao, C. & Shi, C. Sevoflurane postconditioning protects chronically-infarcted rat hearts against ischemia-reperfusion injury by activation of pro-survival kinases and inhibition of mitochondrial permeability transition pore opening upon reperfusion. *Biol. Pharm. Bull.* **32**, 1854–1861 (2009).
83. Chung, I. S. *et al.* Reactive oxygen species by isoflurane mediates inhibition of nuclear factor κB activation in lipopolysaccharide-induced acute inflammation of the lung. *Anesth. Analg.* **116**, 327–335 (2013).
84. Englert, J. A. *et al.* Isoflurane Ameliorates Acute Lung Injury by Preserving Epithelial Tight Junction Integrity. *Anesthesiology* **123**, 377–388 (2015).
85. Bellgardt, M. *et al.* Survival after long-term isoflurane sedation as opposed to intravenous sedation in critically ill surgical patients: Retrospective analysis. *Eur. J. Anaesthesiol.* **33**, 6–13 (2016).
86. Soukup, J. *et al.* Efficiency and safety of inhalative sedation with sevoflurane in comparison to an intravenous sedation concept with propofol in intensive care patients: study protocol for a randomized controlled trial. *Trials* **13**, 135 (2012).
87. Schuster, F., Moegele, S., Johannsen, S. & Roewer, N. Malignant hyperthermia in the

intensive care setting. *Crit. Care* **18**, 411 (2014).
88. Bergeron, N., Dubois, M. J., Dumont, M., Dial, S. & Skrobik, Y. Intensive Care Delirium Screening Checklist: evaluation of a new screening tool. *Intensive Care Med.* **27**, 859–864 (2001).
89. Hellström, J., Öwall, A. & Sackey, P. V. Wake-up times following sedation with sevoflurane versus propofol after cardiac surgery. *Scand. Cardiovasc. J.* **46**, 262–268 (2012).
90. Sackey, P. V., Martling, C.-R., Carlswärd, C., Sundin, O. & Radell, P. J. Short- and long-term follow-up of intensive care unit patients after sedation with isoflurane and midazolam--a pilot study. *Crit. Care Med.* **36**, 801–806 (2008).
91. Cousins, M. J. & Mazze, R. I. Methoxyflurane nephrotoxicity. A study of dose response in man. *JAMA* **225**, 1611–1616 (1973).
92. Röhm, K. D. *et al.* Renal integrity in sevoflurane sedation in the intensive care unit with the anesthetic-conserving device: a comparison with intravenous propofol sedation. *Anesth. Analg.* **108**, 1848–1854 (2009).
93. Calfee, C. S. *et al.* Plasma angiopoietin-2 in clinical acute lung injury: prognostic and pathogenetic significance. *Crit. Care Med.* **40**, 1731–1737 (2012).
94. Calfee, C. S. *et al.* Distinct molecular phenotypes of direct vs indirect ARDS in single-center and multicenter studies. *Chest* **147**, 1539–1548 (2015).
95. Uchida, T. *et al.* Receptor for advanced glycation end-products is a marker of type I cell injury in acute lung injury. *Am. J. Respir. Crit. Care Med.* **173**, 1008–1015 (2006).
96. Vijayan, A. *et al.* Clinical Use of the Urine Biomarker [TIMP-2] × [IGFBP7] for Acute Kidney Injury Risk Assessment. *Am. J. Kidney Dis.* **68**, 19–28 (2016).
97. McNeil, J. B. *et al.* Novel Method for Noninvasive Sampling of the Distal Airspace in Acute Respiratory Distress Syndrome. *Am. J. Respir. Crit. Care Med.* **197**, 1027–1035 (2018).
98. Brochard, L. Intrinsic (or auto-) PEEP during controlled mechanical ventilation. *Intensive Care Med.* **28**, 1376–1378 (2002).
99. Sinha, P. *et al.* Physiologic Analysis and Clinical Performance of the Ventilatory Ratio in Acute Respiratory Distress Syndrome. *Am. J. Respir. Crit. Care Med.* **199**, 333–341 (2019).
100. De Jonghe, B. *et al.* Paresis acquired in the intensive care unit: a prospective multicenter study. *JAMA* **288**, 2859–2867 (2002).
101. Ciesla, N. *et al.* Manual muscle testing: a method of measuring extremity muscle strength applied to critically ill patients. *J. Vis. Exp.* (2011). doi:10.3791/2632
102. Hodgson, C. *et al.* Feasibility and inter-rater reliability of the ICU Mobility Scale. *Heart Lung* **43**, 19–24 (2014).
103. Ely, E. W. *et al.* Delirium in mechanically ventilated patients: validity and reliability of the confusion assessment method for the intensive care unit (CAM-ICU). *JAMA* **286**, 2703–2710 (2001).
104. Ely, E. W. *et al.* Evaluation of delirium in critically ill patients: validation of the Confusion Assessment Method for the Intensive Care Unit (CAM-ICU). *Crit. Care Med.* **29**, 1370–1379 (2001).
105. Page, V. J. *et al.* Evaluation of early administration of simvastatin in the prevention and treatment of delirium in critically ill patients undergoing mechanical ventilation (MoDUS): a randomised, double-blind, placebo-controlled trial. *Lancet Respir Med* **5**, 727–737 (2017).
106. Katz, S., Ford, A. B., Moskowitz, R. W., Jackson, B. A. & Jaffe, M. W. Studies of Illness in the Aged: The Index of ADL: A Standardized Measure of Biological and Psychosocial Function. *JAMA* **185**, 914–919 (1963).
107. Granja, C. *et al.* Understanding posttraumatic stress disorder-related symptoms after critical care: the early illness amnesia hypothesis. *Crit. Care Med.* **36**, 2801–2809 (2008).
108. Twigg, E., Humphris, G., Jones, C., Bramwell, R. & Griffiths, R. D. Use of a screening questionnaire for post-traumatic stress disorder (PTSD) on a sample of UK ICU patients. *Acta Anaesthesiol. Scand.* **52**, 202–208 (2008).
109. Zigmond, A. S. & Snaith, R. P. The hospital anxiety and depression scale. *Acta Psychiatr. Scand.* **67**, 361–370 (1983).
110. Pochard, F. *et al.* Symptoms of anxiety and depression in family members of intensive

care unit patients: ethical hypothesis regarding decision-making capacity. *Crit. Care Med.* **29**, 1893–1897 (2001).
111. Galvin, J. E. *et al.* The AD8: a brief informant interview to detect dementia. *Neurology* **65**, 559–564 (2005).
112. Calfee, C. S. *et al.* Acute respiratory distress syndrome subphenotypes and differential response to simvastatin: secondary analysis of a randomised controlled trial. *Lancet Respir Med* **6**, 691–698 (2018).
113. Sinha, P. *et al.* Latent class analysis of ARDS subphenotypes: a secondary analysis of the statins for acutely injured lungs from sepsis (SAILS) study. *Intensive Care Med.* (2018). doi:10.1007/s00134-018-5378-3
114. Strange, C., Vaughan, L., Franklin, C. & Johnson, J. Comparison of train-of-four and best clinical assessment during continuous paralysis. *Am. J. Respir. Crit. Care Med.* **156**, 1556–1561 (1997).
115. Rudis, M. I. *et al.* A prospective, randomized, controlled evaluation of peripheral nerve stimulation versus standard clinical dosing of neuromuscular blocking agents in critically ill patients. *Crit. Care Med.* **25**, 575–583 (1997).
116. Baumann, M. H. *et al.* A prospective randomized comparison of train-of-four monitoring and clinical assessment during continuous ICU cisatracurium paralysis. *Chest* **126**, 1267–1273 (2004).
117. Brower, R. G. *et al.* Higher versus lower positive end-expiratory pressures in patients with the acute respiratory distress syndrome. *N. Engl. J. Med.* **351**, 327–336 (2004).
118. Grissom, C. K. F. *et al.* Fluid Management With a Simplified Conservative Protocol for the Acute Respiratory Distress Syndrome*. *Crit. Care Med.* **43**, 288–295 (2015).
119. Charlson, M. E., Pompei, P., Ales, K. L. & MacKenzie, C. R. A new method of classifying prognostic comorbidity in longitudinal studies: development and validation. *J. Chronic Dis.* **40**, 373–383 (1987).
120. McCABE, W. R. & Jackson, G. G. Gram-negative bacteremia: I. Etiology and ecology. *Arch. Intern. Med.* **110**, 847–855 (1962).
121. Warren, M. A. *et al.* Severity scoring of lung oedema on the chest radiograph is associated with clinical outcomes in ARDS. *Thorax* **73**, 840–846 (2018).
122. Rouby, J.-J. *et al.* Regional distribution of gas and tissue in acute respiratory distress syndrome. II. Physiological correlations and definition of an ARDS Severity Score. *Intensive Care Med.* **26**, 1046–1056
123. Puybasset, L. *et al.* Regional distribution of gas and tissue in acute respiratory distress syndrome. I. Consequences for lung morphology. *Intensive Care Med.* **26**, 857–869 (2000).
124. Lichtenstein, D. *et al.* Comparative diagnostic performances of auscultation, chest radiography, and lung ultrasonography in acute respiratory distress syndrome. *Anesthesiology* **100**, 9–15 (2004).
125. Arbelot, C., Ferrari, F., Bouhemad, B. & Rouby, J.-J. Lung ultrasound in acute respiratory distress syndrome and acute lung injury. *Curr. Opin. Crit. Care* **14**, 70–74 (2008).
126. Avidan, M. S. *et al.* Prevention of intraoperative awareness in a high-risk surgical population. *N. Engl. J. Med.* **365**, 591–600 (2011).
127. Avidan, M. S. *et al.* Protocol for the BAG-RECALL clinical trial: a prospective, multi-center, randomized, controlled trial to determine whether a bispectral index-guided protocol is superior to an anesthesia gas-guided protocol in reducing intraoperative awareness with explicit recall in high risk surgical patients. *BMC Anesthesiol.* **9**, 8 (2009).
128. Hermans, G. *et al.* Interobserver agreement of Medical Research Council sum-score and handgrip strength in the intensive care unit. *Muscle Nerve* **45**, 18–25 (2012).
129. Laan, W. *et al.* Validity and reliability of the Katz-15 scale to measure unfavorable health outcomes in community-dwelling older people. *J. Nutr. Health Aging* **18**, 848–854 (2014).
130. Gerrard, P. The hierarchy of the activities of daily living in the Katz index in residents of skilled nursing facilities. *J. Geriatr. Phys. Ther.* **36**, 87–91 (2013).
131. Ahasic, A. M., Van Ness, P. H., Murphy, T. E., Araujo, K. L. B. & Pisani, M. A. Functional status after critical illness: agreement between patient and proxy assessments. *Age Ageing* **44**, 506–510 (2015).
132. Needham, D. M. *et al.* Study protocol: The Improving Care of Acute Lung Injury Patients

(ICAP) study. *Crit. Care* **10**, R9 (2006).
133. Needham, D. M. *et al.* Lung protective mechanical ventilation and two year survival in patients with acute lung injury: prospective cohort study. *BMJ* **344**, e2124 (2012).
134. Needham, D. M. *et al.* Physical and cognitive performance of patients with acute lung injury 1 year after initial trophic versus full enteral feeding. EDEN trial follow-up. *Am. J. Respir. Crit. Care Med.* **188**, 567–576 (2013).
135. Needham, D. M. *et al.* One year outcomes in patients with acute lung injury randomised to initial trophic or full enteral feeding: prospective follow-up of EDEN randomised trial. *BMJ* **346**, f1532 (2013).
136. ICH Harmonised Tripartite Guideline: Guideline for Good Clinical Practice. *J. Postgrad. Med.* **47**, 199–203 (2001).
137. ICH harmonised tripartite guideline: guideline for good clinical practice. 8. Essential documents for the conduct of a clinical trial. *J. Postgrad. Med.* **47**, 264–267 (2001).
138. Checkley, W., Brower, R. G., Muñoz, A. & NIH Acute Respiratory Distress Syndrome Network Investigators. Inference for mutually exclusive competing events through a mixture of generalized gamma distributions. *Epidemiology* **21**, 557–565 (2010).
139. Rothman, K. J. & Greenland, S. Modern epidemiology. 2nd. *Philadelphia, PA, Lippencott-Raven Publishers* (1998).
140. Feise, R. J. Do multiple outcome measures require p-value adjustment? *BMC Med. Res. Methodol.* **2**, 8 (2002).
141. Ellis, R. K. Determination of PO2 from saturation. *J. Appl. Physiol.* **67**, 902 (1989).
142. Severinghaus, J. W. Simple, accurate equations for human blood O2 dissociation computations. *J. Appl. Physiol.* **46**, 599–602 (1979).
143. Rice, T. W. *et al.* Comparison of the SpO2/FIO2 ratio and the PaO2/FIO2 ratio in patients with acute lung injury or ARDS. *Chest* **132**, 410–417 (2007).
144. Lanspa, M. J., Jones, B. E., Brown, S. M. & Dean, N. C. Mortality, morbidity, and disease severity of patients with aspiration pneumonia. *J. Hosp. Med.* **8**, 83–90 (2013).
145. Dean, N. C. *et al.* Hospital admission decision for patients with community-acquired pneumonia: variability among physicians in an emergency department. *Ann. Emerg. Med.* **59**, 35–41 (2012).
146. Brown, S. M., Jones, B. E., Jephson, A. R., Dean, N. C. & Infectious Disease Society of America/American Thoracic Society 2007. Validation of the Infectious Disease Society of America/American Thoracic Society 2007 guidelines for severe community-acquired pneumonia. *Crit. Care Med.* **37**, 3010–3016 (2009).
147. Van de Louw, A. *et al.* Accuracy of pulse oximetry in the intensive care unit. *Intensive Care Med.* **27**, 1606–1613 (2001).
148. Pugh, R. N., Murray-Lyon, I. M., Dawson, J. L., Pietroni, M. C. & Williams, R. Transection of the oesophagus for bleeding oesophageal varices. *Br. J. Surg.* **60**, 646–649 (1973).
149. Babor, T. F. *et al.* Types of alcoholics, I. Evidence for an empirically derived typology based on indicators of vulnerability and severity. *Arch. Gen. Psychiatry* **49**, 599–608 (1992).
150. Moss, M., Bucher, B., Moore, F. A., Moore, E. E. & Parsons, P. E. The role of chronic alcohol abuse in the development of acute respiratory distress syndrome in adults. *JAMA* **275**, 50–54 (1996).
151. Chanques, G. *et al.* The CAM-ICU has now a French 'official' version. The translation process of the 2014 updated Complete Training Manual of the Confusion Assessment Method for the Intensive Care Unit in French (CAM-ICU.fr). *Anaesth Crit Care Pain Med* **36**, 297–300 (2017).
152. Skrobik, Y. Le delirium aux soins intensifs. *Réanimation* **17**, 618–624 (2008).
153. Ware, J. E., Jr & Sherbourne, C. D. The MOS 36-item short-form health survey (SF-36). I. Conceptual framework and item selection. *Med. Care* **30**, 473–483 (1992).

19. Appendix list

Appendix A1: SpO_2/FiO_2 ratio inclusion criteria

Appendix A2: Exclusion definitions

Appendix B: Time-Events Schedule

Appendix C: Ventilator procedures
- **C1**: Ventilator management
- **C2**: Weaning

Appendix D: Conservative fluid management approach

Appendix G: SOFA scoring system

Appendix H: KDIGO criteria for acute kidney injury

Appendix I: Common risk factors for ARDS

Appendix K: Consort diagram of the SESAR trial

Appendix L: Curriculum vitae (principal coordinator)

Appendix M: Instruments and questionnaires (English and French versions)

Appendix N: Proposed sedation protocol for inhaled sedation with sevoflurane

Appendix A1: SpO_2/FiO_2 ratio inclusion criteria

The table below displays an equivalence table that determines the estimated PaO_2/FiO_2 ratio from the FiO_2 and SpO_2. This data was generated by investigators at the University of Utah, on a cohort of critically ill patients with pneumonia[136–142].

Imputed PaO_2/FiO_2 for combination of SpO_2 (rows) and FiO_2 (columns)

SPO2	FiO2														
	0.3	0.35	0.4	0.45	0.5	0.55	0.6	0.65	0.7	0.75	0.8	0.85	0.9	0.95	1
80%	148	127	111	98	89	81	74	68	63	59	55	52	49	47	44
81%	151	129	113	101	91	82	76	70	65	60	57	53	50	48	45
82%	155	132	116	103	93	84	77	71	66	62	58	55	52	49	46
83%	158	136	119	106	95	86	79	73	68	63	59	56	53	50	47
84%	162	139	122	108	97	89	81	75	70	65	61	57	54	51	49
85%	167	143	125	111	100	91	83	77	71	67	63	59	56	53	50
86%	171	147	129	114	103	94	86	79	73	69	64	61	57	54	51
87%	177	151	132	118	106	96	88	81	76	71	66	62	59	56	53
88%	182	156	137	121	109	99	91	84	78	73	68	64	61	58	55
89%	189	162	141	126	113	103	94	87	81	75	71	67	63	60	57
90%	196	168	147	130	117	107	98	90	84	78	73	69	65	62	59
91%	203	174	153	136	122	111	102	94	87	81	76	72	68	64	61
92%	213	182	159	142	128	116	106	98	91	85	80	75	71	67	64
93%	223	191	168	149	134	122	112	103	96	89	84	79	74	71	67
94%	236	202	177	157	142	129	118	109	101	94	89	83	79	75	71
95%	252	216	189	168	151	138	126	116	108	101	95	89	84	80	76
96%	273	234	205	182	164	149	136	126	117	109	102	96	91	86	82

For altitude adjustment, we would recommend the practice from ARDS Network studies of multiplying the qualification threshold PaO_2/FiO_2 by the ratio of average ambient to sea level barometric pressure (for Utah, it is 0.86*150 = 129; for Denver it is 0.84*150 = 126).

Additional requirements for the use of the SaO_2/FiO_2 ratio include:

1. SpO_2 between 80-96%
2. SpO_2 should be measured at least 10 minutes after any change in FiO_2.
3. PEEP ≥8 cmH_2O
4. An adequate pulse oximeter waveform tracing

Appendix A2: Exclusion definitions

Child-Pugh Score[143]

Premorbid values within 1 year of enrollment should be used.

Points	Class
5-6	A
7-9	B
≥ 10	C

	Numerical Score for Increasing Abnormality		
Measurement	1	2	3
Ascites	None	Present	Tense
Encephalopathy	None	Grade I or II	Grade III or IV
Bilirubin (mg/dl)	< 2	2-3	> 3
Albumin (g/L)	> 35	28-35	< 28
Prothrombin time (sec. prolonged)	1-4	4-10	> 10

NOTE: If using INR instead of Prothrombin time for Child-Pugh calculation, points for INR are as follows:

<1.7 = 1 point
1.7-2.3 = 2 points
>2.3 = 3 points

Appendix B: Time-Events Schedule

Measurement/Event	Day 0	1	2	3	4	5	6	7	14	28	90	365
Demographics, History and Physical	X											
Etiology of ARDS	X											
APACHE II, SAPS II	X											
HCG (females)	X											
Alcohol Use/Smoking History	X											
Study sevoflurane administration (maximum)	X	(X)	(X)	(X)	(X)	(X)	(X)	(X)				
Level of sedation	X	X	X	X	X	X	X	X				
Bispectral index value	A	A	A	A	A	A	A	A				
Sedation interruption (Y/N)	N	Y	Y	Y	Y	Y	Y	Y				
Ventilator parameters	X	X	X	X	X	X	X	X				
Hemodynamic parameters	X	X	X	X	X	X	X	X				
Arterial Blood Gases	X	X	X	A	A	A	A	A				
K^+, HCO_3^-, glucose	A	A	A	A	A	A	A	A				
Serum liver function tests (AST, ALT, bilirubin, INR)	X	X	X	X	X	X	X	X				
KDIGO criteria for acute kidney injury	X	X	X	X	X	X	X	X	X			
Pneumothorax (Y/N)	X	X	X	X	X	X	X	X				
Use of rescue procedures (Y/N)	X	X	X	X	X	X	X	X				
Central Venous Pressure	A	A	A	A	A	A	A	A				
On-study meds: sedative, opioid, corticosteroid	X	X	X	X	X	X	X	X				

administration (Y/N)											
SOFA Score (modified)[B]	X	X	X	X	X	X	X	X	X		
CAM-ICU	X	X	X	X	X	X	X				
Plasma Collection	X	X	X		X		X		X **#		
Urine Collection	X	X	X		X		X		X **#		
Whole Blood Collection for DNA/RNA studies	X		X								
Bronchoalveolar lavage	X			X							
Undiluted pulmonary edema fluid[£]	X	X									
Collection of heat moisture exchanger filters[£]		X									
Collection of AnaConDa-S filters[*,£]		X									
Number of ventilator-free days								X	X	X		
Disability (KATZ ADL)											X	X
Health-Related Quality of Life (SF-36)											X	X
Self-rated health											X	X
Pain-interference											X	X
Post-Traumatic Stress-like Symptoms (PTSS-14, HADS)											X	X
Cognitive Function (AD-8)											X	X
Subsequent return to work, hospital and ED use, and location of residence											X	X
Vital Status								X	X	X	X	X

X = Required
A = When available
* = in patients enrolled in the intervention arm
**# = or at ICU discharge, whichever occurs first
B = Records clinically available creatinine, platelets, bilirubin, SBP and vasopressor use
# = Measure during reference period (06:00-10:00); other values may be obtained closest to 08:00 on the specified calendar date
£ = in selected centers only, due to logistical considerations (n=30 patients from each group)
$ = in 5 selected centers only, due to logistical considerations (total n=25 patients)

Appendix C: Ventilator procedures

C1: Ventilator management

We will use a simplified version of the ARDS network 6 mL/kg PBW lung-protective ventilation protocol[5] except that controlled modes of ventilation will be required during the period of neuromuscular blockade. If not already being used, a low tidal volume protocol for mechanical ventilation will be initiated within two hours of randomization in all patients. Using volume-controlled ventilation, the tidal volume (Vt) will be set at 6 mL/kg (+/- 2 mL/kg) of PBW[32] and PEEP will be adjusted based on airway pressure and kept as high as possible without increasing the maximal inspiratory plateau pressure above 28 to 30 cmH_2O, such as in the Expiratory Pressure (Express) Study[31]; therefore, PEEP will be individually titrated based on plateau pressure, regardless of its effect on oxygenation in contrast to the PEEP/FiO_2 scales used in some studies[49,117].

We will recommend sites to wait at least 12 hours (as per PROSEVA[29]) before proning. As recommended by recent international guidelines[32], proning will be applied in patients with severe ARDS for more than 12 hours/day; proning will eventually be applied more than once, as per the treating clinicians.

We will allow deviation from the high PEEP strategy, for limited situations:

- If there is clinical concern that the use of high PEEP may be worsening oxygenation (e.g., oxygenation worsens with PEEP increases) at a FiO_2 ≥0.5 for more than 2 hours, clinicians may trial lower PEEP.
- If oxygenation worsens or is unchanged at the lower level of PEEP, the PEEP should be raised back to the previous level.
- If hypotension and/or high Pplat (>30 cmH_2O) are present despite further tidal volume reduction, fluid boluses, and/or respiratory rate increase, lower PEEP may be used. It will then be allowed to reduce PEEP 2 cmH_2O every 5-15 minutes, until the physiologic parameters of concern have improved, as per the treating clinician and/or responsible investigator (e.g., reduce PEEP to the level that lowers plateau pressure to 30 cmH_2O). Later, the clinician tries to return PEEP to a level consistent with the mechanical strategy described above.

Lower PEEP may also be used if a study participant develops a pneumothorax, is deemed at high risk for barotrauma (e.g., known multiple pulmonary cysts or bullae) or as per the treating clinicians.

Predicted body weight (PBW) is calculated from gender and height (heel to crown) according to the following equations:

Males:

PBW (kg) = 50 + 2.3 [height (inches) – 60]
PBW (kg) = 50 + 0.91 [height (centimeters) – 152.4]

Females:

PBW (kg) = 45.5 + 2.3 [eight (inches) – 60]
PBW (kg) = 45.5 + 0.91 [height (centimeters) – 152.4]

Oxygenation target: 55 mmHg $<PaO_2<$80 mmHg or 88% $<SpO_2<$95%. When both PaO_2 and SpO_2 are available simultaneously, the PaO_2 criterion will take precedence.

No specific rules for respiratory rate, but incremental increase in the RR to maximum set rate of 35 if pH <7.30.

I: E ratio of at least 1/2.

Bicarbonate infusion is allowed (neither encouraged nor discouraged) if pH <7.30.

Changes in more than one ventilator setting driven by measurements of PaO_2, pH, and Pplat may be performed simultaneously, if necessary.

In the intervention arm, we will only allow deviation from the inhaled sedation strategy (interruption of sevoflurane administration and removal of the AnaConDa-S from the breathing circuit) if severe acidemia (pH <7.15) is present, in the absence of metabolic acidosis, and despite further tidal volume and/or respiratory rate increase, or if malignant hyperthermia or a bronchopleural fistula that is persistent despite drainage (to limit room exposure) develops under inhaled sedation. In this situation, patients from the intervention arm will be switched to an intravenous sedation strategy using propofol.

C2: Weaning from mechanical ventilation

Commencement of weaning

Patients will be assessed for the following weaning readiness criteria each day between 06:00 and 10:00. If a patient procedure, test, or other extenuating circumstance prevents assessment for these criteria between 06:00 and 10:00, then the assessment and initiation of subsequent weaning procedures may be delayed for up to six hours.

Patients can be assessed for weaning readiness criteria twice a day:

1. At least 12 hours since enrollment in the trial
2. FiO_2 ≤0.50 and PEEP ≤8 cmH_2O
3. Values of both PEEP and FiO_2 ≤values from previous day
4. Systolic arterial pressure ≥90 mmHg without vasopressor support (≤0.5 µg/kg/min; dopamine will not be considered a vasopressor)

Spontaneous breathing trial (SBT) procedure and assessment for unassisted breathing

If criteria 1-4 above are met, first the neuromuscular blocking agent will need to be discontinued if the medication is still being infused. When the neuromuscular blocking agent has worn off and the patient is having spontaneous respirations, then initiate a trial of 60 minutes of spontaneous breathing with FiO_2 ≥0.5 using any of the following approaches:

1. Pressure support of 8-10 cmH_2O with PEEP = 0 cmH_2O
2. CPAP ≤5 cmH_2O
3. T-piece
4. Tracheostomy mask

Monitor for tolerance using the following:

1. SpO2 ≥90% and / or PaO2 ≥60 mmHg
2. Mean spontaneous tidal volume ≥4 mL/kg PBW (if measured)
3. Respiratory Rate ≤35 /min

4. pH ≥7.30 (if measured)
5. No respiratory distress (defined as 2 or more of the following):
    a. Heart rate ≥120% of the 06:00 rate (≤5 min at >120% may be tolerated)
    b. Marked use of accessory muscles
    c. Abdominal paradox
    d. Diaphoresis
    e. Marked subjective dyspnea.

If any of the goals 1-5 are not met, revert to previous ventilator settings or to the pressure support level needed to reach a Vt of 6 mL/kg of PBW, with PEEP and FiO_2 = previous settings and reassess for weaning the next morning.

The clinical team may decide to change the mode of support during spontaneous breathing (PS = 5, CPAP, tracheostomy mask, or T-piece) at any time.

The AnaConDa-S will be removed from the breathing circuit as soon as inhaled sedation is interrupted. The AnaConDa-S should be removed from the breathing circuit for spontaneous breathing trial.

Decision to remove ventilatory support

For intubated patients, if tolerance criteria for spontaneous breathing trial (1-5 above) are met for 60 minutes, the clinical team will decide to extubate.

If any of criteria 1-5 are not met during unassisted breathing, then the ventilator settings that were in use before the attempt to wean will be restored and the patient will be reassessed for weaning the following day.

Definition of unassisted breathing

a) Extubated with face mask, nasal prong oxygen, or room air, OR
b) T-tube breathing, OR
c) Tracheostomy mask breathing, OR
d) CPAP ≤5 without PS or IMV assistance
e) Use of CPAP or BIPAP solely for sleep apnea management
f) Use of a high flow oxygen system

For an uninterrupted period of at least 24 hours or more.

Completion of ventilator procedures

Patients will be considered to have completed the study ventilator procedures if any of the following conditions occur:

a. Death
b. Hospital discharge
c. Alive 28 days after enrollment

If a patient requires positive pressure ventilation after a period of unassisted breathing, the study ventilator procedures will resume unless the patient was discharged from the hospital or >28 days elapsed since enrollment.

Appendix D: Conservative fluid management approach

A modified conservative fluid protocol will be used based on the findings from FACTT that conservative fluid management increased ventilator-free days. This protocol is recommended for all enrolled patients, to be used until study day 7, whichever occurs first.

1. Discontinue maintenance fluids.
2. Continue medications and nutrition.
3. Manage electrolytes and blood products per usual practice.
4. For shock, use any combination of fluid boluses[#] and vasopressor(s) to achieve MAP ≥60 mmHg as fast as possible. Wean vasopressors as quickly as tolerated beginning four hours after blood pressure has stabilized.
5. Withhold diuretic therapy in renal failure[§] and until 12 hours after last fluid bolus or vasopressor given.

This protocol is a simplified modification of the conservative protocol used in FACTT. For patients without a CVC, no fluid gain over the first 7 study days is recommended once patients' blood pressure has stabilized. Stable blood pressure is defined as no requirement for either vasopressors or a fluid bolus to support blood pressure for 12 or more hours.

<table>
<tr><th rowspan="2">CVP
(recommended)</th><th rowspan="2">PAOP
(optional)</th><th colspan="2">MAP ≥ 60 mm Hg AND off vasopressors for ≥ 12 hours</th></tr>
<tr><th>Average urine output < 0.5 ml/kg/hr</th><th>Average urine output ≥ 0.5 ml/kg/hr</th></tr>
<tr><td>>8</td><td>> 12</td><td>Furosemide*
Reassess in 1 hour</td><td rowspan="2">Furosemide*
Reassess in 4 hours</td></tr>
<tr><td>4-8</td><td>8-12</td><td rowspan="2">Give fluid bolus as fast as possible[#]
Reassess in 1 hour</td></tr>
<tr><td>< 4</td><td>< 8</td><td>No intervention
Reassess in 4 hours</td></tr>
</table>

[§] Renal failure is defined as dialysis dependence, oliguria with serum creatinine >3 mg/dL, or oliguria with serum creatinine 0-3 with urinary indices indicative of acute renal failure.
[#] Recommended fluid bolus = 15 mL/kg crystalloid (round to nearest 250 mL) or 1 Unit packed red cells or 25 grams albumin
* Recommended Furosemide dosing = begin with 20 mg bolus or 3 mg/hr infusion or last known effective dose. Double each subsequent dose until goal achieved (oliguria reversal or intravascular pressure target) or maximum infusion rate of 24 mg/hr or 160 mg bolus reached. Do not exceed 620 mg/day. Also, if the patient has heart failure, consider treatment with dobutamine.

Appendix G: SOFA scoring system

Sepsis-related organ failure assessment (SOFA) scoring (excluding Glasgow Coma Score)

Organ system	0	1	2	3	4
**Respiration** PaO_2/FiO_2 (mmHg)	>400	301 – 400	<301 (without respiratory support*)	101 – 200 (without respiratory support*)	≤100 (without respiratory support*)
**Coagulation** Platelets ($x10^3/mm^3$)	>150	101 – 150	51 – 100	21 – 50	≤20
**Liver** Bilirubin (µmol/L)	<20	20 – 32	33 – 101	102 – 204	>204
**Cardiovascular** Hypotension	MAP >70 mmHg	MAP <70 mmHg	dopamine ≤5.0 (µg/kg/min)	dopamine >5.0 (µg/kg/min)	dopamine >15.0 (µg/kg/min)
			or any dose dobutamine	or norepinephrine ≤0.1 or epinephrine ≤0.1	or norepinephrine >0.1 or epinephrine >0.1
**Renal** Creatinine (µmol/L)	<110	110 – 170	171 – 299	300 – 440	>440
OR urine output				or <500 mL/day	or <200 mL/day

The most deranged value recorded in the previous 24 h is to be used. If a value has not been measured, the score 0 should be given.

* Respiratory support is defined as any form of invasive or non-invasive ventilation including continuous positive airway pressure delivered through mask or tracheotomy

Appendix H: KDIGO criteria for acute kidney injury

Stage	Serum creatinine	Urine output
**1**	1.5-1.9 times baseline<br>or ≥0.3 mg/dL (≥26.5 µmol/L) increase	<0.5 ml/kg/h for 6-12 h
**2**	2.0-2.9 times baseline	<0.5 ml/kg/h for ≥12 h
**3**	3 times baseline<br>or ≥4.0 mg/dL (≥353.6 µmol/L) increase<br>or initiation of RRT	<0.3 ml/kg/h ≥24 h<br>or anuria ≥12 h

Appendix I: Common risk factors for ARDS

Direct

Pneumonia
Aspiration of gastric contents
Inhalational injury
Pulmonary contusion
Drowning

Indirect

Non-pulmonary sepsis
Major trauma
Pancreatitis
Severe burns
Non-cardiogenic shock
Drug overdose
Multiple transfusions or transfusion associated acute lung injury (TRALI)

Source: Supplementary Online Content, eTable 1. The ARDS Definition Task Force. Acute respiratory distress syndrome: the Berlin Definition. JAMA. doi:10.1001/jama.2012.5669.[1]

Appendix K: CONSORT diagram of the SESAR trial

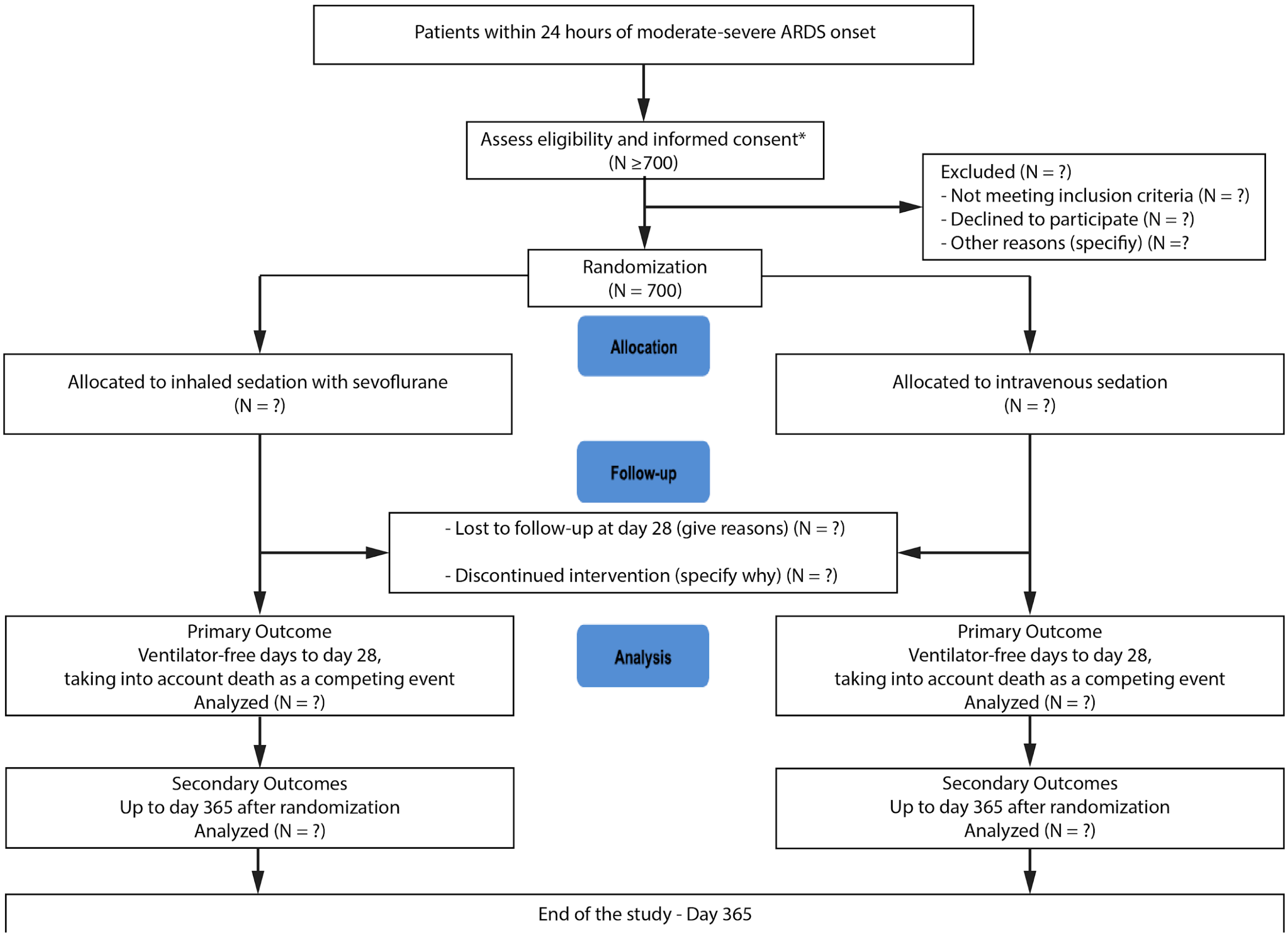

* Because, in emergency situations, sedation and ventilation must be initiated as early as possible, the study protocol provides for a waiver of informed consent from the patient. The consent from the patient's next of kin will therefore be sought actively during the 24-hour enrollment time window. In case the patient's next of kin cannot be reached in a timely manner, the investigator will decide to include the patient in the study using an emergent consent procedure. Deferred informed consent will be obtained from participants for potential continuation of the research.

Appendix L: Curriculum vitae (principal coordinator)

Name (First, last): JABAUDON Matthieu

Function: Associate Professor of Anesthesiology and Critical Care Medicine

Medical order registration (CNOM): **63/5679**
ADELI number: **63 10 5679 3**
RPPS number: **10005175210**

Affiliations:
Department of Perioperative Medicine (Pr Bazin), CHU Clermont-Ferrand
Université Clermont Auvergne, CNRS UMR 6293, INSERM U1103, GReD

Professional address:
Department of Perioperative Medicine, CHU Clermont-Ferrand
1, Place Lucie et Raymond Aubrac, 63003 Clermont-Ferrand Cedex 1
Email: mjabaudon@chu-clermontferrand.fr
Tel: +33 (0)4 73 750 501 / Fax: +33 (0)4 73 750 500

Recent publication (5):

1. Jabaudon M, Godet T, Futier E, et al. Rationale, study design, and analysis plan of the Lung Imaging morphology for Ventilator settings in acute respiratory distress syndrome study (LIVE study): study protocol for a randomised controlled trial. Anaesth Crit Care Pain Med. 2017 Oct; 36(5):301-306.
2. Jabaudon M, Boucher P, Imhoff E, et al. Sevoflurane for Sedation in ARDS: A Randomized Controlled Pilot Study. Am J Respir Crit Care Med. 2017 Mar 15; 195(6):792-800.
3. Mrozek S, Jabaudon M, Jaber S, et al. Elevated Plasma Levels of sRAGE Are Associated With Nonfocal CT-Based Lung Imaging in Patients With ARDS: A Prospective Multicenter Study. Chest. 2016 Nov;150(5):998-1007.
4. Jabaudon M, Blondonnet R, Roszyk L, et al. Soluble Receptor for Advanced Glycation End-Products Predicts Impaired Alveolar Fluid Clearance in Acute Respiratory Distress Syndrome. Am J Respir Crit Care Med. 2015 Jul 15; 192(2):191-9.
5. Jabaudon M, Hamroun N, Roszyk L, et al. Effects of a recruitment maneuver on plasma levels of soluble RAGE in patients with diffuse acute respiratory distress syndrome: a prospective randomized crossover study. Intensive Care Med. 2015 May; 41(5):846-55.

Date: 27/08/2018 **Signature:**

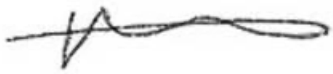

Appendix M: Instruments and questionnaires (English + French versions)

Confusion Assessment Method for the ICU (CAM-ICU)[4,5]

Instructions: To evaluate for the presence of delirium in your patient, complete this clinical assessment every shift (8-12 hours).

CAM-ICU is a valid and reliable delirium assessment tool recommended by the Society of Critical Care Medicine (SCCM) in its 2013 Pain, Agitation, and Delirium (PAD) guidelines.

CAM-ICU	Criteria	✓ Present
**FEATURE 1: Alteration/Fluctuation in Mental Status**		
▪ Is the patient's mental status different than his/her baseline? **OR**<br>▪ Has the patient had any fluctuation in mental status in the past 24 hours as evidenced by fluctuation on a sedation scale (eg, RASS, Glasgow Coma Scale [GCS]), or previous delirium assessment?	If Yes for either question ▶	☐
**FEATURE 2: Inattention 1: Alteration/Fluctuation in Mental Status**		
**Letters Attention Test:**<br>Tell the patient *"I am going to read to you a series of 10 letters. Whenever you hear the letter 'A,' squeeze my hand."*<br>S A V E A H A A R T<br>Count errors (each time patient fails to squeeze on the letter "A" and squeezes on a letter other than "A").	If number of errors >2 ▶	☐
**FEATURE 3: Altered Level of Consciousness (LOC)**		
▪ Present if the RASS score is anything other than Alert and Calm (zero) **OR**<br>▪ If SAS is anything other than Calm (4)	If RASS ≠0 **OR** SAS ≠4 ▶	☐
**FEATURE 4: Disorganized Thinking**		
**Yes/No Questions:** Ask the patient to respond:<br>1. Will a stone float on water?<br>2. Are there fish in the sea?<br>3. Does 1 pound weigh more than 2 pounds?<br>4. Can you use a hammer to pound a nail?<br>Count errors (each time patient answers incorrectly).<br>**Commands:** Ask the patient to follow your instructions:<br>a) *"Hold up this many fingers."* (Hold 2 fingers in front of the patient.)<br>b) *"Now do the same thing with the other hand."* (Do **not** demonstrate the number of fingers this time.)<br>◻ If unable to move both arms, for part "b" of command ask patient to "Hold up one more finger."<br>Count errors if patient is unable to complete the entire command.	If combined number of errors >1 ▶	
If Features 1 and 2 are both present and either Features 3 or 4 are present: *CAM-ICU is positive, delirium is present*	**Delirium present** ☐<br>**Delirium absent** ☐	

Le score peut être utilisé uniquement chez les patients capables d'ouvrir les yeux et de serrer la main à la demande

Critère 1 : Début soudain et évolution fluctuante

- La modification de l'état mental du patient est-elle soudaine ? (oui = positif/non = négatif)
- Y-a-t-il des fluctuations sur les dernières 24 heures ? (oui = positif/non = négatif)

Critère 2 : Inattention

- Le patient présente-t-il des difficultés à focaliser son attention ?
  - Test «ABRACADABRA» : Epelez chaque lettre (1-2 secondes entre chaque lettre) et demandez au patient de serrer la main sur la lettre «A» :

Le test est positif s'il y a plus de deux erreurs (soit le patient ne sert pas la main sur la lettre A, soit il la sert sur une autre lettre)

Critère 3 : Désorganisation de la pensée

- Question oui/non :
  - Est-ce qu'une pierre flotte sur l'eau ?
  - Y-a-t-il des poissons dans la mer ?
  - Est-ce qu'un kilogramme pèse plus que deux kilogrammes ?
  - Peut-on utiliser un marteau pour enfoncer un clou ?

Le test est positif s'il y a plus d'une réponse fausse

- Commandes verbales :
  - Dites au patient : «Montrez-moi le même nombre de doigts» (Montrez-lui deux doigts)
  - Puis «Faites la même chose avec l'autre main»

Le test est positif si le patient se trompe dans ces deux commandes

Etape 4 : Evaluation de la vigilance

- Globalement, comment évalueriez-vous l'état général de votre patient ? Alerte, vigile, léthargique, stuporeux, comateux

Le test est positif si le niveau de conscience est autre qu'alerte

Le diagnostic de delirium requiert la présence de 3 des 4 critères. Les critères 1 et 2 sont toujours requis, accompagnés du critère 3 ou 4

Katz Activities of Daily Living (ADL)[9]

Katz Index of Independence in Activities of Daily Living		
**Activities**<br>Points (1 or 0)	**Independence**<br>(1 Point)<br><br>**NO** supervision, direction or personal assistance.	**Dependence**<br>(0 Points)<br><br>**WITH** supervision, direction, personal assistance or total care.
**BATHING**<br><br>Points: __________	**(1 POINT)** Bathes self completely or needs help in bathing only a single part of the body such as the back, genital area or disabled extremity.	**(0 POINTS)** Need help with bathing more than one part of the body, getting in or out of the tub or shower. Requires total bathing
**DRESSING**<br><br>Points: __________	**(1 POINT)** Get clothes from closets and drawers and puts on clothes and outer garments complete with fasteners. May have help tying shoes.	**(0 POINTS)** Needs help with dressing self or needs to be completely dressed.
**TOILETING**<br><br>Points: __________	**(1 POINT)** Goes to toilet, gets on and off, arranges clothes, cleans genital area without help.	**(0 POINTS)** Needs help transferring to the toilet, cleaning self or uses bedpan or commode.
**TRANSFERRING**<br><br>Points: __________	**(1 POINT)** Moves in and out of bed or chair unassisted. Mechanical transfer aids are acceptable	**(0 POINTS)** Needs help in moving from bed to chair or requires a complete transfer.
**CONTINENCE**<br><br>Points: __________	**(1 POINT)** Exercises complete self control over urination and defecation.	**(0 POINTS)** Is partially or totally incontinent of bowel or bladder
**FEEDING**<br><br>Points: __________	**(1 POINT)** Gets food from plate into mouth without help. Preparation of food may be done by another person.	**(0 POINTS)** Needs partial or total help with feeding or requires parenteral feeding.
**TOTAL POINTS:** ________	**SCORING:** 6 = High (*patient independent*) 0 = Low (*patient very dependent*	

**ECHELLE A.D.L** (Aide-soignante Infirmière)	1ère évaluation<br>Date :<br>Score:	2ème évaluation<br>Date :<br>Score:	3ème évaluation<br>Date :<br>Score:
**HYGIENE CORPORELLE**			
. autonomie	1	1	1
. aide	½	½	½
. dépendant(e)	0	0	0
**HABILLAGE**			
. autonomie pour le choix des vêtements et l'habillage	1	1	1
. autonomie pour le choix des vêtements, l'habillage mais a besoin d'aide pour se chausser	½	½	½
. dépendant(e)	0	0	0
**ALLER AUX TOILETTES**			
. autonomie pour aller aux toilettes, se déshabiller et se rhabiller ensuite	1	1	1
. doit être accompagné(e) ou a besoin d'aide pour se déshabil-ler ou se rhabiller	½	½	½
. ne peut aller aux toilettes seul(e)	0	0	0
**LOCOMOTION**			
. autonomie	1	1	1
. a besoin d'aide	½	½	½
. grabataire	0	0	0
**CONTINENCE**			
. continent(e)	1	1	1
. incontinence occasionnelle	½	½	½
. incontinent(e)	0	0	0
**REPAS**			
. mange seul(e)	1	1	1
. aide pour couper la viande ou peler les fruits	½	½	½
. dépendant(e)	0	0	0
**TOTAL**			

Short Form-36 (SF-36)[12]

SF-36 QUESTIONNAIRE

Name:___________________ **Ref. Dr:**__________________ **Date:** _______

ID#: ______________ **Age:** _______ **Gender: M / F**

Please answer the 36 questions of the **Health Survey** completely, honestly, and without interruptions.

GENERAL HEALTH:
In general, would you say your health is:
◯ Excellent ◯ Very Good ◯ Good ◯ Fair ◯ Poor

Compared to one year ago, how would you rate your health in general now?
◯ Much better now than one year ago
◯ Somewhat better now than one year ago
◯ About the same
◯ Somewhat worse now than one year ago
◯ Much worse than one year ago

LIMITATIONS OF ACTIVITIES:
The following items are about activities you might do during a typical day. Does your health now limit you in these activities? If so, how much?

Vigorous activities, such as running, lifting heavy objects, participating in strenuous sports.
◯ Yes, Limited a lot ◯ Yes, Limited a Little ◯ No, Not Limited at all

Moderate activities, such as moving a table, pushing a vacuum cleaner, bowling, or playing golf
◯ Yes, Limited a Lot ◯ Yes, Limited a Little ◯ No, Not Limited at all

Lifting or carrying groceries
◯ Yes, Limited a Lot ◯ Yes, Limited a Little ◯ No, Not Limited at all

Climbing several flights of stairs
◯ Yes, Limited a Lot ◯ Yes, Limited a Little ◯ No, Not Limited at all

Climbing one flight of stairs
◯ Yes, Limited a Lot ◯ Yes, Limited a Little ◯ No, Not Limited at all

Bending, kneeling, or stooping
◯ Yes, Limited a Lot ◯ Yes, Limited a Little ◯ No, Not Limited at all

Walking more than a mile
◯ Yes, Limited a Lot ◯ Yes, Limited a Little ◯ No, Not Limited at all

Walking several blocks
◯ Yes, Limited a Lot ◯ Yes, Limited a Little ◯ No, Not Limited at all

Walking one block
◯ Yes, Limited a Lot ◯ Yes, Limited a Little ◯ No, Not Limited at all

Bathing or dressing yourself
○Yes, Limited a Lot ○Yes, Limited a Little ○No, Not Limited at all

PHYSICAL HEALTH PROBLEMS:
During the past 4 weeks, have you had any of the following problems with your work or other regular daily activities as a result of your physical health?

Cut down the amount of time you spent on work or other activities
○Yes ○No

Accomplished less than you would like
○Yes ○No

Were limited in the kind of work or other activities
○Yes ○No

Had difficulty performing the work or other activities (for example, it took extra effort)
○Yes ○No

EMOTIONAL HEALTH PROBLEMS:
During the past 4 weeks, have you had any of the following problems with your work or other regular daily activities as a result of any emotional problems (such as feeling depressed or anxious)?

Cut down the amount of time you spent on work or other activities
○Yes ○No

Accomplished less than you would like
○Yes ○No

Didn't do work or other activities as carefully as usual
○Yes ○No

SOCIAL ACTIVITIES:
Emotional problems interfered with your normal social activities with family, friends, neighbors, or groups?

○Not at all ○Slightly ○Moderately ○Severe ○Very Severe

PAIN:
How much bodily pain have you had during the past 4 weeks?

○None ○Very Mild ○Mild ○Moderate ○Severe ○Very Severe

During the past 4 weeks, how much did pain interfere with your normal work (including both work outside the home and housework)?

○Not at all ○A little bit ○Moderately ○Quite a bit ○Extremely

ENERGY AND EMOTIONS:
These questions are about how you feel and how things have been with you during the last 4 weeks. For each question, please give the answer that comes closest to the way you have been feeling.

Did you feel full of pep?
- All of the time
- Most of the time
- A good Bit of the Time
- Some of the time
- A little bit of the time
- None of the Time

Have you been a very nervous person?
- All of the time
- Most of the time
- A good Bit of the Time
- Some of the time
- A little bit of the time
- None of the Time

Have you felt so down in the dumps that nothing could cheer you up?
- All of the time
- Most of the time
- A good Bit of the Time
- Some of the time
- A little bit of the time
- None of the Time

Have you felt calm and peaceful?
- All of the time
- Most of the time
- A good Bit of the Time
- Some of the time
- A little bit of the time
- None of the Time

Did you have a lot of energy?
- All of the time
- Most of the time
- A good Bit of the Time
- Some of the time
- A little bit of the time
- None of the Time

Have you felt downhearted and blue?

- All of the time
- Most of the time
- A good Bit of the Time
- Some of the time
- A little bit of the time
- None of the Time

Did you feel worn out?

- All of the time
- Most of the time
- A good Bit of the Time
- Some of the time
- A little bit of the time
- None of the Time

Have you been a happy person?

- All of the time
- Most of the time
- A good Bit of the Time
- Some of the time
- A little bit of the time
- None of the Time

Did you feel tired?

- All of the time
- Most of the time
- A good Bit of the Time
- Some of the time
- A little bit of the time
- None of the Time

SOCIAL ACTIVITIES:
During the past 4 weeks, how much of the time has your physical health or emotional problems interfered with your social activities (like visiting with friends, relatives, etc.)?

- All of the time
- Most of the time
- Some of the time
- A little bit of the time
- None of the Time

QUESTIONNAIRE SF-36

1. Dans l'ensemble, pensez-vous que votre santé est :

Excellente	Très bonne	Bonne	Médiocre	Mauvaise
❑	❑	❑	❑	❑

2. Par rapport à l'année dernière à la même époque, comment trouvez-vous votre état de santé en ce moment ?

Bien meilleur que l'an dernier	Plutôt meilleur	A peu près pareil	Plutôt moins bon	Beaucoup moins bon
❑	❑	❑	❑	❑

3. Voici une liste d'activités que vous pouvez avoir à faire dans votre vie de tous les jours. Pour chacune d'entre elles indiquez si vous êtes limité(e) en raison de votre état de santé actuel.

Cochez la réponse de votre choix, une par ligne

	**Oui, très limité**	**Oui, plutôt limité**	**Non, pas du tout limité**
**Efforts physiques importants** (courir, soulever un objet lourd, faire du sport)	❑	❑	❑
**Efforts physiques modérés** (déplacer une table, passer l'aspirateur, jouer aux boules)	❑	❑	❑
Soulever et porter les courses	❑	❑	❑
Monter **plusieurs étages** par l'escalier	❑	❑	❑
Monter **un étage** par l'escalier	❑	❑	❑
Se pencher en avant, se mettre à genoux, s'accroupir	❑	❑	❑
Marcher **plus d'un km** à pied	❑	❑	❑
Marcher **plusieurs centaines de mètres**	❑	❑	❑
Marcher **une centaine de mètres**	❑	❑	❑
Prendre un bain, une douche ou s'habiller	❑	❑	❑

4. Au cours de ces 4 dernières semaines, et en raison de votre état physique.

Cochez la réponse de votre choix, une par ligne

	OUI	NON
Avez-vous réduit **le temps passé** à votre travail ou à vos activités habituelles ?	❑	❑
Avez-vous **accompli moins** de choses que ce que vous auriez souhaité ?	❑	❑
Avez-vous dû arrêter de faire **certaines** choses ?	❑	❑
Avez-vous eu des **difficultés** à faire votre travail ou toute autre activité ? (par ex : cela vous a demandé un effort supplémentaire)	❑	❑

5. Au cours de ces 4 dernières semaines, et en raison de votre état émotionnel (comme vous sentir triste, nerveux(se), ou déprimé(e).

Cochez la réponse de votre choix, une par ligne

	OUI	NON
Avez-vous réduit **le temps passé** à votre travail ou à vos activités habituelles ?	❑	❑
Avez-vous **accompli moins** de choses que ce que vous auriez souhaité ?	❑	❑
Avez-vous eu des **difficultés** à faire ce que vous aviez à faire **avec autant de soin et d'attention que d'habitude** ?	❑	❑

6. Au cours de ces 4 dernières semaines dans quelle mesure votre état de santé, physique ou émotionnel, vous a-t-il gêné(e) dans votre vie sociale et vos relations avec les autres, votre famille, vos amis, vos connaissances ?

Pas du tout	Un petit peu	Moyennement	Beaucoup	Enormément
❑	❑	❑	❑	❑

7. Au cours de ces 4 dernières semaines, quelle a été l'intensité de vos douleurs physiques ?

Nulle	Très faible	Faible	Moyenne	Grande	Très grande
❑	❑	❑	❑	❑	❑

8. Au cours de ces 4 dernières semaines, dans quelle mesure vos douleurs physiques vous ont-elles limité(e) dans votre travail ou vos activités domestiques ?

Pas du tout	Un petit peu	Moyennement	Beaucoup	Enormément
❑	❑	❑	❑	❑

9. Les questions qui suivent portent sur comment vous vous êtes senti(e) au cours de ces 4 dernières semaines. Pour chaque question, veuillez indiquer la réponse qui vous semble la plus appropriée. Au cours de ces 4 dernières semaines, y a-t-il eu des moments où :

Cochez la réponse de votre choix, une par ligne

	en permanence	très souvent	souvent	quelque fois	rarement	jamais
Vous vous êtes senti(e) dynamique ?	❑	❑	❑	❑	❑	❑
Vous vous êtes senti(e) nerveux ?	❑	❑	❑	❑	❑	❑
Vous vous êtes senti(e) si découragé(e) que rien ne pouvait vous remonter le moral ?	❑	❑	❑	❑	❑	❑
Vous vous êtes senti(e) calme et détendu(e) ?	❑	❑	❑	❑	❑	❑
Vous vous êtes senti(e) débordant(e) d'énergie ?	❑	❑	❑	❑	❑	❑
Vous vous êtes senti(e) triste et abattu(e) ?	❑	❑	❑	❑	❑	❑
Vous vous êtes senti(e) épuisé(e) ?	❑	❑	❑	❑	❑	❑
Vous vous êtes senti(e) heureux ?	❑	❑	❑	❑	❑	❑
Vous vous êtes senti(e) fatigué(e) ?	❑	❑	❑	❑	❑	❑

10. Au cours de ces 4 dernières semaines y a-t-il eu des moments où votre état de santé, physique ou émotionnel, vous a gêné(e) dans votre vie sociale et vos relations avec les autres, votre famille, vos amis, vos connaissances ?

En permanence	Une bonne partie du temps	De temps en temps	Rarement	Jamais
❑	❑	❑	❑	❑

11. Indiquez, pour chacune des phrases suivantes, dans quelle mesure elles sont vraies ou fausses dans votre cas :

Cochez la réponse de votre choix, une par ligne

	totalement vraie	plutôt vraie	je ne sais pas	plutôt fausse	totalement fausse
Je tombe malade plus facilement que les autres	❑	❑	❑	❑	❑
Je me porte aussi bien que n'importe qui	❑	❑	❑	❑	❑
Je m'attends à ce que ma santé se dégrade	❑	❑	❑	❑	❑
Je suis en excellente santé	❑	❑	❑	❑	❑

Self-rated health (1 item)

In general, would you say that your **health** is

- Excellent?
- Very good?
- Good?
- Fair?
- Poor?

D'une manière générale, diriez-vous que votre état de santé est:

- Excellent ?
- Très bon ?
- Bon ?
- Correct ?
- Mauvais

Pain Interference (1 item)

In the past 7 days, how much did pain interfere with your day to day activities ?

- Not at all
- A little bit
- Somewhat
- Quite a bit
- Very much

Au cours des 7 derniers jours, à quel point la douleur a-t-elle interféré avec vos activités quotidiennes ?

- Pas du tout
- Très peu
- Quelque peu
- Modérément
- Beaucoup

Post-Traumatic Stress Symptoms (PTSS-14)[14,15]

The UK-PTSS-14.

Presently (this means in the past few days) I suffer from:

1. Sleep problems
2. Nightmares
3. Depression, I feel dejected/downtrodden
4. Jumpiness, I am easily frightened by sudden sounds or sudden movements
5. The need to withdraw from others
6. Irritability, that is, I am easily agitated/annoyed and angry
7. Frequent mood swings
8. A bad conscience, blame myself, have guilt feelings
9. Fear of places and situations, which remind me of the intensive care unit
10. Muscular tension
11. Upsetting, unwanted thoughts or images of my time on the intensive care unit
12. Feeling numb (e.g. cannot cry, unable to have loving feelings)
13. Avoid places, people or situations that remind me of the intensive care unit
14. Feeling as if my plans or dreams for the future will not come true

En ce moment, je souffre de:

1. Troubles du sommeil
2. Cauchemars
3. Dépression (je me sens abattu(e), opprimé(e))
4. Nervosité (je suis facilement apeuré(e) par des bruits ou des mouvements brusques)
5. Besoin de m'isoler des autres personnes
6. Irritabilité (je suis facilement agité(e) et/ou agacé(e)
7. Changements fréquents d'humeur
8. Mauvaise conscience (je me blâme, j'ai des sentiments de culpabilité)
9. Peur de certains endroits ou de certaines situations
10. Tension musculaire
11. Tristesse, pensées ou images involontaires me rappelant mon séjour en réanimation
12. Sensation de lividité (je suis transi(e), ne peux pas pleurer ou incapable d'éprouver des sentiments d'amour)
13. Peur de certains endroits ou de certaines situations qui me rappellent la réanimation
14. Sensation que mes projet futurs ne se réaliseront pas

Chaque item est scoré de 1 (jamais) à 7 (toujours). Le score total est obtenu en additionnant les scores de chaque item, et peut varier de 14 à 48.

Hospital Anxiety and Depression Scale[16,17]

Hospital Anxiety and Depression Scale (HADS)

Tick the box beside the reply that is closest to how you have been feeling in the past week.
Don't take too long over you replies: your immediate is best.

D	A		D	A	
		**I feel tense or 'wound up':**			**I feel as if I am slowed down:**
	3	Most of the time	3		Nearly all the time
	2	A lot of the time	2		Very often
	1	From time to time, occasionally	1		Sometimes
	0	Not at all	0		Not at all
		**I still enjoy the things I used to enjoy:**			**I get a sort of frightened feeling like 'butterflies' in the stomach:**
0		Definitely as much		0	Not at all
1		Not quite so much		1	Occasionally
2		Only a little		2	Quite Often
3		Hardly at all		3	Very Often
		**I get a sort of frightened feeling as if something awful is about to happen:**			**I have lost interest in my appearance:**
	3	Very definitely and quite badly	3		Definitely
	2	Yes, but not too badly	2		I don't take as much care as I should
	1	A little, but it doesn't worry me	1		I may not take quite as much care
	0	Not at all	0		I take just as much care as ever
		**I can laugh and see the funny side of things:**			**I feel restless as I have to be on the move:**
0		As much as I always could		3	Very much indeed
1		Not quite so much now		2	Quite a lot
2		Definitely not so much now		1	Not very much
3		Not at all		0	Not at all
		**Worrying thoughts go through my mind:**			**I look forward with enjoyment to things:**
	3	A great deal of the time	0		As much as I ever did
	2	A lot of the time	1		Rather less than I used to
	1	From time to time, but not too often	2		Definitely less than I used to
	0	Only occasionally	3		Hardly at all
		**I feel cheerful:**			**I get sudden feelings of panic:**
3		Not at all		3	Very often indeed
2		Not often		2	Quite often
1		Sometimes		1	Not very often
0		Most of the time		0	Not at all
		**I can sit at ease and feel relaxed:**			**I can enjoy a good book or radio or TV program:**
	0	Definitely	0		Often
	1	Usually	1		Sometimes
	2	Not Often	2		Not often
	3	Not at all	3		Very seldom

Please check you have answered all the questions

Scoring:
Total score: Depression (D) ___________ Anxiety (A) _____________
0-7 = Normal
8-10 = Borderline abnormal (borderline case)
11-21 = Abnormal (case)

L'échelle HAD est un instrument qui permet de dépister les troubles anxieux et dépressifs. Elle comporte 14 items cotés de 0 à 3. Sept questions se rapportent à l'anxiété (total A) et sept autres à la dimension dépressive (total D), permettant ainsi l'obtention de deux scores (note maximale de chaque score = 21).

1. Je me sens tendu(e) ou énervé(e)
- La plupart du temps 3
- Souvent 2
- De temps en temps 1
- Jamais 0

2. Je prends plaisir aux mêmes choses qu'autrefois
- Oui, tout autant 0
- Pas autant 1
- Un peu seulement 2
- Presque plus 3

3. J'ai une sensation de peur comme si quelque chose d'horrible allait m'arriver
- Oui, très nettement 3
- Oui, mais ce n'est pas trop grave 2
- Un peu, mais cela ne m'inquiète pas 1
- Pas du tout 0

4. Je ris facilement et vois le bon côté des choses
- Autant que par le passé 0
- Plus autant qu'avant 1
- Vraiment moins qu'avant 2
- Plus du tout 3

5. Je me fais du souci
- Très souvent 3
- Assez souvent 2
- Occasionnellement 1
- Très occasionnellement 0

6. Je suis de bonne humeur
- Jamais 3
- Rarement 2
- Assez souvent 1
- La plupart du temps 0

7. Je peux rester tranquillement assis(e) à ne rien faire et me sentir décontracté(e)
- Oui, quoi qu'il arrive 0
- Oui, en général 1
- Rarement 2
- Jamais 3

8. J'ai l'impression de fonctionner au ralenti
- Presque toujours 3
- Très souvent 2
- Parfois 1
- Jamais 0

9. J'éprouve des sensations de peur et j'ai l'estomac noué
- Jamais 0
- Parfois 1
- Assez souvent 2
- Très souvent 3

10. Je ne m'intéresse plus à mon apparence
- Plus du tout 3
- Je n'y accorde pas autant d'attention que je devrais 2
- Il se peut que je n'y fasse plus autant attention 1
- J'y prête autant d'attention que par le passé 0

11. J'ai la bougeotte et n'arrive pas à tenir en place
- Oui, c'est tout à fait le cas 3
- Un peu 2
- Pas tellement 1
- Pas du tout 0

12. Je me réjouis d'avance à l'idée de faire certaines choses
- Autant qu'avant 0
- Un peu moins qu'avant 1
- Bien moins qu'avant 2
- Presque jamais 3

13. J'éprouve des sensations soudaines de panique
- Vraiment très souvent 3
- Assez souvent 2
- Pas très souvent 1
- Jamais 0

14. Je peux prendre plaisir à un bon livre ou à une bonne émission de radio ou de télévision
- Souvent 0
- Parfois 1
- Rarement 2
- Très rarement 3

Scores

Additionnez les points des réponses : 1, 3, 5, 7, 9, 11, 13 : Total A = _______

Additionnez les points des réponses : 2, 4, 6, 8, 10, 12, 14 : Total D = _______

Interprétation

Pour dépister des symptomatologies anxieuses et dépressives, l'interprétation suivante peut être proposée pour chacun des scores (A et D) :

- 7 ou moins : absence de symptomatologie
- 8 à 10 : symptomatologie douteuse – 11 et plus : symptomatologie certaine.

Cognitive function: the Alzheimer's Disease 8 (AD8)[18]

AD8 Dementia Screening Interview

Patient ID#:__________

CS ID#:___________
Date:___________

Remember, "Yes, a change" indicates that there has been a change in the last several years caused by cognitive (thinking and memory) problems.	YES, A change	NO, No change	N/A, Don't know
1. Problems with judgment (e.g., problems making decisions, bad financial decisions, problems with thinking)			
2. Less interest in hobbies/activities			
3. Repeats the same things over and over (questions, stories, or statements)			
4. Trouble learning how to use a tool, appliance, or gadget (e.g., VCR, computer, microwave, remote control)			
5. Forgets correct month or year			
6. Trouble handling complicated financial affairs (e.g., balancing checkbook, income taxes, paying bills)			
7. Trouble remembering appointments			
8. **Daily** problems with thinking and/or memory			

Avez-vous remarqué, au cours des dernières années, une des caractéristiques suivantes :	Oui, un changement	Non, aucun changement	Ne sais pas
1. Problèmes de jugement (p. ex. difficulté à prendre des décisions, mauvaises décisions financières, trouble de la pensée)	1	0	0
2. Moins d'intérêt pour ses loisirs	1	0	0
3. Se répète souvent (questions, histoires ou déclarations)	1	0	0
4. Difficulté à apprendre comment utiliser un nouvel appareil (p. ex. micro-ondes, ordinateur, magnétoscope)	1	0	0
5. Oubli du mois/année	1	0	0
6. Difficulté à gérer ses finances (p. ex. impôts, payer les comptes, vérifier les relevés de comptes bancaires)	1	0	0
7. Difficulté à se souvenir de ses rendez-vous	1	0	0
8. Problèmes quotidiens avec sa mémoire/raisonnement	1	0	0

Score total : somme des questions auxquelles il a été répondu « Oui, un changement » _______

Appendix N: Proposed sedation protocol for inhaled sedation with sevoflurane

PROTOCOLE DE SEDATION INHALEE PAR SEVOFLURANE (Système ANACONDA)

REGLES DE BASE
RASS <--> sevoflurane *versus* **BPS** <--> rémifentanil
Le BPS l'emporte sur le RASS (<--> d'abord le rémifentanil si nécessaire)

Au repos

1) En premier: si BPS ≥ 5 ou EVN ≥ 4 quel que soit le RASS

↓

***rémifentanil: augmenter la vitesse de 2 mL.h^{-1} toutes les 2 min jusqu'à BPS 3-4 (max 20 mL.h^{-1})**
(allo médecin si > 20 mL.h^{-1})

2) Si BPS 3-4 ou EVN <4 avec RASS

↓

RASS -5 inférieur à la cible

↓**sevoflurane** de 1 mL.h^{-1}

ET ↓ **rémifentanil** de 1 mL.h^{-1}

Réévaluer 5 minutes après

RASS cible

Stable puis diminution (paliers de 1 mL.h^{-1} de sevoflurane) pour rechercher la posologie minimale efficace / 4h maximum

RASS supérieur à la cible

Si RASS >+1, titrer bolus de sévoflurane 0.3 mL /2 min (max 1.2 mL) jusqu'à RASS cible

Puis ↑ sevoflurane de 1 mL.h^{-1}
Réévaluer 5 minutes après

(allo médecin si Fe sevo > 1.1)

Pour les soins

Augmenter la vitesse du rémifentanil 3 à 5 min avant (multiplier x 2, x 3 ou x 4 selon le soin)

AU COURS D'UN SOIN, si BPS ≥ 5
Arrêter le soin, multiplier la vitesse de rémifentanil par 2 et attendre 2 minutes

Pour les patients en VSAI
- Si FR ↘ 30%: passage en VAC
- Retour à la posologie initiale de rémifentanil à la fin du soin
- Retour en VSAI 2 minutes après

A TOUT MOMENT SI RASS ≥ 3:
Bolus de 0.6-1 mL de sévoflurane et allo médecin

Initiation de la sédation inhalée par sévoflurane

Amorçage de la tubulure de l'agent anesthésique

- Administrer un bolus d'1.5 mL (1.5 mL lors du premier raccordement du filtre AnaConDa, 1.2 mL lors du changement/remplacement d'un filtre déjà raccordé)
- Attendre l'affichage de la valeur CO_2 sur le moniteur de gaz
- Démarrez le pousse-seringue selon l'abaque ci-dessous (en fonction de la ventilation minute)

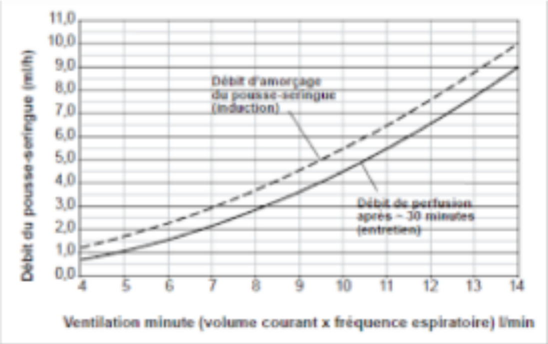

Proposition de cible initiale de Fe de sévoflurane en fonction du RASS souhaité

RASS	Fe
-1	0.1-0.3
-2	0.4-0.5
-3	0.6-0.8
-4	0.8-0.9
-5	0.9-1.1

Avis du Comité de protection des personnes Comité de protection des personnes Ile de France II sur Modification Substantielle

Informations CPP

Nom du CPP : Comité de protection des personnes Ile de France II
Adresse : Hôpital universitaire Necker Enfants malades Carré Necker - 149 rue de Sèvres - Porte N2 1er étage 75015 PARIS France
Courriel : cppidf2@gmail.com
Numéro de téléphone :

Informations promoteur

Organisme : CHU de Clermont-Ferrand
Nom et prénom : Morand Dominique

Investigateur

Investigateur : Jabaudon Matthieu

Informations dossier

Numéro SI : 18.00254.180921-MS05
Numéro national : 2018-000763-83
Référence interne : RBHP 2018 JABAUDON
Règlementation : Loi Jardé
Qualification de recherche : Catégorie 1
Produit ou acte : Médicaments à usage humain
Titre : Sédation par Sévoflurane dans le Syndrome de Détresse Respiratoire Aiguë - Etude prospective, randomisée et multicentrique
Numéro de la MS : 5
Motif de la MS : - mise à jour de la liste des centres et des investigateurs principaux,
- Mise à jour des actes liés à la recherche et des données recueillies

Récapitulatif des avis antérieurs

Dossier	Avis	Date d'émission
18.00254.180921	*Favorable*	18/06/2021
18.00254.180921-MS04	*Favorable*	24/08/2021

Ce dossier a été étudié en séance le 07/02/2022 12:45 et mandat a été donné au président du CPP d'émettre l'avis à réception des réponses du déposant aux dernières demandes. Au vu des réponses obtenues, l'avis suivant a donc été émis le 10/02/2022. C'est la date de la notification de l'avis sur le SI qui fait foi.

Avis favorable

Personnes ayant délibéré

**Collège**	**Catégorie**	**Nom et prénom**
Collège I	Qualification RIPH - Autre	COLONNA Pierre
Collège I	Spécialiste de médecine générale	MARTINEAUX Claude
Collège I	Qualification RIPH - Biostatistique ou épidémiologie	WACK Maxime
Collège I	Pharmacien hospitalier	BROISSAND Christine
Collège I	Qualification RIPH - Autre	GOUEL Aurélie
Collège I	Auxiliaire médical	SIMON Marie
Collège I	Qualification RIPH - Autre	FABRE Chantal
Collège I	Pharmacien hospitalier	STERNJACOB Thomas
Collège I	Spécialiste de médecine générale	JACOB VESTLING René
Collège I	Qualification RIPH - Biostatistique ou épidémiologie	BRESSON Jean-Louis
Collège I	Auxiliaire médical	QUIJOUX Flavien
Collège I	Qualification RIPH - Autre	MAMZER Marie-France
Collège II	Compétence en sciences humaines et sociales ou action sociale	BALLOUARD Christian
Collège II	Compétence en sciences humaines et sociales ou action sociale	GUEDMI Jeannette
Collège II	Compétence juridique	CHEVREAU Laura
Collège II	Représentant d'association agréée	ARDIOT Chantal
Collège II	Compétence éthique	DUPONT Jean-Claude
Collège II	Compétence juridique	LHUILLIER Floriane
Collège II	Représentant d'association agréée	SEHAN Monique
		VESTRIS Nora

Documents analysés par le CPP

**Catégorisation**	**Intitulé**	**Date de dépôt**
CVI - CV investigateurs	*2018-000763-83_Cv-Dr.Thouy_20220106.pdf*	07/01/2022
Courrier de demande de modification substantielle	*2018-000763-83_Courrier_20211117_SESAR.pdf*	02/12/2021
DOC - Autres documents	*2018-000763-83 FR 20211202 Form_eudract.pdf*	02/12/2021
DOC - Autres documents	*2018-000763-83_BPC-Dr.Thouy_20200109.pdf*	07/01/2022
Formulaire de demande de MS	*2018-000763-83_Form_dde_ansm_MS5_20211117_SESAR.pdf*	02/12/2021
LIS - Liste investigateurs	*2018-000763-83_Liste-Inv_v9_20211117_SESAR.pdf*	02/12/2021
Les informations justifiant le bien-fondé de chaque modification	*2018-000763-83_Justification_20211117_SESAR.pdf*	02/12/2021
PRO - Protocole	*2018-000763-83_Protocole_v9_20211117_SESAR.pdf*	02/12/2021
REP - Courrier de réponse	*2018-000763-83_Courrier_20220107_SESAR.pdf*	07/01/2022
RES - Résumé	*2018-000763-83_Summary_v9_20211117_SESAR.pdf*	02/12/2021
Tableau comparatif mettant en évidence les modifications apportées dans les documents de la demande initiale	*2018-000763-83_Tableau-Comparatif_20211117_SESAR.pdf*	02/12/2021

Tableau récapitulatif de l'ensemble des MS et MNS survenues depuis le DI	*2018-000763-83_Tableau_récapitulatif_MS_Sesar.pdf*	02/12/2021

Pr Marie-France MAMZER-BRUNEEL,

Présidente du CPP IDF II

CPP Ile de France 2
149, rue de Sèvres
75 743 Paris cedex 15
Carré Necker - Porte N2
Tél. : 01 42 19 26 88
Fax : 01 44 49 45 06
