# Supplementary material for: Design and Rationale of the Sevoflurane for Sedation in Acute Respiratory Distress Syndrome (SESAR) Randomized Controlled Trial
Source: J Clin Med. 2022 May 16;11(10):2796. doi: 10.3390/jcm11102796 (PMC9147018; doi:10.3390/jcm11102796)
Supplement: Supplementary file 1 [file jcm-11-02796-s001.zip › Supplementary Material File S3_Final_Conservative fluid management approach.pdf]

Supplementary Material File S3. Conservative fluid management approach.

A modified conservative fluid protocol will be used based on the findings from FACTT that conservative fluid management increased ventilator-free days. This protocol is recommended for all enrolled patients, to be used until study day 7, whichever occurs first.

1. Discontinue maintenance fluids.
2. Continue medications and nutrition.
3. Manage electrolytes and blood products per usual practice.
4. For shock, use any combination of fluid boluses[#] and vasopressor(s) to achieve MAP ≥60 mmHg as fast as possible. Wean vasopressors as quickly as tolerated beginning four hours after blood pressure has stabilized.
5. Withhold diuretic therapy in renal failure[§] and until 12 hours after last fluid bolus or vasopressor given.

This protocol is a simplified modification of the conservative protocol used in FACTT. For patients without a CVC, no fluid gain over the first 7 study days is recommended once patients' blood pressure has stabilized. Stable blood pressure is defined as no requirement for either vasopressors or a fluid bolus to support blood pressure for 12 or more hours.

<table>
<tr><th rowspan="2">CVP (recommended)</th><th rowspan="2">PAOP (optional)</th><th colspan="2">MAP ≥ 60 mm Hg AND off vasopressors for ≥ 12 hours</th></tr>
<tr><th>Average urine output < 0.5 ml/kg/hr</th><th>Average urine output ≥ 0.5 ml/kg/hr</th></tr>
<tr><td>>8</td><td>> 12</td><td>Furosemide*
Reassess in 1 hour</td><td rowspan="2">Furosemide*
Reassess in 4 hours</td></tr>
<tr><td>4-8</td><td>8-12</td><td rowspan="2">Give fluid bolus as fast as possible[#]
Reassess in 1 hour</td></tr>
<tr><td>< 4</td><td>< 8</td><td>No intervention
Reassess in 4 hours</td></tr>
</table>

[§] Renal failure is defined as dialysis dependence, oliguria with serum creatinine >3 mg/dL, or oliguria with serum creatinine 0-3 with urinary indices indicative of acute renal failure.

[#] Recommended fluid bolus = 15 mL/kg crystalloid (round to nearest 250 mL) or 1 Unit packed red cells or 25 grams albumin

* Recommended Furosemide dosing = begin with 20 mg bolus or 3 mg/hr infusion or last known effective dose. Double each subsequent dose until goal achieved (oliguria reversal or intravascular pressure target) or maximum infusion rate of 24 mg/hr or 160 mg bolus reached. Do not exceed 620 mg/day. Also, if the patient has heart failure, consider treatment with dobutamine.
