# Supplementary material for: Design and Rationale of the Sevoflurane for Sedation in Acute Respiratory Distress Syndrome (SESAR) Randomized Controlled Trial
Source: J Clin Med. 2022 May 16;11(10):2796. doi: 10.3390/jcm11102796 (PMC9147018; doi:10.3390/jcm11102796)
Supplement: Supplementary file 1 [file jcm-11-02796-s001.zip › Supplementary Material File S4_Final_Protocol for weaning from mechanical ventilation.pdf]

Supplementary Material File S4. Protocol for weaning from mechanical ventilation.

Commencement of weaning

Patients will be assessed for the following weaning readiness criteria each day between 06:00 and 10:00. If a patient procedure, test, or other extenuating circumstance prevents assessment for these criteria between 06:00 and 10:00, then the assessment and initiation of subsequent weaning procedures may be delayed for up to six hours.

Patients can be assessed for weaning readiness criteria twice a day:

1. At least 12 hours since enrollment in the trial
2. FiO_2 ≤0.50 and PEEP ≤8 cmH_2O
3. Values of both PEEP and FiO_2 ≤values from previous assessment
4. Systolic arterial pressure ≥90 mmHg without vasopressor support (≤0.5 µg/kg/min; dopamine will not be considered a vasopressor)

Spontaneous breathing trial (SBT) procedure and assessment for unassisted breathing

If criteria 1-4 above are met, first the neuromuscular blocking agent will need to be discontinued if the medication is still being infused. When the neuromuscular blocking agent has worn off and the patient is having spontaneous respirations, then initiate a trial of 60 minutes of spontaneous breathing with FiO_2 ≥0.5 using any of the following approaches:

1. Pressure support of 8-10 cmH_2O with PEEP = 0 cmH_2O
2. CPAP ≤5 cmH_2O
3. T-piece
4. Tracheostomy mask

Monitor for tolerance using the following:

1. SpO2 ≥90% and / or PaO2 ≥60 mmHg
2. Mean spontaneous tidal volume ≥4 mL/kg PBW (if measured)
3. Respiratory Rate ≤35 /min
4. pH ≥7.30 (if measured)
5. No respiratory distress (defined as 2 or more of the following):
    a. Heart rate ≥120% of the 06:00 rate (≤5 min at >120% may be tolerated)
    b. Marked use of accessory muscles
    c. Abdominal paradox
    d. Diaphoresis
    e. Marked subjective dyspnea.

If any of the goals 1-5 are not met, revert to previous ventilator settings or to the pressure support level needed to reach a Vt of 6 mL/kg of PBW, with PEEP and FiO_2 = previous settings and reassess for

weaning the next morning.

The clinical team may decide to change the mode of support during spontaneous breathing (PS = 5, CPAP, tracheostomy mask, or T-piece) at any time.

The AnaConDa-S will be removed from the breathing circuit as soon as inhaled sedation is interrupted.

The AnaConDa-S should be removed from the breathing circuit for spontaneous breathing trial.

Decision to remove ventilatory support

For intubated patients, if tolerance criteria for spontaneous breathing trial (1-5 above) are met for 60 minutes, the clinical team will decide to extubate.

If any of criteria 1-5 are not met during unassisted breathing, then the ventilator settings that were in use before the attempt to wean will be restored and the patient will be reassessed for weaning the following day.

Definition of unassisted breathing

a) Extubated with face mask, nasal prong oxygen, or room air, OR

b) T-tube breathing, OR

c) Tracheostomy mask breathing, OR

d) CPAP ≤ 5 without PS or IMV assistance

e) Use of CPAP or BIPAP solely for sleep apnea management

f) Use of a high flow oxygen system

For an uninterrupted period of at least 24 hours or more.

Completion of ventilator procedures

Patients will be considered to have completed the study ventilator procedures if any of the following conditions occur:

a. Death

b. Hospital discharge

c. Alive 28 days after enrollment

If a patient requires positive pressure ventilation after a period of unassisted breathing, the study ventilator procedures will resume unless the patient was discharged from the hospital or >28 days elapsed since enrollment.
