# Supplementary material for: Design and Rationale of the Sevoflurane for Sedation in Acute Respiratory Distress Syndrome (SESAR) Randomized Controlled Trial
Source: J Clin Med. 2022 May 16;11(10):2796. doi: 10.3390/jcm11102796 (PMC9147018; doi:10.3390/jcm11102796)
Supplement: Supplementary file 1 [file jcm-11-02796-s001.zip › Supplementary Material File S5_Final_Time-Events Schedule.pdf]

Supplementary Material File S5. Time-Events Schedule.

Measurement/Event	Day 0	1	2	3	4	5	6	7	14	28	90	365
Demographics, History and Physical	X											
Etiology of ARDS	X											
APACHE II, SAPS II	X											
HCG (females)	X											
Alcohol Use/Smoking History	X											
Study sevoflurane administration (maximum)	X	(X)	(X)	(X)	(X)	(X)	(X)	(X)				
Level of sedation	X	X	X	X	X	X	X	X				
Bispectral index value	A	A	A	A	A	A	A	A				
Sedation interruption (Y/N)	N	Y	Y	Y	Y	Y	Y	Y				
Ventilator parameters	X	X	X	X	X	X	X	X				
Hemodynamic parameters	X	X	X	X	X	X	X	X				
Arterial Blood Gases	X	X	X	A	A	A	A	A				
K^+, HCO_3^-, glucose	A	A	A	A	A	A	A	A				
Serum liver function tests (AST, ALT, bilirubin, INR)	X	X	X	X	X	X	X	X				
KDIGO criteria for acute kidney injury	X	X	X	X	X	X	X	X	X			
Pneumothorax (Y/N)	X	X	X	X	X	X	X	X				
Use of rescue procedures (Y/N)	X	X	X	X	X	X	X	X				
Central Venous Pressure	A	A	A	A	A	A	A	A				
On-study meds: sedative, opioid, corticosteroid administration (Y/N)	X	X	X	X	X	X	X	X				
SOFA Score (modified)[B]	X	X	X	X	X	X	X	X	X			
CAM-ICU	X	X	X	X	X	X	X					

Plasma Collection	X	X	X		X		X		X **#			
Urine Collection	X	X	X		X		X		X **#			
Whole Blood Collection for DNA/RNA studies	X		X									
Bronchoalveolar lavage		X			X							
Undiluted pulmonary edema fluid£	X	X										
Collection of heat moisture exchanger filters£		X										
Collection of AnaConDa-S filters*,£		X										
Number of ventilator-free days								X	X	X		
Disability (KATZ ADL)											X	X
Health-Related Quality of Life (SF-36)											X	X
Self-rated health											X	X
Pain-interference											X	X
Post-Traumatic Stress-like Symptoms (PTSS-14, HADS)											X	X
Cognitive Function (AD-8)											X	X
Subsequent return to work, hospital and ED use, and location of residence											X	X
Vital Status								X	X	X	X	X

X = Required
A = When available
* = in patients enrolled in the intervention arm
**# = or at ICU discharge, whichever occurs first
B = Records clinically available creatinine, platelets, bilirubin, SBP and vasopressor use
# = Measure during reference period (06:00-10:00); other values may be obtained closest to 08:00 on the specified calendar date
£ = in selected centers only, due to logistical considerations (n=30 patients from each group)
$ = in 5 selected centers only, due to logistical considerations (total n=25 patients)
