# Supplementary material for: Design and Rationale of the Sevoflurane for Sedation in Acute Respiratory Distress Syndrome (SESAR) Randomized Controlled Trial
Source: J Clin Med. 2022 May 16;11(10):2796. doi: 10.3390/jcm11102796 (PMC9147018; doi:10.3390/jcm11102796)
Supplement: Supplementary file 1 [file jcm-11-02796-s001.zip › Supplementary Material File S6_Final_Instruments and questionnaires.pdf]

Supplementary Material File S6. Instruments and questionnaires for ICU-acquired delirium and long-term outcome assessment.

A. Confusion Assessment Method for the ICU (CAM-ICU)

Instructions: To evaluate for the presence of delirium in your patient, complete this clinical assessment every shift (8-12 hours).

CAM-ICU is a valid and reliable delirium assessment tool recommended by the Society of Critical Care Medicine (SCCM) in its 2013 Pain, Agitation, and Delirium (PAD) guidelines.

<table>
<tr><th>CAM-ICU</th><th>Criteria</th><th>✓ Present</th></tr>
<tr><td colspan="3">FEATURE 1: Alteration/Fluctuation in Mental Status</td></tr>
<tr><td>▪ Is the patient's mental status different than his/her baseline? OR
▪ Has the patient had any fluctuation in mental status in the past 24 hours as evidenced by fluctuation on a sedation scale (eg, RASS, Glasgow Coma Scale [GCS]), or previous delirium assessment?</td><td>If Yes for either question ▶</td><td>☐</td></tr>
<tr><td colspan="3">FEATURE 2: Inattention 1: Alteration/Fluctuation in Mental Status</td></tr>
<tr><td>Letters Attention Test:
Tell the patient "I am going to read to you a series of 10 letters. Whenever you hear the letter 'A,' squeeze my hand."
S A V E A H A A R T
Count errors (each time patient fails to squeeze on the letter "A" and squeezes on a letter other than "A").</td><td>If number of errors >2 ▶</td><td>☐</td></tr>
<tr><td colspan="3">FEATURE 3: Altered Level of Consciousness (LOC)</td></tr>
<tr><td>▪ Present if the RASS score is anything <u>other than</u> Alert and Calm (zero) OR
▪ If SAS is anything <u>other than</u> Calm (4)</td><td>If RASS ≠0 OR SAS ≠4 ▶</td><td>☐</td></tr>
<tr><td colspan="3">FEATURE 4: Disorganized Thinking</td></tr>
<tr><td>Yes/No Questions: Ask the patient to respond:
1. Will a stone float on water?
2. Are there fish in the sea?
3. Does 1 pound weigh more than 2 pounds?
4. Can you use a hammer to pound a nail?
Count errors (each time patient answers incorrectly).
Commands: Ask the patient to follow your instructions:
a) "Hold up this many fingers." (Hold 2 fingers in front of the patient.)
b) "Now do the same thing with the other hand." (Do <u>not</u> demonstrate the number of fingers this time.)
▫ If unable to move both arms, for part "b" of command ask patient to "Hold up one more finger."
Count errors if patient is unable to complete the entire command.</td><td>If combined number of errors >1 ▶</td><td></td></tr>
<tr><td>If Features 1 and 2 are both present <u>and</u> either Features 3 <u>or</u> 4 are present:
CAM-ICU is positive, delirium is present</td><td colspan="2">Delirium present ☐
Delirium absent ☐</td></tr>
</table>

Le score peut être utilisé uniquement chez les patients capables d'ouvrir les yeux et de serrer la main à la demande

Critère 1 : Début soudain et évolution fluctuante

- La modification de l'état mental du patient est-elle soudaine ? (oui = positif/non = négatif)
- Y-a-t-il des fluctuations sur les dernières 24 heures ? (oui = positif/non = négatif)

Critère 2 : Inattention

- Le patient présente-t-il des difficultés à focaliser son attention ?
  - Test «ABRACADABRA» : Epelez chaque lettre (1-2 secondes entre chaque lettre) et demandez au patient de serrer la main sur la lettre «A» :

Le test est positif s'il y a plus de deux erreurs (soit le patient ne sert pas la main sur la lettre A, soit il la sert sur une autre lettre)

Critère 3 : Désorganisation de la pensée

- Question oui/non :
  - Est-ce qu'une pierre flotte sur l'eau ?
  - Y-a-t-il des poissons dans la mer ?
  - Est-ce qu'un kilogramme pèse plus que deux kilogrammes ?
  - Peut-on utiliser un marteau pour enfoncer un clou ?

Le test est positif s'il y a plus d'une réponse fausse

- Commandes verbales :
  - Dites au patient : «Montrez-moi le même nombre de doigts» (Montrez-lui deux doigts)
  - Puis «Faites la même chose avec l'autre main»

Le test est positif si le patient se trompe dans ces deux commandes

Etape 4 : Evaluation de la vigilance

- Globalement, comment évalueriez-vous l'état général de votre patient ? Alerte, vigile, léthargique, stuporeux, comateux

Le test est positif si le niveau de conscience est autre qu'alerte

Le diagnostic de delirium requiert la présence de 3 des 4 critères. Les critères 1 et 2 sont toujours requis, accompagnés du critère 3 ou 4

B. Katz Activities of Daily Living (ADL)

Katz Index of Independence in Activities of Daily Living		
**Activities**<br>Points (1 or 0)	**Independence**<br>(1 Point)<br><br>**NO** supervision, direction or personal assistance.	**Dependence**<br>(0 Points)<br><br>**WITH** supervision, direction, personal assistance or total care.
**BATHING**<br><br>Points: __________	**(1 POINT)** Bathes self completely or needs help in bathing only a single part of the body such as the back, genital area or disabled extremity.	**(0 POINTS)** Need help with bathing more than one part of the body, getting in or out of the tub or shower. Requires total bathing
**DRESSING**<br><br>Points: __________	**(1 POINT)** Get clothes from closets and drawers and puts on clothes and outer garments complete with fasteners. May have help tying shoes.	**(0 POINTS)** Needs help with dressing self or needs to be completely dressed.
**TOILETING**<br><br>Points: __________	**(1 POINT)** Goes to toilet, gets on and off, arranges clothes, cleans genital area without help.	**(0 POINTS)** Needs help transferring to the toilet, cleaning self or uses bedpan or commode.
**TRANSFERRING**<br><br>Points: __________	**(1 POINT)** Moves in and out of bed or chair unassisted. Mechanical transfer aids are acceptable	**(0 POINTS)** Needs help in moving from bed to chair or requires a complete transfer.
**CONTINENCE**<br><br>Points: __________	**(1 POINT)** Exercises complete self control over urination and defecation.	**(0 POINTS)** Is partially or totally incontinent of bowel or bladder
**FEEDING**<br><br>Points: __________	**(1 POINT)** Gets food from plate into mouth without help. Preparation of food may be done by another person.	**(0 POINTS)** Needs partial or total help with feeding or requires parenteral feeding.
**TOTAL POINTS:** ________	**SCORING:** 6 = High (*patient independent*)	0 = Low (*patient very dependent*

QUESTIONNAIRE Katz ADL

Cochez la réponse de votre choix, une par ligne

	Autonome	Besoin d'aide	Dépendant(e)
**Hygiène corporelle**	❑	❑	❑
	Autonome pour le choix des vêtements et l'habillage	Autonome pour le choix des vêtements, l'habillage mais besoin d'aide pour se chausser	Dépendant(e)
**Habillage**	❑	❑	❑
	Autonome pour aller aux toilettes, se déshabiller et se rhabiller ensuite	Je dois être accompagné(e) ou besoin d'aide pour se déshabiller ou se rhabiller	Je ne peux pas aller aux toilettes seul(e)
**Aller aux toilettes**	❑	❑	❑
	Autonome	J'ai besoin d'aide	Je suis grabataire
**Locomotion**	❑	❑	❑
	Continent(e)	Incontinence occasionnelle	Incontinent(e)
**Continence**	❑	❑	❑
	Je mange seul(e)	Je suis aidé(e) pour couper la viande et peler les fruits	Dépendant(e)
**Repas**	❑	❑	❑

C. Short Form-36 (SF-36)

SF-36 QUESTIONNAIRE

Name:__________________ Ref. Dr:__________________ Date: _______

ID#: ______________ Age: _______ Gender: M / F

Please answer the 36 questions of the Health Survey completely, honestly, and without interruptions.

GENERAL HEALTH:

In general, would you say your health is:

◯ Excellent ◯ Very Good ◯ Good ◯ Fair ◯ Poor

Compared to one year ago, how would you rate your health in general now?

◯ Much better now than one year ago
◯ Somewhat better now than one year ago
◯ About the same
◯ Somewhat worse now than one year ago
◯ Much worse than one year ago

LIMITATIONS OF ACTIVITIES:

The following items are about activities you might do during a typical day. Does your health now limit you in these activities? If so, how much?

Vigorous activities, such as running, lifting heavy objects, participating in strenuous sports.

◯ Yes, Limited a lot ◯ Yes, Limited a Little ◯ No, Not Limited at all

Moderate activities, such as moving a table, pushing a vacuum cleaner, bowling, or playing golf

◯ Yes, Limited a Lot ◯ Yes, Limited a Little ◯ No, Not Limited at all

Lifting or carrying groceries

◯ Yes, Limited a Lot ◯ Yes, Limited a Little ◯ No, Not Limited at all

Climbing several flights of stairs

◯ Yes, Limited a Lot ◯ Yes, Limited a Little ◯ No, Not Limited at all

Climbing one flight of stairs

◯ Yes, Limited a Lot ◯ Yes, Limited a Little ◯ No, Not Limited at all

Bending, kneeling, or stooping

◯ Yes, Limited a Lot ◯ Yes, Limited a Little ◯ No, Not Limited at all

Walking more than a mile

◯ Yes, Limited a Lot ◯ Yes, Limited a Little ◯ No, Not Limited at all

Walking several blocks

◯ Yes, Limited a Lot ◯ Yes, Limited a Little ◯ No, Not Limited at all

Walking one block

◯ Yes, Limited a Lot ◯ Yes, Limited a Little ◯ No, Not Limited at all

Bathing or dressing yourself
◯Yes, Limited a Lot ◯Yes, Limited a Little ◯No, Not Limited at all

PHYSICAL HEALTH PROBLEMS:
During the past 4 weeks, have you had any of the following problems with your work or other regular daily activities as a result of your physical health?

Cut down the amount of time you spent on work or other activities
◯Yes ◯No

Accomplished less than you would like
◯Yes ◯No

Were limited in the kind of work or other activities
◯Yes ◯No

Had difficulty performing the work or other activities (for example, it took extra effort)
◯Yes ◯No

EMOTIONAL HEALTH PROBLEMS:
During the past 4 weeks, have you had any of the following problems with your work or other regular daily activities as a result of any emotional problems (such as feeling depressed or anxious)?

Cut down the amount of time you spent on work or other activities
◯Yes ◯No

Accomplished less than you would like
◯Yes ◯No

Didn't do work or other activities as carefully as usual
◯Yes ◯No

SOCIAL ACTIVITIES:
Emotional problems interfered with your normal social activities with family, friends, neighbors, or groups?

◯Not at all ◯Slightly ◯Moderately ◯Severe ◯Very Severe

PAIN:
How much bodily pain have you had during the past 4 weeks?

◯None ◯Very Mild ◯Mild ◯Moderate ◯Severe ◯Very Severe

During the past 4 weeks, how much did pain interfere with your normal work (including both work outside the home and housework)?

◯Not at all ◯A little bit ◯Moderately ◯Quite a bit ◯Extremely

ENERGY AND EMOTIONS:
These questions are about how you feel and how things have been with you during the last 4 weeks. For each question, please give the answer that comes closest to the way you have been feeling.

Did you feel full of pep?
- All of the time
- Most of the time
- A good Bit of the Time
- Some of the time
- A little bit of the time
- None of the Time

Have you been a very nervous person?
- All of the time
- Most of the time
- A good Bit of the Time
- Some of the time
- A little bit of the time
- None of the Time

Have you felt so down in the dumps that nothing could cheer you up?
- All of the time
- Most of the time
- A good Bit of the Time
- Some of the time
- A little bit of the time
- None of the Time

Have you felt calm and peaceful?
- All of the time
- Most of the time
- A good Bit of the Time
- Some of the time
- A little bit of the time
- None of the Time

Did you have a lot of energy?
- All of the time
- Most of the time
- A good Bit of the Time
- Some of the time
- A little bit of the time
- None of the Time

Have you felt downhearted and blue?

- All of the time
- Most of the time
- A good Bit of the Time
- Some of the time
- A little bit of the time
- None of the Time

Did you feel worn out?

- All of the time
- Most of the time
- A good Bit of the Time
- Some of the time
- A little bit of the time
- None of the Time

Have you been a happy person?

- All of the time
- Most of the time
- A good Bit of the Time
- Some of the time
- A little bit of the time
- None of the Time

Did you feel tired?

- All of the time
- Most of the time
- A good Bit of the Time
- Some of the time
- A little bit of the time
- None of the Time

SOCIAL ACTIVITIES:

During the past 4 weeks, how much of the time has your physical health or emotional problems interfered with your social activities (like visiting with friends, relatives, etc.)?

- All of the time
- Most of the time
- Some of the time
- A little bit of the time
- None of the Time

QUESTIONNAIRE SF-36

1. Dans l'ensemble, pensez-vous que votre santé est :

Excellente	Très bonne	Bonne	Médiocre	Mauvaise
❑	❑	❑	❑	❑

2. Par rapport à l'année dernière à la même époque, comment trouvez-vous votre état de santé en ce moment ?

Bien meilleur que l'an dernier	Plutôt meilleur	A peu près pareil	Plutôt moins bon	Beaucoup moins bon
❑	❑	❑	❑	❑

3. Voici une liste d'activités que vous pouvez avoir à faire dans votre vie de tous les jours. Pour chacune d'entre elles indiquez si vous êtes limité(e) en raison de votre état de santé actuel.

Cochez la réponse de votre choix, une par ligne

	**Oui, très limité**	**Oui, plutôt limité**	**Non, pas du tout limité**
**Efforts physiques importants** (courir, soulever un objet lourd, faire du sport)	❑	❑	❑
**Efforts physiques modérés** (déplacer une table, passer l'aspirateur, jouer aux boules)	❑	❑	❑
Soulever et porter les courses	❑	❑	❑
Monter **plusieurs étages** par l'escalier	❑	❑	❑
Monter **un étage** par l'escalier	❑	❑	❑
Se pencher en avant, se mettre à genoux, s'accroupir	❑	❑	❑
Marcher **plus d'un km** à pied	❑	❑	❑
Marcher **plusieurs centaines de mètres**	❑	❑	❑
Marcher **une centaine de mètres**	❑	❑	❑
Prendre un bain, une douche ou s'habiller	❑	❑	❑

4. Au cours de ces 4 dernières semaines, et en raison de votre état physique.

Cochez la réponse de votre choix, une par ligne

	OUI	NON
Avez-vous réduit **le temps passé** à votre travail ou à vos activités habituelles ?	❑	❑
Avez-vous **accompli moins** de choses que ce que vous auriez souhaité ?	❑	❑
Avez-vous dû arrêter de faire **certaines** choses ?	❑	❑
Avez-vous eu des **difficultés** à faire votre travail ou toute autre activité ? (par ex : cela vous a demandé un effort supplémentaire)	❑	❑

5. Au cours de ces 4 dernières semaines, et en raison de votre état émotionnel (comme vous sentir triste, nerveux(se), ou déprimé(e).

Cochez la réponse de votre choix, une par ligne

	OUI	NON
Avez-vous réduit **le temps passé** à votre travail ou à vos activités habituelles ?	❑	❑
Avez-vous **accompli moins** de choses que ce que vous auriez souhaité ?	❑	❑
Avez-vous eu des **difficultés** à faire ce que vous aviez à faire **avec autant de soin et d'attention que d'habitude** ?	❑	❑

6. Au cours de ces 4 dernières semaines dans quelle mesure votre état de santé, physique ou émotionnel, vous a-t-il gêné(e) dans votre vie sociale et vos relations avec les autres, votre famille, vos amis, vos connaissances ?

Pas du tout	Un petit peu	Moyennement	Beaucoup	Enormément
❑	❑	❑	❑	❑

7. Au cours de ces 4 dernières semaines, quelle a été l'intensité de vos douleurs physiques ?

Nulle	Très faible	Faible	Moyenne	Grande	Très grande
❑	❑	❑	❑	❑	❑

8. Au cours de ces 4 dernières semaines, dans quelle mesure vos douleurs physiques vous ont-elles limité(e) dans votre travail ou vos activités domestiques ?

Pas du tout	Un petit peu	Moyennement	Beaucoup	Enormément
❑	❑	❑	❑	❑

9. Les questions qui suivent portent sur comment vous vous êtes senti(e) au cours de ces 4 dernières semaines. Pour chaque question, veuillez indiquer la réponse qui vous semble la plus appropriée. Au cours de ces 4 dernières semaines, y a-t-il eu des moments où :

Cochez la réponse de votre choix, une par ligne

	en permanence	très souvent	souvent	quelque fois	rarement	jamais
Vous vous êtes senti(e) dynamique ?	❑	❑	❑	❑	❑	❑
Vous vous êtes senti(e) nerveux ?	❑	❑	❑	❑	❑	❑
Vous vous êtes senti(e) si découragé(e) que rien ne pouvait vous remonter le moral ?	❑	❑	❑	❑	❑	❑
Vous vous êtes senti(e) calme et détendu(e) ?	❑	❑	❑	❑	❑	❑
Vous vous êtes senti(e) débordant(e) d'énergie ?	❑	❑	❑	❑	❑	❑
Vous vous êtes senti(e) triste et abattu(e) ?	❑	❑	❑	❑	❑	❑
Vous vous êtes senti(e) épuisé(e) ?	❑	❑	❑	❑	❑	❑
Vous vous êtes senti(e) heureux ?	❑	❑	❑	❑	❑	❑
Vous vous êtes senti(e) fatigué(e) ?	❑	❑	❑	❑	❑	❑

10. Au cours de ces 4 dernières semaines y a-t-il eu des moments où votre état de santé, physique ou émotionnel, vous a gêné(e) dans votre vie sociale et vos relations avec les autres, votre famille, vos amis, vos connaissances ?

En permanence	Une bonne partie du temps	De temps en temps	Rarement	Jamais
❑	❑	❑	❑	❑

11. Indiquez, pour chacune des phrases suivantes, dans quelle mesure elles sont vraies ou fausses dans votre cas :

Cochez la réponse de votre choix, une par ligne

	**totalement vraie**	**plutôt vraie**	**je ne sais pas**	**plutôt fausse**	**totalement fausse**
Je tombe malade plus facilement que les autres	❑	❑	❑	❑	❑
Je me porte aussi bien que n'importe qui	❑	❑	❑	❑	❑
Je m'attends à ce que ma santé se dégrade	❑	❑	❑	❑	❑
Je suis en excellente santé	❑	❑	❑	❑	❑

D. Self-rated Health

In general, would you say that your ***health*** is

- Excellent?
- Very good?
- Good?
- Fair?
- Poor?

AUTO-EVALUATION SUR L'ETAT DE SANTE

D'une manière générale, diriez-vous que votre état de santé est :

Excellent	Très bon	Bon	Correct	Mauvaise
❑	❑	❑	❑	❑

E. Pain Interference

In the past 7 days, how much did pain interfere with your day to day activities ?

- Not at all
- A little bit
- Somewhat
- Quite a bit
- Very much

RETENTISSEMENT DE LA DOULEUR DANS LES ACTIVITES QUOTIDIENNES

Au cours des 7 derniers jours, à quel point la douleur a-t-elle interféré avec vos activités quotidiennes ?

Pas du tout	Très peu	Quelque peu	Modérément	Beaucoup
❑	❑	❑	❑	❑

F. Post-Traumatic Stress Symptoms (PTSS)-14

The UK-PTSS-14.

Presently (this means in the past few days) I suffer from:

1. Sleep problems
2. Nightmares
3. Depression, I feel dejected/downtrodden
4. Jumpiness, I am easily frightened by sudden sounds or sudden movements
5. The need to withdraw from others
6. Irritability, that is, I am easily agitated/annoyed and angry
7. Frequent mood swings
8. A bad conscience, blame myself, have guilt feelings
9. Fear of places and situations, which remind me of the intensive care unit
10. Muscular tension
11. Upsetting, unwanted thoughts or images of my time on the intensive care unit
12. Feeling numb (e.g. cannot cry, unable to have loving feelings)
13. Avoid places, people or situations that remind me of the intensive care unit
14. Feeling as if my plans or dreams for the future will not come true

QUESTIONNAIRE PTSS-14

Entourez la réponse de votre choix, une par ligne

En ce moment, je souffre de :

	jamais 1	2	3	4	5	6	toujours 7
Troubles du sommeil	1	2	3	4	5	6	7
Cauchemars	1	2	3	4	5	6	7
Dépression (je me sens abattu(e), opprimé(e))	1	2	3	4	5	6	7
Nervosité (je suis facilement apeuré(e) par des bruits ou des mouvements brusques)	1	2	3	4	5	6	7
Besoin de m'isoler des autres personnes	1	2	3	4	5	6	7
Irritabilité (je suis facilement agité(e) et/ou agacé(e)	1	2	3	4	5	6	7
Changements fréquents d'humeur	1	2	3	4	5	6	7
Mauvaise conscience (je me blâme, j'ai des sentiments de culpabilité)	1	2	3	4	5	6	7
Peur de certains endroits ou de certaines situations	1	2	3	4	5	6	7
Tension musculaire	1	2	3	4	5	6	7
Tristesse, pensées ou images involontaires me rappelant mon séjour en réanimation	1	2	3	4	5	6	7
Sensation de lividité (je suis transi(e), ne peux pas pleurer ou incapable d'éprouver des sentiments d'amour)	1	2	3	4	5	6	7
Peur de certains endroits ou de certaines situations qui me rappellent la réanimation	1	2	3	4	5	6	7
Sensation que mes projets futurs ne se réaliseront pas	1	2	3	4	5	6	7

(Each item row is printed with the labels "jamais" above 1 and "toujours" above 7.)

G. Hospital Anxiety and Depression Scale

Hospital Anxiety and Depression Scale (HADS)

Tick the box beside the reply that is closest to how you have been feeling in the past week.
Don't take too long over you replies: your immediate is best.

D	A		D	A	
		**I feel tense or 'wound up':**			**I feel as if I am slowed down:**
	3	Most of the time	3		Nearly all the time
	2	A lot of the time	2		Very often
	1	From time to time, occasionally	1		Sometimes
	0	Not at all	0		Not at all
		**I still enjoy the things I used to enjoy:**			**I get a sort of frightened feeling like 'butterflies' in the stomach:**
0		Definitely as much		0	Not at all
1		Not quite so much		1	Occasionally
2		Only a little		2	Quite Often
3		Hardly at all		3	Very Often
		**I get a sort of frightened feeling as if something awful is about to happen:**			**I have lost interest in my appearance:**
	3	Very definitely and quite badly	3		Definitely
	2	Yes, but not too badly	2		I don't take as much care as I should
	1	A little, but it doesn't worry me	1		I may not take quite as much care
	0	Not at all	0		I take just as much care as ever
		**I can laugh and see the funny side of things:**			**I feel restless as I have to be on the move:**
0		As much as I always could		3	Very much indeed
1		Not quite so much now		2	Quite a lot
2		Definitely not so much now		1	Not very much
3		Not at all		0	Not at all
		**Worrying thoughts go through my mind:**			**I look forward with enjoyment to things:**
	3	A great deal of the time	0		As much as I ever did
	2	A lot of the time	1		Rather less than I used to
	1	From time to time, but not too often	2		Definitely less than I used to
	0	Only occasionally	3		Hardly at all
		**I feel cheerful:**			**I get sudden feelings of panic:**
3		Not at all		3	Very often indeed
2		Not often		2	Quite often
1		Sometimes		1	Not very often
0		Most of the time		0	Not at all
		**I can sit at ease and feel relaxed:**			**I can enjoy a good book or radio or TV program:**
	0	Definitely	0		Often
	1	Usually	1		Sometimes
	2	Not Often	2		Not often
	3	Not at all	3		Very seldom

Please check you have answered all the questions

Scoring:
Total score: Depression (D) ___________ Anxiety (A) ______________
0-7 = Normal
8-10 = Borderline abnormal (borderline case)
11-21 = Abnormal (case)

ECHELLE HAD

1. Je me sens tendu(e) ou énervé(e).

Jamais	De temps en temps	Souvent	La plupart du temps
◻	◻	◻	◻

2. Je prends plaisir aux mêmes choses qu'autrefois.

Oui, tout autant	Pas autant	Un peu seulement	Presque plus
◻	◻	◻	◻

3. J'ai une sensation de peur comme si quelque chose d'horrible allait m'arriver.

Pas du tout	Un peu mais cela ne m'inquiète pas	Oui, mais ce n'est pas trop grave	Oui, très nettement
◻	◻	◻	◻

4. Je ris facilement et vois le bon côté des choses.

Autant que par le passé	Plus autant qu'avant	Vraiment moins qu'avant	Plus du tout
◻	◻	◻	◻

5. Je me fais du souci.

Très occasionnellement	Occasionnellement	Assez souvent	Très souvent
◻	◻	◻	◻

6. Je suis de bonne humeur.

La plupart du temps	Assez souvent	Rarement	Jamais
◻	◻	◻	◻

7. Je peux rester tranquillement assis à ne rien faire et me sentir décontracté.

Oui, quoi qu'il arrive	Oui, en général	Rarement	Jamais
◻	◻	◻	◻

8. J'ai l'impression de fonctionner au ralenti.

Jamais	Parfois	Très souvent	Presque toujours
◻	◻	◻	◻

9. J'éprouve des sensations de peur et j'ai l'estomac noué.

Jamais	Parfois	Assez souvent	Très souvent
☐	☐	☐	☐

10. Je ne m'intéresse plus à mon apparence.

J'y prête autant d'attention que par le passé	Il se peut que je n'y fasse plus autant attention	Je n'y accorde pas autant d'attention que je devrais	Plus du tout
☐	☐	☐	☐

11. J'ai la bougeotte et n'arrive pas à tenir en place.

Pas du tout	Pas tellement	Un peu	Oui, c'est tout à fait le cas
☐	☐	☐	☐

12. Je me réjouis d'avance à l'idée de faire certaines choses.

Autant qu'avant	Un peu moins qu'avant	Bien moins qu'avant	Presque jamais
☐	☐	☐	☐

13. J'éprouve des sensations soudaines de panique.

Jamais	Pas très souvent	Assez souvent	Vraiment très souvent
☐	☐	☐	☐

14. Je peux prendre plaisir à un bon livre ou à une bonne émission de radio ou télévision.

Souvent	Parfois	Rarement	Très rarement
☐	☐	☐	☐

H. Alzheimer's Disease 8 (AD8)

AD8 Dementia Screening Interview

Patient ID#:__________

CS ID#:____________

Date:____________

Remember, "Yes, a change" indicates that there has been a change in the last several years caused by cognitive (thinking and memory) problems.	**YES, A change**	**NO, No change**	**N/A, Don't know**
1. Problems with judgment (e.g., problems making decisions, bad financial decisions, problems with thinking)			
2. Less interest in hobbies/activities			
3. Repeats the same things over and over (questions, stories, or statements)			
4. Trouble learning how to use a tool, appliance, or gadget (e.g., VCR, computer, microwave, remote control)			
5. Forgets correct month or year			
6. Trouble handling complicated financial affairs (e.g., balancing checkbook, income taxes, paying bills)			
7. Trouble remembering appointments			
8. **Daily** problems with thinking and/or memory			

QUESTIONNAIRE AD-8

Cochez la réponse de votre choix, une par ligne

	Oui, un changement	Non, aucun changement	Je ne sais pas
Avez-vous remarqué, au cours des dernières années, une des caractéristiques suivantes :	❑	❑	❑
Problèmes de jugement (par ex. : difficulté à prendre des décisions, mauvaises décisions financières, trouble de la pensée)	❑	❑	❑
Moins d'intérêt pour ses loisirs	❑	❑	❑
Se répète souvent (questions, histoires ou déclarations)	❑	❑	❑
Difficulté à apprendre comment utiliser un nouvel appareil (par ex. : micro-ondes, ordinateur, magnétoscope)	❑	❑	❑
Oubli du mois/année	❑	❑	❑
Difficulté à gérer ses finances (par ex. : impôts, payer les comptes, vérifier les relevés de comptes bancaires)	❑	❑	❑
Difficulté à se souvenir de ses rendez-vous	❑	❑	❑
Problèmes quotidiens avec sa mémoire/raisonnement	❑	❑	❑
