# Supplementary material for: Design and Rationale of the Sevoflurane for Sedation in Acute Respiratory Distress Syndrome (SESAR) Randomized Controlled Trial
Source: J Clin Med. 2022 May 16;11(10):2796. doi: 10.3390/jcm11102796 (PMC9147018; doi:10.3390/jcm11102796)
Supplement: Supplementary file 1 [file jcm-11-02796-s001.zip › Supplementary Material File S7_Final_Statistical Analysis Plan.pdf]

Supplementary Material File S7. Statistical Analysis Plan.

All analyses will be performed with Stata software version 15 (StataCorp, College Station, USA), R version 4.0.5 (R Foundation for Statistical Computing, Vienna, Austria), and MPlus Version 8.6 (Muthen & Muthen) before the breaking of the randomization code, according to International Conference on Harmonization-Good Clinical Practice guidelines [1]. A two-sided P value of less than 0.05 will be considered for statistical significance of all analyses (except for interim analysis). Because of the potential for type I error due to multiple comparisons, findings for analyses of secondary endpoints will be interpreted as exploratory and systematic correction of type I error will not be applied; analyses will focus not only on statistical significance but also on the magnitude of differences [2]. The primary analysis will be performed using an intention-to-treat (ITT) principle. Then, we will perform per-protocol and subgroup analyses on the primary and secondary outcomes.

The primary endpoint (ventilator-free days at day 28, VFD28) will be analyzed using a mixture of generalized gamma distributions to concatenate the overall frequency and distribution of the times [3]. Multivariable adjustments using generalized gamma distribution mixed models will be performed in 1) a first model including only the randomization-stratification variables (severe ARDS, COVID-19, and shock at enrollment) and center as random-effect (to measure between- and within-center variability); 2) a second model with covariates from the first model and covariates with clinically relevant relationships with the outcome and significant in univariate analyses (P <0.10). Results will be expressed as regression coefficients and 95% confidence intervals.

The key secondary outcome of day-90 survival will be evaluated using Kaplan-Meier approach and compared using log-rank test (univariate analysis) and marginal Cox proportional hazard regression (multivariable analysis); adjusted analyses will be conducted using the same two models described above. Results will be expressed as hazard-ratios with 95% confidence intervals and proportional-hazard assumption will be verified using the Schoenfeld test and by plotting residuals.

Categorical variables will be analyzed using chi-squared test or Fisher's exact test, as appropriate. For multivariable analysis, adjusted analyses will be performed with the use of random-effects robust Poisson generalized linear model (Stata commands glm, link=log and offset) to consider within- and between-center variability with center as random-effect. Results will be expressed as Relative Risks and 95% confidence intervals. Adjusted analyses will be conducted using the same adjustment variables described for the first model of the primary endpoint analysis.

Continuous parameters outcomes will be compared between groups using Student's t-test or Mann-Whitney U test. Normality will be studied by the Shapiro-Wilk test and homoscedasticity using the Fisher-Snedecor test. Multivariable analyses will be performed with linear mixed models, using the same adjustment variables described for the first model of the primary endpoint analysis. We will minimize the Akaike Information Criterion to determine the adequate relationship (logarithm, square

root, linear, quadratic, or cubic polynomial, or terciles or quartiles categorization). Results will be expressed as regression coefficients and 95% confidence intervals. For variables such as organ failure-free, ICU-free or hospital-free days, generalized linear models will initially use Poisson distribution or, alternatively, negative binomial distribution. When assumptions for these distributions are not met, we will analyze data using the nonparametric Van Elteren test, adjusted only for the center. Longitudinal analysis of repeated data will be performed using mixed models to study fixed effects group, time-point evaluation, and their interaction considering between- and within-subject variability. An ancillary analysis will also be conducted to assess the presence of phenotypes among patients with ARDS, based on distinct clinical, imaging [4–6], and/or biological [7,8] profiles, and their differential therapeutic response to sevoflurane, if any, using multidimensional analyses such as factorial analysis and latent-class analysis.

A learning curve analysis (sensitivity analysis excluding the five first patients of each center for the primary outcome) will be performed to evaluate if an improvement in terms of primary outcome is observed over time, in other words to assess whether the study results might be associated with some degree of "learning effect" attributable to the specific training on inhaled sedation.

The nature of missing data (missing at random or not) will be studied through sensitivity analysis and the most appropriate approach to imputing missing data will be proposed accordingly. We do not expect missing data for the primary outcome measure and only complete case-analysis will be performed. For secondary outcomes, a complete-case analysis will initially be performed; if the frequency of missing data is >5%, an additional analysis will be performed using the multiple imputation method (Stata command mi).

REFERENCES

1. ICH Harmonised Tripartite Guideline: Guideline for Good Clinical Practice. *J. Postgrad. Med.* **2001**, *47*, 199–203.
2. Feise, R.J. Do Multiple Outcome Measures Require P-Value Adjustment? *BMC Med. Res. Methodol.* **2002**, *2*, 8.
3. Checkley, W.; Brower, R.G.; Muñoz, A.; NIH Acute Respiratory Distress Syndrome Network Investigators Inference for Mutually Exclusive Competing Events through a Mixture of Generalized Gamma Distributions. *Epidemiology* **2010**, *21*, 557–565.
4. Constantin, J.-M.; Grasso, S.; Chanques, G.; Aufort, S.; Futier, E.; Sebbane, M.; Jung, B.; Gallix, B.; Bazin, J.E.; Rouby, J.-J.; et al. Lung Morphology Predicts Response to Recruitment Maneuver in Patients with Acute Respiratory Distress Syndrome. *Crit. Care Med.* **2010**, *38*, 1108–1117.
5. Puybasset, L.; Gusman, P.; Muller, J.-C.; Cluzel, P.; Coriat, P.; Rouby, J.-J.; and CT Scan ARDS Study Group the Group Regional Distribution of Gas and Tissue in Acute Respiratory Distress Syndrome. III. Consequences for the Effects of Positive End-Expiratory Pressure. *Intensive Care Med.* **2000**, *26*, 1215–1227.
6. Mrozek, S.; Jabaudon, M.; Jaber, S.; Paugam-Burtz, C.; Lefrant, J.-Y.; Rouby, J.-J.; Asehnoune, K.; Allaouchiche, B.; Baldesi, O.; Leone, M.; et al. Elevated Plasma Levels of sRAGE Are Associated With Nonfocal CT-Based Lung Imaging in Patients With ARDS: A Prospective Multicenter Study. *Chest* **2016**, *150*, 998–1007.
7. Calfee, C.S.; Delucchi, K.; Parsons, P.E.; Thompson, B.T.; Ware, L.B.; Matthay, M.A.; NHLBI ARDS Network Subphenotypes in Acute Respiratory Distress Syndrome: Latent Class Analysis of Data from Two Randomised Controlled Trials. *Lancet Respir Med* **2014**, *2*, 611–620.
8. Famous, K.R.; Delucchi, K.; Ware, L.B.; Kangelaris, K.N.; Liu, K.D.; Thompson, B.T.; Calfee, C.S.; ARDS Network Acute Respiratory Distress Syndrome Subphenotypes Respond Differently to Randomized Fluid Management Strategy. *Am. J. Respir. Crit. Care Med.* **2017**, *195*, 331–338.
